# *CDH1* loss remodels gene expression and lineage identity in human mammary epithelial cells

**DOI:** 10.1371/journal.pgen.1012351

**Published:** 2026-10-27

**Authors:** Maggie Musick, Chinasa A. Ufondu, Carmen E. Rowland, Micaela E. Brown, Joseph L. Sottnik, Madeleine T. Shackleford, Camryn S. Nesiba, Etienne P. Danis, Andrew E. Goodspeed, Richard Zera, Arthur T. Johnson, Kathryn L. Schwertfeger, Andrew C. Nelson, Julie H. Ostrander, Matthew J. Sikora

**Affiliations:** 1 Department of Pathology, University of Colorado Anschutz, Aurora, Colorado, United States of America; 2 Department of Pharmacology, Molecular Pharmacology and Therapeutics (MPaT) Program, University of Minnesota, Twin Cities, Minneapolis, Minnesota, United States of America; 3 University of Colorado Cancer Center, University of Colorado Anschutz, Aurora, Colorado, United States of America; 4 Department of Biomedical Informatics, University of Colorado Anschutz, Aurora, Colorado, United States of America; 5 Hennepin County Medical Center, Minneapolis, Minnesota, United States of America; 6 Laboratory Medicine and Pathology, University of Minnesota, Minneapolis, Minnesota, United States of America; 7 Masonic Cancer Center, University of Minnesota, Twin Cities, Minneapolis, Minnesota, United States of America; Emory University School of Medicine, UNITED STATES OF AMERICA

## Abstract

Invasive lobular carcinoma (ILC) is a common subtype of breast cancer, molecularly defined by genetic loss of *CDH1,* and subsequent loss of cell adhesion protein E-cadherin, in ~95% of ILC. Though *CDH1* loss occurs early in ILC oncogenesis, it is unclear how this facilitates transformation. We modeled early *CDH1* loss using “normal” human mammary epithelial cells (HMEC), i.e., finite lifespan cells reflecting early hyperplasia, and targeted E-cadherin signaling using antibodies versus causing genetic *CDH1* loss using siRNA or CRISPR/Cas9-knockout. Transcriptome analysis across four HMEC models showed that the mode of E-cadherin targeting is critical for the subsequent phenotype. Antibody-mediated inhibition of cell-cell contacts induced gene signatures of epithelial-mesenchymal transition (EMT), consistent with the role of E-cadherin suppression during the EMT process. Conversely, genetic *CDH1* loss – as in ILC oncogenesis – repressed EMT signatures, and instead remodeled gene expression toward a luminal epithelial phenotype. RNA-seq, single cell transcriptomics, flow cytometry, microscopy, and ATAC-seq analyses support that *CDH1* loss induces lineage remodeling to a luminal state, which is mirrored in transcriptomic analysis of clinical ILC precursor lesions. By isolating luminal versus basal cells prior to *CDH1* knockout, we found that *CDH1* loss led to remodeling of lineage identity in both populations, converging on a new lineage homeostasis with a luminal progenitor-like phenotype. Consistent with the shift to a luminal progenitor phenotype, *CDH1* loss enhanced proliferative capacity over the finite lifespan of the HMECs, highlighting a feature of early *CDH1* loss that may contribute to clonal advantage during tumor initiation. Moreover, *CDH1* loss enhanced anoikis resistance, a defining feature of ILC cells. Our findings support that genetic loss of *CDH1* in mammary epithelial cells induces transcriptional and phenotypic changes consistent with lineage identity remodeling toward a luminal progenitor-like state, which may underpin the mechanism by which early *CDH1* loss mediates ILC oncogenesis.

## Author summary

Invasive lobular carcinoma (ILC) is a unique form of breast cancer that accounts for approximately 15 percent of all breast cancer diagnoses. A key molecular hallmark of ILC is the genetic loss of the gene encoding E-cadherin, *CDH1*. Though *CDH1* loss is one of the earliest events in ILC oncogenesis, it remains poorly understood how *CDH1* loss permits transformation from normal mammary cells to ILC. To address this gap, we developed cell culture models of early *CDH1* loss using “normal” human mammary epithelial cells (HMEC), i.e., non-transformed mammary cells from healthy reduction mammoplasties reflecting early hyperplasia, and assessed cellular phenotypes upon *CDH1* loss. We identified that the genetic loss of *CDH1*, as opposed to blocking E-cadherin protein, remodeled cellular gene expression and drove a reprogramming of cell lineage identity, with cells adopting a progenitor-like phenotype upon *CDH1* loss and acquiring ILC-like phenotypes. These data provide new mechanistic insight into how loss of *CDH1* is a key oncogenic event in ILC, and may explain distinct aspects of the clinical presentation of ILC among breast cancers.

## Introduction

Invasive lobular carcinoma (ILC) is the most common special histological subtype of breast cancer, accounting for ~15% of all new diagnoses. ILC is molecularly identified by loss of the cell adhesion protein E-cadherin, encoded by *CDH1*, which in ILC is typically via *CDH1* loss-of-heterozygosity (e.g., *CDH1* loss-of-function mutation with a 16q22.1 deletion) [1]. *CDH1* loss is associated with ILC’s characteristic “single-file” discohesive growth pattern in the breast and unique metastatic spread [2–5], and anoikis resistance, i.e., anchorage-independent survival, another hallmark of ILC [6,7]. Importantly, *CDH1* loss is an early event in ILC oncogenesis found in a majority of ILC precursor lesions; ~ 95% of atypical lobular hyperplasia (ALH) and lobular carcinoma *in situ* (LCIS) lesions are E-cadherin-negative [8–11]. However, clinical disease progression in ILC is poorly understood. Though ALH and LCIS are associated with >4-fold and >10-fold increase in breast cancer risk, respectively, clinical management of benign ILC precursors lacks clear guidelines and patients face uncertainty with their long-term risks [8]. Given the central role of *CDH1* in ILC oncogenesis, understanding mechanisms of cancer progression upon *CDH1* loss can improve patient risk assessment and provide new mechanistic insight into the role of *CDH1* as a tumor suppressor.

*CDH1* encodes E-cadherin, a cell surface adhesion protein with critical roles in epithelial cell-cell communication and pathologic contexts in epithelial-mesenchymal transition (EMT), invasion, and metastasis. Though *CDH1* is a tumor suppressor (e.g., hereditary *CDH1* mutations are linked to gastric cancer and ILC [12]), E-cadherin loss or suppression has differing context-specific implications. In breast cancer of no special type (NST; i.e., invasive ductal carcinoma, IDC), E-cadherin is critical for metastasis [13,14]; transient loss of E-cadherin occurs in late stages of cancer progression via epigenetic silencing (e.g., hypermethylation) [15,16], but E-cadherin must be re-activated at metastatic sites to facilitate outgrowth [13,17]. Though E-cadherin suppression is a hallmark of EMT, E-cadherin/*CDH1* loss in ILC is not accompanied by other canonical features of EMT [18], and other studies support that E-cadherin loss leads to induction of EMT specifically in transformed settings [19–21]. Importantly, unlike in EMT, *CDH1* loss in ILC is neither transient nor reversible, thus is mechanistically and phenotypically distinct from EMT-related E-cadherin suppression.

Despite being originally identified in the breast lobules, the cell-of-origin for ILC within the mammary gland has not been explicitly defined. The mammary gland consists of two main epithelial lineages: luminal cells making up the inner lining of the duct, consisting of hormone-responsive and secretory cells, and basal (or myoepithelial) cells lining the duct [22–24]. E-cadherin is expressed in both populations and is critical to maintaining organization in mammary ducts [25,26]. Whether these lineages each differentially support ILC oncogenesis is unclear. However, ILC predominantly present luminal features without classic basal cytokeratins [27,28]) and are largely restricted to luminal-type tumors (i.e., ~ 95% of ILC are estrogen receptor α /ER-positive [29]). The potential relevance of lineage is also reflected in genetically engineered mouse models (GEMMs; Cre/l*oxP, Cdh1*^flox/flox^ plus additional oncogenic hits) [30], as not all Cre-driving promoters drive ILC-like oncogenesis**.** Cre driven by the whey acidic protein promoter (*Wap*; mammary secretory/luminal epithelial cells) or the cytokeratin 14 promoter (*Krt14*; broadly active in epithelial cells) both facilitate ILC-like mammary oncogenesis [19,31]. However, Cre driven by the *Lgr6* promoter (active in epithelial stem cells) did not permit ILC-like oncogenesis, instead producing NST/IDC-like mammary tumors with metaplastic squamous features [32]. Taken together, cellular commitment to the epithelial lineage may be important for ILC oncogenesis.

To further examine how genetic loss of *CDH1* facilitates ILC development and functions as a distinct oncogenic event in breast cancer, we modeled different modes of *CDH1*/E-cadherin functional vs genetic suppression in “normal” non-transformed human mammary epithelial cells (HMEC). Strikingly, genetic *CDH1* loss in these models of early hyperplasia drove a distinct form of phenotypic remodeling compared to functional E-cadherin suppression, which we explored further. This approach ultimately points toward a key role for *CDH1* loss in creating a cellular pathway for ILC oncogenesis.

## Results

### Genetic loss of E-cadherin represses EMT and shifts cells to a luminal transcriptional state

To study the role of *CDH1* loss as a driver of ILC oncogenesis, we used finite lifespan human mammary epithelial cells (HMEC) derived from reduction mammoplasties from healthy individual donors, which each maintain distinct heterogeneous populations of luminal/basal cells [33]. We used variant HMECs that were altered in culture to overcome stasis (i.e., stress-associated senescence barrier to continuous proliferation) via overexpression of *CCND1* [34,35]. *CCND1* is a common copy number gain in ILC (~40% of ILC [36]), thus making these models of early hyperplasia relevant settings for targeting *CDH1* (while facilitating genetic studies, versus using primary cells). HMEC models used in our studies were from donors 122, 153, 184, and 240 (see Methods for specific model definitions) [33–35], and herein are referred to as 122D1, 153D1, 184D1, and 240D1, respectively, to denote *CCND1*-overexpression.

We used three modes of E-cadherin suppression across the four HMEC models: 1) inhibition of E-cadherin using a monoclonal antibody that binds the extracellular domain of E-cadherin, HECD-1 (α-Ecad); 2) *CDH1* siRNA (si*CDH1*); 3) doxycycline-inducible CRISPR/Cas9 knockout of *CDH1* (*CDH1-*KO) (Fig 1A). For *CDH1*-KO models, we used a non-targeting gRNA (NTG) control and two *CDH1*-targeting gRNA (*CDH1*-KO g1 and g2). *CDH1*-KO was generated by two weeks of Cas9 activation to induce frameshift mutations in *CDH1*, without clonal selection post-knockout to maintain HMEC lineage heterogeneity. Of note, each of these approaches also represents a distinct timescale for the perturbation; α-Ecad is acute whereas the genetic models occur over longer timescales that may allow for greater cellular adaptation (Fig 1A). In evaluating *CDH1*/E-cadherin targeting efficacy, α-Ecad caused HMEC to have a rounded morphology, blocked monolayer formation, and reduced the binding efficiency to E-cadherin-coated plates (S1A Fig). siRNA reduced E-cadherin by ~70% by western blot (S1B-S1C Fig). CRISPR/Cas9 knockout of *CDH1* was confirmed through flow analysis, complete knockout was observed in up to ~54% of cells with the remainder showing reduced levels consistent with hemizygous knockout (S1D-S1G Fig).

**Fig 1:**
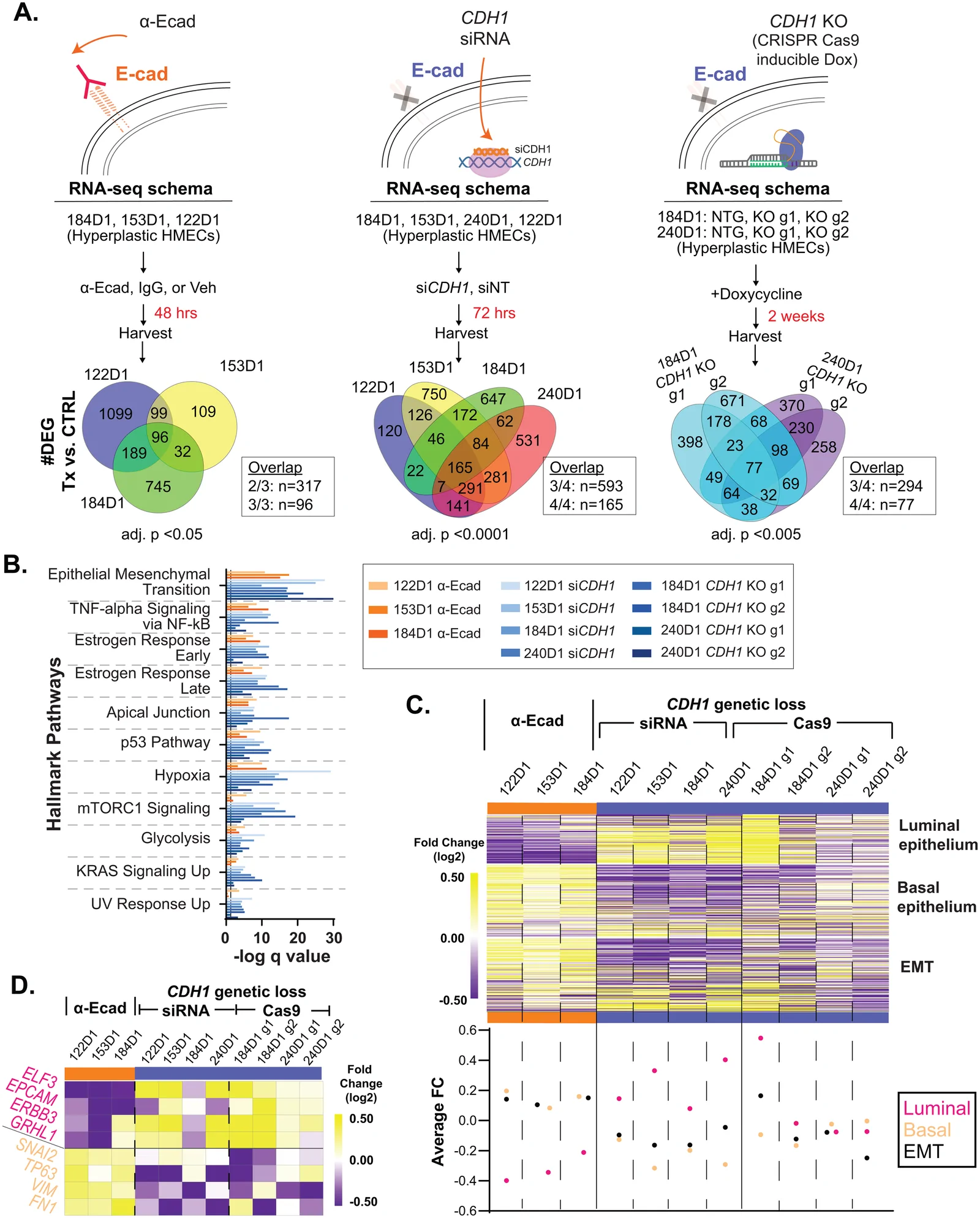
Genetic loss of E-cadherin represses EMT and shifts cells to a luminal transcriptional state. **A)** Workflow of RNA-seq using three methods of E-cadherin suppression; extracellular antibody inhibition (α-Ecad), siRNA transfection (si*CDH1*), and dox-inducible CRISPR/Cas9 (*CDH1*-KO). siNT, non-targeting siRNA. NTG, KO g1, and KO g2 refer to non-targeting Cas9 gRNA, CDH1-targeting gRNA-1, and CDH1-targeting gRNA-2, respectively (see Methods). Venn diagrams show the overlap of differentially expressed genes (DEG), and the adj. p value cutoff used to determine gene lists used for B-E. Adj.p cutoffs were selected in each method to obtain similar numbers of DEGs to prevent biases in downstream gene set analyses from longer vs shorter DEG genesets. **B)** Overrepresentation analysis of Hallmark pathways in all RNA-seq datasets (q value < 0.0001 across genetic loss datasets. Color coded by contact inhibition (orange) vs. genetic loss of *CDH1* (blue). **C)** Fold change of gene expression (p < 0.05) between E-cadherin inhibition and genetic *CDH1* loss within each HMEC line in the EMT Hallmark, luminal epithelium, and basal epithelium gene signatures. Below heatmap: average fold change of each gene signature across cell lines. **D)** Fold changes of representative luminal and basal/EMT genes. K-means clusters are represented by the orange and blue bars (Euclidean distance, 2 clusters, max. iterations = 1000).

We performed RNA-seq using all three modes of E-cadherin loss (Fig 1A), which identified 335-1,915 differentially expressed genes per HMEC model and targeting mode (S1 File). Differentially expressed genes were most strongly enriched in all the models for the Hallmark [37] signature for EMT, and universally enriched for Hallmarks for apical junction signaling, estrogen response, hypoxia, and glycolysis, among others (Fig 1B). We examined these pathways further using fast gene set enrichment analysis (FGSEA) and observed that antibody inhibition of E-cadherin resulted in positive regulation of hypoxia and EMT (S2-S3 Fig), but in contrast, the models of genetic *CDH1* loss showed negative regulation of hypoxia and EMT.

We further examined changes in EMT and lineage signatures upon *CDH1* loss, using signatures for luminal versus basal epithelial phenotype previously developed through gene expression profiling of these HMEC models (~300 genes total) [35]. *CDH1* loss by siRNA and CRISPR/Cas9 increased luminal-associated gene expression and decreased basal-associated gene expression, but the opposite was observed with antibody inhibition of E-cadherin (Fig 1C-1D). Importantly, these trends were observed across all 4 of the HMEC models utilized, which each carry distinct baseline luminal/basal composition (S4A Fig). In our *CDH1*-KO models, the apparent modest change in lineage signatures in HMEC 240D1 (vs 184D1) likely reflects this differing baseline lineage composition; 240D1 are ~ 60% EpCAM^Hi^ luminal cells, whereas 184D1 are > 70% EpCAM^Lo^ basal cells. The relative ratio of luminal to basal/EMT genes from Fig 1D shows that the HMEC 184D1 and 240D1 converge on similar luminal/basal ratios upon Cas9-mediated *CDH1* loss (S4B Fig). These data show that genetic loss of *CDH1* in HMEC mirroring the loss of *CDH1* during ILC oncogenesis is distinct from loss of cell-cell contacts by E-cadherin (i.e., by antibody inhibition); the latter activates EMT [21], while the former represses EMT and pushes cells to a luminal transcriptional phenotype.

### Single cell analysis supports *CDH1*-KO-induced reprogramming in luminal and basal cells

To track the impact of *CDH1* loss within luminal/basal subpopulations in HMEC, we used single-cell RNA sequencing by Particle-templated instant partition sequencing (PIP-seq [38]). We compared HMEC 184D1 non-targeting gRNA control (NTG) versus *CDH1*-KO g2 (referred to as *CDH1*-KO in this analysis), obtaining transcriptome data from 36,956 cells (17,306 NTG, 19,650 *CDH1*-KO), with an average of ~14,000 transcripts per cell covering ~3,800 genes per cell (Fig 2A). As in bulk RNA-seq (Fig 1), the Hallmark EMT signature was repressed across *CDH1*-KO versus control cells (NES = -1.81; adj. p = 0.038). Cell cycle state prediction (i.e., imputed from gene expression data) showed an increase in proliferating (S and G2/M) cells in the *CDH1*-KO population (Fig 2B).

**Fig 2:**
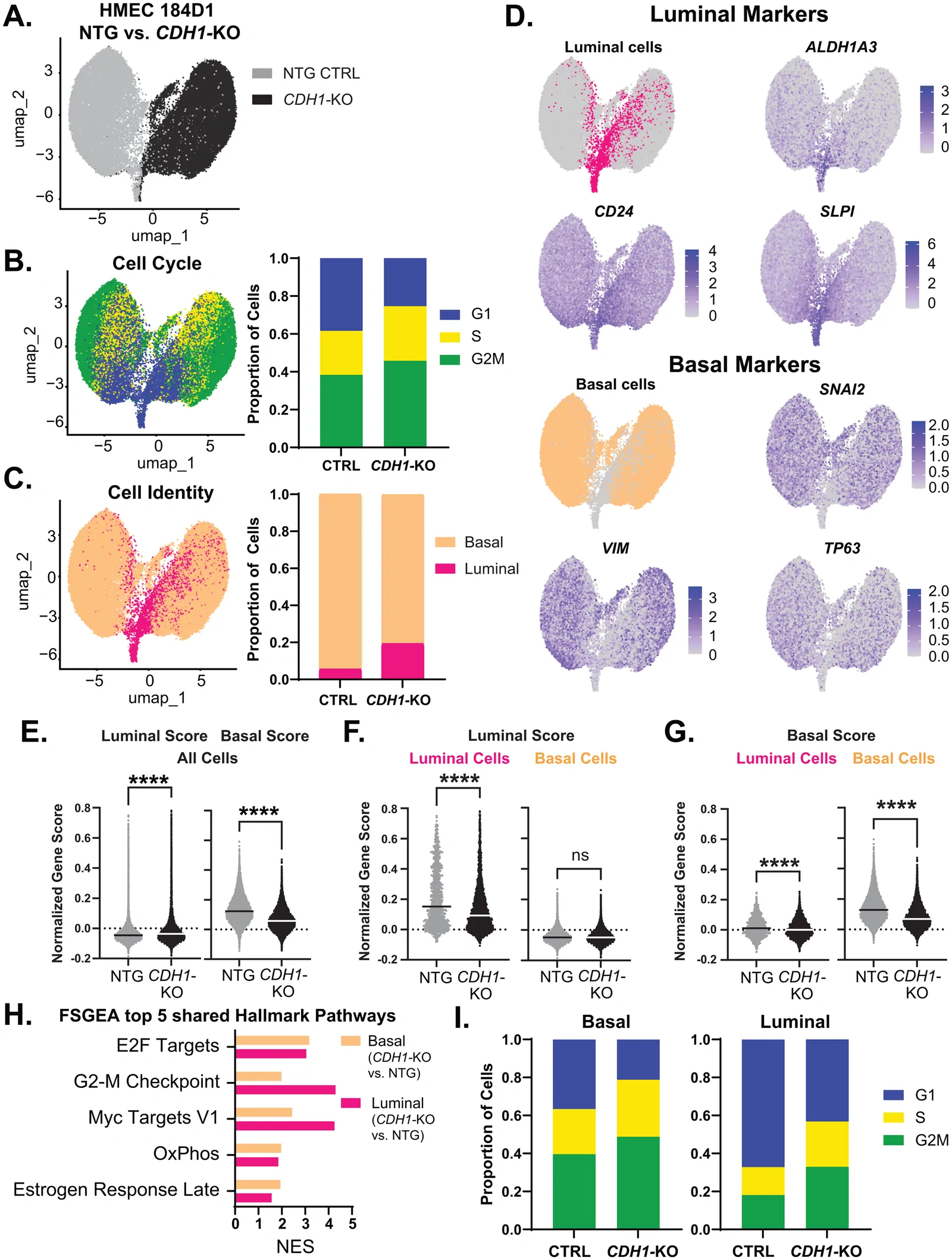
Single cell analysis supports *CDH1*-KO-induced reprogramming in luminal and basal cells. **A)** HMEC 184D1 *CDH1*-KO g2 (*CDH1*-KO) and HMEC 184D1 NTG UMAP clustering based on sample type. ~ 17,000 cells per sample. **B)** Cells characterized by cell cycle signatures; proportion of cells in each phase by sample type. **C)** Using z scores of luminal and basal epithelium gene signatures and the AddModuleScore() function in Seurat, cells were classified as luminal (pink) or basal (orange); proportion of cells within each sample type. **D)** Feature plots of representative luminal (*SLPI, CD24, ALDH1A3*) and basal (*VIM, SNAI2, TP63*) markers. **(E-G)** The distribution of the normalized gene scores calculated using PIPseeker binning algorithm for both the luminal and basal gene signatures: (E) within all cells (F) luminal scores of the luminal and basal cells (G) basal scores of the luminal and basal cells. **H)** FGSEA of the top 5 shared Hallmark pathways for luminal (*CDH1*-KO vs. NTG) and basal (*CDH1*-KO vs. NTG). **I)** Proportion of cells in each cell cycle phase by cell type. -0.3 > logFC > 0.3, adj. p < 1x10^-10^ (pathways p < 0.05; except the luminal Estrogen Response Late p = 0.055).

We then characterized cells as luminal versus basal using the HMEC signatures described above [35]. *CDH1* loss drove a ~ 3.5-fold increase in the luminal population (Fig 2C-2D). Accordingly, mean basal signature score decreased across *CDH1*-KO cells while mean luminal signature score increased (Fig 2E). However, changes observed within the luminal and basal subpopulations suggest that *CDH1* loss reduced the fidelity of luminal vs basal lineage identity; i.e., we observed less “polarization” of luminal vs basal features, as indicated by a decreased luminal score in luminal cells (Fig 2F) and a decreased basal score in basal cells (Fig 2G). This reduced lineage fidelity was seen by other mammary cell lineage signatures (S5 Fig). Other gene expression features were similarly regulated upon *CDH1* loss in both luminal and basal subpopulations (Fig 2H), e.g. Hallmark signatures for oxidative phosphorylation and estrogen response were upregulated in both luminal and basal cells, and cell cycle imputation from gene expression data showed an increase in proliferative cells (S and G2/M) in both lineage populations (Fig 2I). We isolated cells predicted to be in G1 to exclude these proliferation features; among the most induced genes by *CDH1*-KO were cytokeratin 16 (*KRT16*) and transcription factor inhibitor of DNA binding 2 (*ID2*) (S6A-S6B Fig), both of which are associated with luminal progenitor cells [6,39]. Notably, in pathway analyses via FGSEA, the cholesterol homeostasis Hallmark was upregulated in *CDH1*-KO cells in both luminal and basal populations (luminal G1 NES = 2.48, adj. p = 0.0027; basal G1 NES = 1.71, adj. p = 0.0706) (S6A, S6C Fig). This single-cell analysis supports observations from bulk RNA-seq that *CDH1* loss drives a population-level shift to a luminal epithelial phenotype, and identifies a loss of strict lineage identity within luminal and basal populations and a gain of features of luminal progenitor cells.

### Chromatin landscape supports *CDH1* loss-induced changes in cell identity

Since cell lineage is closely tied to chromatin accessibility [40–42], we examined changes in the chromatin landscape caused by *CDH1* loss via ATAC-seq (Assay for Transposase-Accessible Chromatin Sequencing), using siRNA in HMEC 240D1 and 153D1. The majority of accessible chromatin sites (i.e., peaks) in both models were unchanged by *CDH1* knockdown (Fig 3A-3B); gained peaks (chromatin opened by *CDH1* loss) accounted for 2.1% and 6.3% of all peaks in 240D1 and 153D1, respectively, and reduced/lost peaks (chromatin closed by *CDH1* loss) accounted for 2.5% and 8.2% of all peaks in 240D1 and 153D1, respectively (Fig 3A-3B). The distribution of maintained and gained peaks were similar in introns (~40%), distal intergenic regions (~16%), and promoters (~37%). However, lost peaks had a higher distribution of peaks found in introns (49–52%) and distal intergenic regions (18–23%) and reduced distribution of peaks found in promoters (18–26%) compared to maintained and gained peak distributions (S7A Fig). Between the HMEC models, we see direct overlap of 566 gained peaks and 573 lost peaks (Fig 3C), which were within 30kb of 513 and 363 genes, respectively (Fig 3D), suggesting that these genes were directly regulated by changes in chromatin accessibility upon *CDH1* loss. Accordingly, 165 of the genes identified in ATAC-seq were differentially expressed after *CDH1* knockdown in both HMEC models (153D1 and 240D1, using a cutoff value of adj. p < 0.05 for differential gene expression) (Fig 3D-3E). Genes associated with gained peaks had significantly higher mean fold change compared to the genes associated with lost peaks (S7B-S7C Fig) and included luminal breast cancer-associated markers (*KCNK5, PDK4, CPT1A*) [43–46] (Fig 3E-3F, S2-S3 Files); genes associated with lost peaks were mostly downregulated and included basal and EMT-related factors such as *SERPINE1*, *SPHK1*, ZFHX4, *LAMC2*, and *NRG1* (Fig 3E-3F, S2-S3 Files) [47–51]. In both HMEC models, there was a significant positive correlation between fold changes in ATAC-seq peak signal versus fold changes in corresponding gene expression (Figs 3F, S7B). The direct association between chromatin landscape and gene expression supports that *CDH1* loss drives epigenomic reprogramming toward a luminal progenitor-like phenotype.

**Fig 3:**
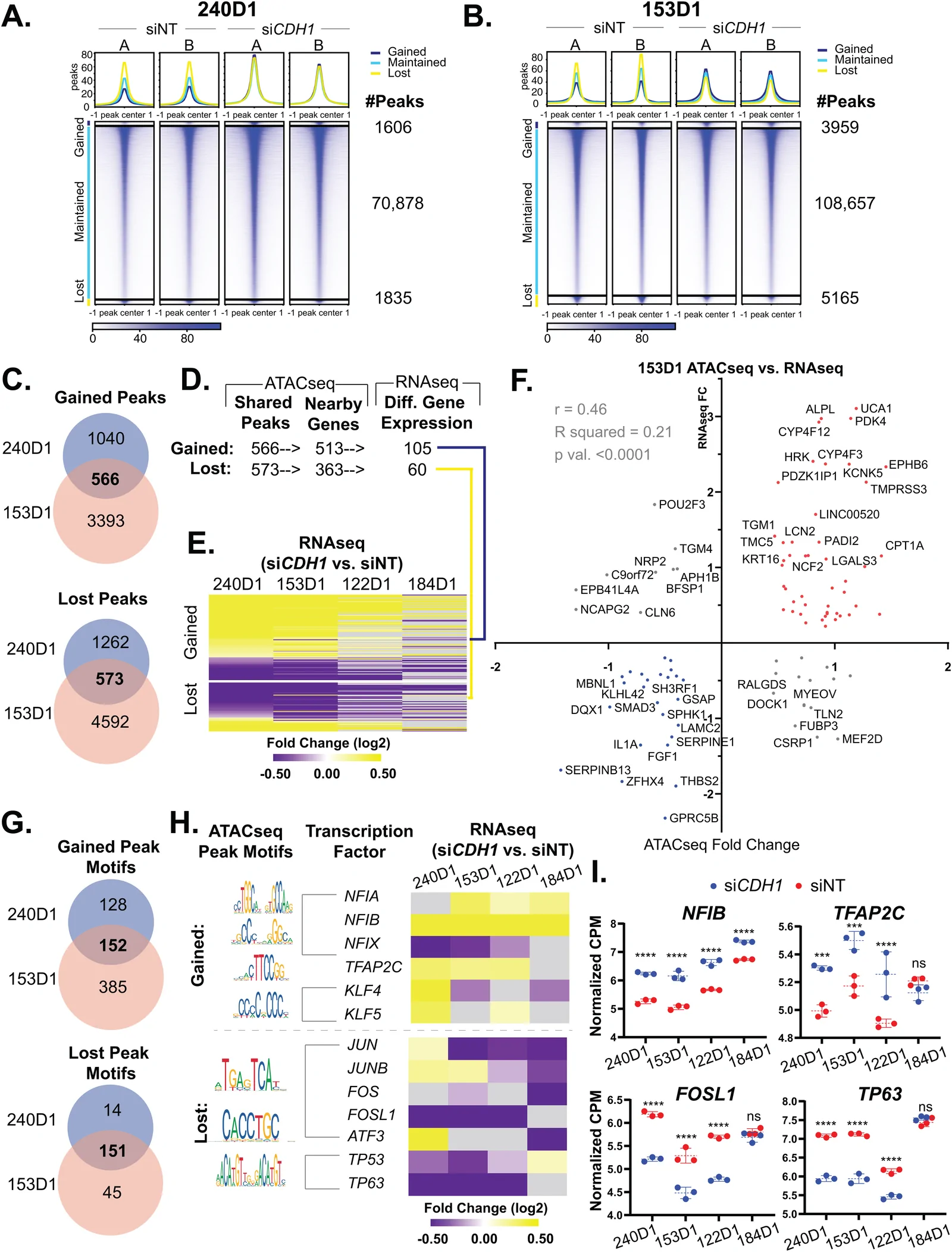
*CDH1*-KO chromatin landscape supports change in cell identity. A-B) Tornado plots centered at peak centers (+/-1kb) arranged by gained, maintained, and lost peak clusters following si*CDH1*. There are two sets of tornado plots showing the individual replicates of si*CDH1* and siNT for HMEC cell line 240D1 (A) and 153D1 **(B)**. The expression bars below the tornado plots show the relative peak intensity per cell line. Peak plots above each tornado plot show the distribution of peaks per cluster within each sample/replicate. In the HMEC 240D1, there were 74,319 reproducible peaks and of these peaks there were 70,878 maintained peaks, 1,606 gained peaks, and 1,835 lost peaks. In the HMEC 153D1, there were 117,781 reproducible peaks and of these peaks there were 108,657 maintained peaks, 3,959 gained peaks, and 5,165 lost peaks. **C)** Venn diagrams show the overlap of gained and lost peaks between cell lines. **D)** Peak to gene analysis (BETA-minus pipeline, see Methods) was used to identify nearby genes within 30kb of the shared peaks. 165 of these genes were differentially expressed in the RNAseq datasets from 240D1 and 153D1 HMEC models using an adj. p < 0.05. **E)** Heatmaps show log2 fold change (si*CDH1* vs. siNT) of all differentially expressed genes within each of the four HMEC models. Light gray represents non-significant DGE. **F)** Scatter plot of fold changes from differential peak analysis vs. corresponding gene expression from RNAseq within the 153D1 cell line. Red circles show genes that are upregulated and associated with gained peaks and blue circles show genes that are downregulated and associated with lost peaks. **G)** ATACseq peak motif analysis (i-cisTarget) was used to identify motifs associated with gained and lost peaks within each cell line (NES > 3). Venn diagrams show the overlap of motifs in gained and lost peaks across cell lines. **H)** Summary of motifs (JASPAR) and corresponding transcription factors organized by motifs associated with gained peaks (top) followed by lost peaks (bottom). Heatmaps show log2 fold change (si*CDH1* vs. siNT) of the transcription factors from the RNAseq datasets of all four HMEC cell lines (adj. p val. < 0.05). **I)** RNAseq Normalized counts per million (CPM) for each replicate for each condition (siNT, n = 3 and siCDH1, n = 3) within each HMEC cell model were plotted for the top transcription factors from **H.** (adj. p val **** < 0.0001; *** < 0.0005).

To identify potential drivers of the changes in chromatin accessibility after *CDH1* loss, we examined transcription factor motifs enriched in gained versus lost chromatin accessibility sites [52]. There were 152 shared motifs associated with gained peaks and 151 motifs associated with lost peaks (Fig 3G, S3 File). Among the top motif families were NFI-related motifs including (NFIA, NFIB, and NFIX), which are indicative of a luminal progenitor state, ETS-related motif TFAP2C, and epithelial identity motif KLF4 and progenitor-associated motif KLF5 [53–57] (Fig 3H). The gene expression of these factors were mostly upregulated across all four HMEC models following *CDH1* loss; however, there was variability across HMEC models (Fig 3H). There was no overlap in motifs between gained and lost peaks. The motifs associated with the lost peaks include basal AP1-related motifs JUN, FOS and ATF3 and basal/EMT-related motif TP63 [47,58]. The loss of these motifs in ATAC-seq were accompanied by downregulation of their transcripts following *CDH1* loss (Fig 3H). Although si*CDH1* in the 184D1 HMECs did not have statistically significant differential gene expression changes for several of the top transcription factors, we found that gene expression (in counts per million) in si*CDH1* 184D1s was similar to si*CDH1* gene expression levels from the other three cell models (Fig 3I). Further, *TP63* expression decreased in *CDH1*-KO vs. NTG in the 184D1 model (-0.20 to -0.48 log2 FC). We also examined differentially expressed genes in 184D1 *CDH1*-KO models for enrichment in regulatory genesets from ENCODE/ChEA, which includes consensus genesets for TP53, TP63, KLF4, FOS, and ATF3; TP63, FOS, and KLF4 were each significantly enriched in differentially expressed genes for both gRNA knockouts (S3 File), supporting the motif enrichment observed in ATAC-seq. This analysis suggests that shared transcriptional/epigenomic factors are dysregulated across HMECs upon *CDH1* loss.

### *CDH1* loss in HMEC mirrors gene expression changes in early lobular neoplasia

We hypothesized that gene expression changes seen in the transition from normal breast tissue to lobular neoplasia, i.e., ILC precursors atypical lobular hyperplasia (ALH) and lobular carcinoma *in situ* (LCIS), would provide a window into the early changes caused by *CDH1* loss, as >95% of ALH/LCIS are already E-cadherin negative [1,8–11]. However, LCIS gene expression data are extremely limited, with only a single array-based study reported to date [59]. To examine gene expression changes in LCIS, we conducted transcriptomic analyses of archival LCIS tissues from U. Minnesota (n = 22) and unaffected normal breast from reduction mammoplasties (n = 7) by RNA-seq (clinicopathologic features and gene expression summaries in S4 File). Based on our observations in HMEC, we examined expression of the Luminal/Basal signatures as above and found that these genes drove independent clustering of the normal vs LCIS tissues (Fig 4A). Of note, 4 of 22 LCIS were considered to have pleomorphic histology. While pleomorphic LCIS (pLCIS) clustered with classic LCIS (n = 18) by these lineage markers, pLCIS likely represents a distinct oncogenic trajectory [60,61], and as such were excluded in further analysis. Among lineage markers, key luminal markers (*MUC1, FOXA1, KRT18, KRT8, ESR1*) were increased in LCIS, and basal markers (*KRT5, KRT14, ITGA6, TP63*) were decreased in LCIS compared to normal breast tissue **(**Fig 4B). We further examined differentially expressed genes between classic LCIS and normal tissues (n = 1455 genes at adj.p < 0.01; S4 File). By gene set enrichment, the Hallmark signatures for Estrogen Response were the most strongly activated in LCIS, whereas Apical Junction was suppressed, consistent with remodeled cell adhesion and cell surface markers with *CDH1* loss (Fig 4C; S4 File). Changes in other gene sets for luminal vs. basal / mesenchymal lineage [62] were consistent with a luminal-type cell lineage shift in LCIS relative to normal tissue **(**Fig 4C; S4 File). Importantly, the observed gene expression differences are correlative with *CDH1* loss in early lobular neoplasia, yet may reflect other features of oncogenesis, e.g., increased epithelial cellularity in LCIS. However, the shift we observe in LCIS contrasts data from ductal carcinoma *in situ* (DCIS) in the literature; Rebbeck et al assessed gene expression in samples from n = 145 patients with DCIS, and identified EMT activation in the progression of DCIS to invasive disease [63]. Taken together, LCIS gene expression data are consistent with the changes seen in our *CDH1* loss models in HMEC, and support that early ILC oncogenesis includes a cell lineage shift to a luminal phenotype.

**Fig 4:**
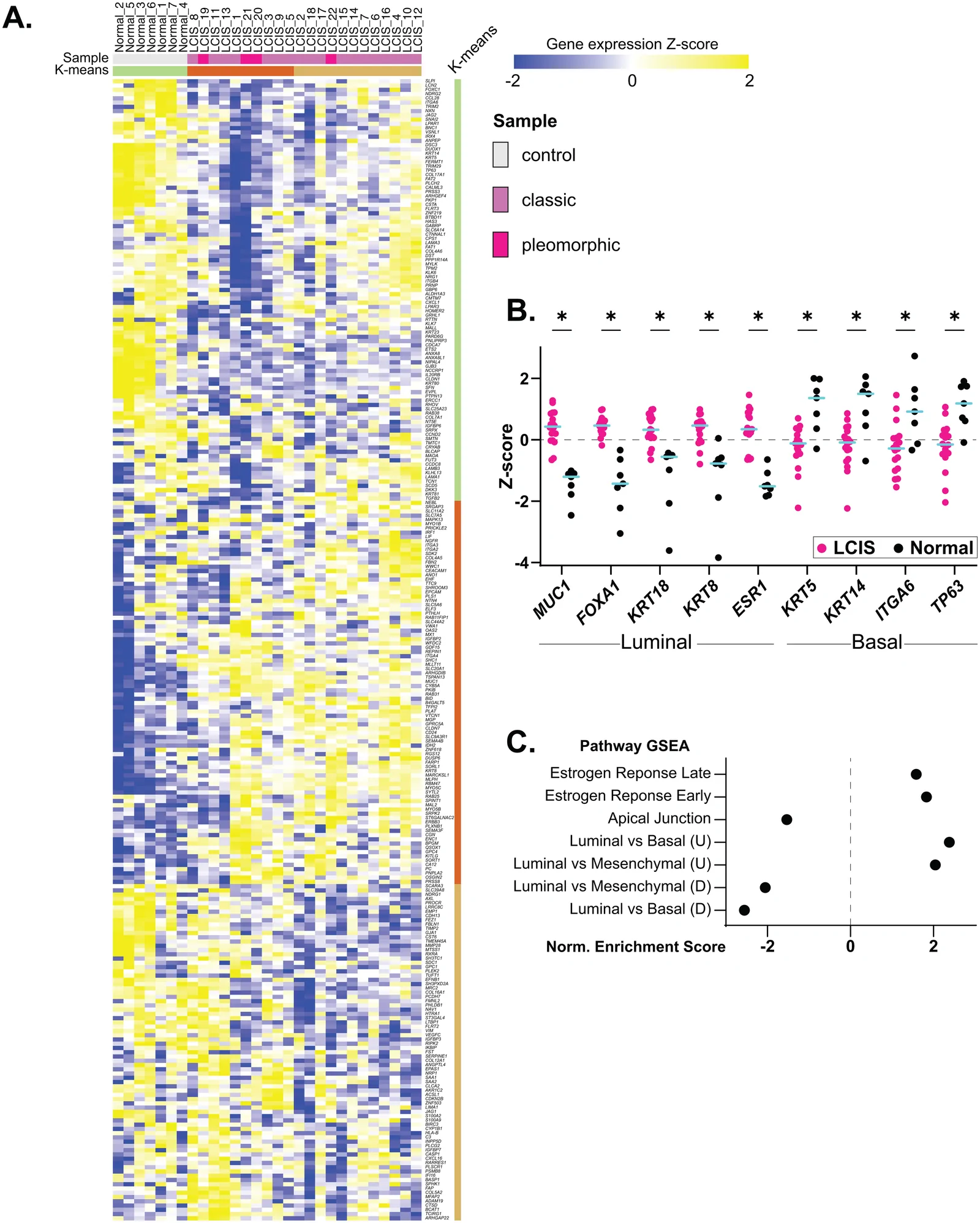
*CDH1*-KO mimics luminal shift in LCIS data. **A)** RNA-sequencing on archival tissues from **U.** Minnesota, from LCIS (n = 22) vs. unaffected normal breast from reduction mammoplasties (n = 7). Heatmap shows luminal and basal genes and K means clusters (represented by green, orange, and yellow bars). **B)** Z-scores of key luminal and basal markers comparing LCIS vs. normal breast. *p val. < 0.01. **C)** Pathway gene set enrichment analysis of Hallmark gene signatures (Estrogen Response Early and Late, Apical Junction) and other gene signatures (Charafe Luminal vs. Basal, Luminal vs. Mesenchymal). Adj. p val. < 0.05.

### Cellular lineage markers support a *CDH1* loss-induced luminal progenitor-like phenotype

We next examined how gene expression changes upon *CDH1* loss would lead to changes in cell surface presentation of lineage marker proteins, thus indicating a lineage phenotype change. Flow cytometry analysis of EpCAM versus CD49f (*ITGA6*) was used to define cells as basal (EpCAM^lo^/CD49f^hi^), luminal progenitor (EpCAM^hi^/CD49f^hi^), or mature luminal (EpCAM^hi^/CD49f^lo^) (Fig 5A). Consistent with the lineage shifts we observed by gene expression, *CDH1* loss caused a shift in 184D1 from primarily basal (EpCAM^lo^) to luminal (EpCAM^hi^) phenotype (Fig 5A), with an enrichment in EpCAM^hi^/CD49f^hi^ cells, indicating an enrichment in the luminal progenitor-like population (Fig 5B). We further examined the luminal enrichment using CD10/*MME*, which is typically a basal marker but also indicates progenitor capacity in EpCAM^hi^ cells [64–66]. Across all HMEC and *CDH1* loss models tested, *CDH1* loss increases CD10 expression in the EpCAM^hi^ luminal population (Fig 5C), supporting that these cells acquire progenitor capacity after *CDH1*-KO. We examined this further using the early common progenitor-derived colonies assay [67], wherein colony formation on a layer of irradiated NIH 3T3 mouse fibroblasts, per colony morphology, can distinguish epithelial progenitor capacity as luminal, basal, or mixed. When inducing Cas9/*CDH1*-KO in standard 2D culture and then transferring to the feeder layer, 94% of NTG colonies presented the basal phenotype, whereas 88% of *CDH1*-KO colonies presented the luminal phenotype (Fig 5D-5E). We repeated the experiment by allowing cells to form primary spheres in 3D (ultra-low attachment) after *CDH1*-KO, prior to transfer to the feeder layer. Similarly, 70% of NTG colonies were the basal phenotype, versus none in *CDH1*-KO colonies, where 85% were the luminal phenotype (Fig 5F-5G). Taken together, increased CD10 and the functional shift to luminal phenotype colony formation supports that *CDH1*-KO induces a phenotypic shift to a luminal progenitor-like state.

**Fig 5:**
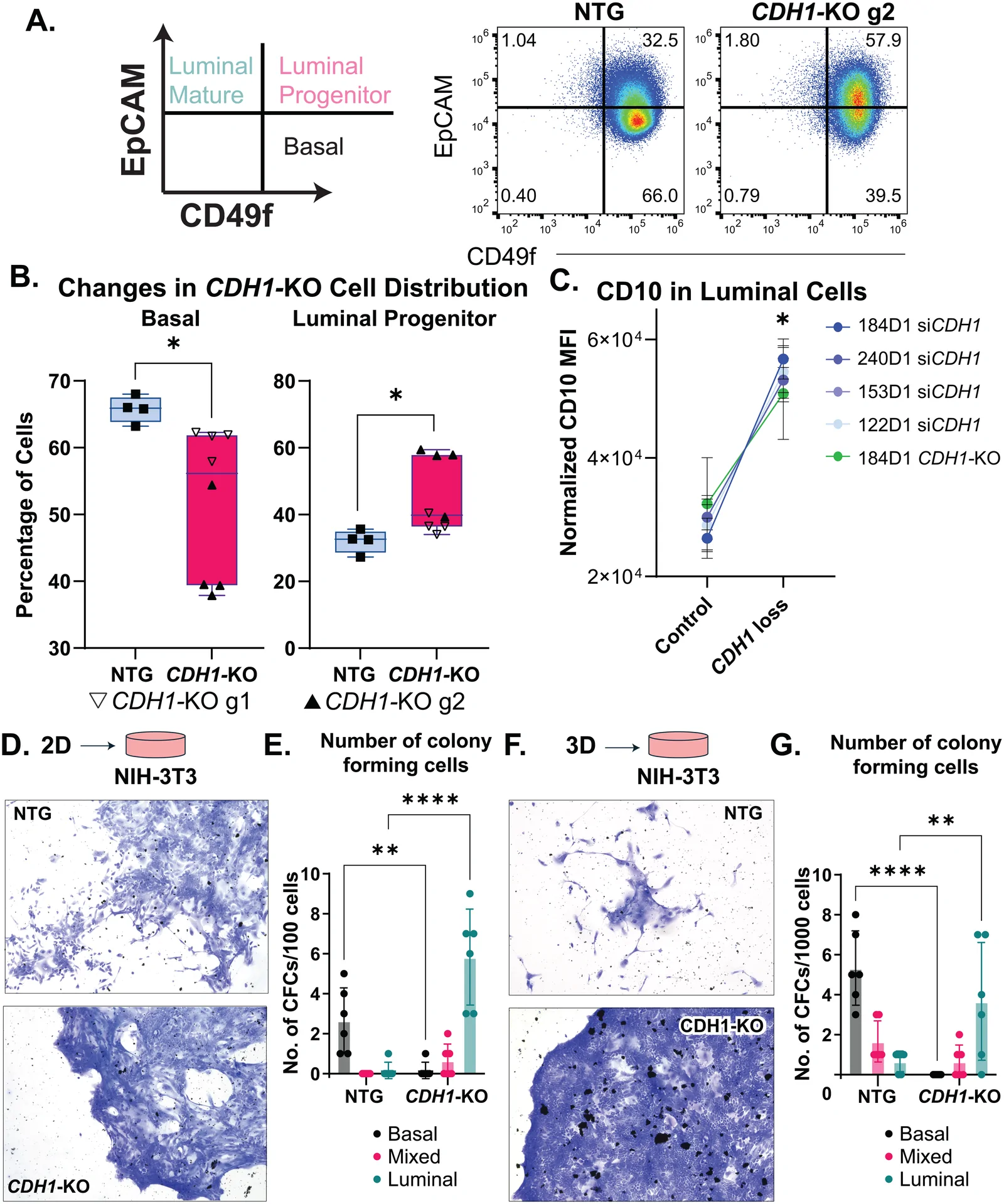
Cellular lineage markers support a *CDH1* loss-induced luminal progenitor-like phenotype. **A)** Representative plots of flow cytometry analysis of EpCAM/CD49f expression in 184D1 NTG vs. *CDH1*-KO. Gates were set to designate cell lineage: mature luminal (EpCAM^hi^ CD49f^low^), luminal progenitor (EpCAM^hi^ CD49f^hi^), and basal (EpCAM^low^ CD49f^hi^). Percentages of cells in each lineage are noted in each quadrant. **B)** Quantification of percentage of basal cells (left) and luminal progenitor cells (right) (n = 8; n = 4 *CDH1*-KO g1; n = 4 *CDH1*-KO g2) *(adj. p val. < 0.05). **C)** Quantification of CD10 expression changes were measured by mean CD10 MFI normalized across multiple experiments (n = 14; n = 5 siCDH1 184D1, n = 3 si*CDH1* 122D1, n = 2 si*CDH1* 240D1, n = 4 si*CDH1* 153D1) or *CDH1*-KO vs. NTG (n = 8; n = 4 *CDH1*-KO g1; n = 4 *CDH1*-KO g2) (adj. p val. * < 0.0006) **D)** HMEC *CDH1*-KO g2 and NTG grown in 2D, transferred to feeder layer of irradiated NIH 3T3 cells. Images of NTG and *CDH1*-KO g2 colonies on NIH-3T3 feeder layer. **E)** Quantification of the number of colony forming cells (CFCs) per 100 cells seeded (2-way ANOVA; adj. p val. **** < 0.0001; ** = 0.0017); represents n = 56 colonies total. **F)** HMEC *CDH1*-KO g2 and NTG grown in 3D, transferred to feeder layer of irradiated NIH 3T3 cells. Images of NTG and *CDH1*-KO g2 colonies on NIH-3T3 feeder layer **G)** Quantification of the number of colony forming cells (CFCs) per 1000 cells seeded (2-way ANOVA; adj. p val. **** < 0.0001; ** = 0.0020).; represents n = 72 colonies total. Additional information on analysis/phenotyping in (D-G) is provided in Methods.

### *CDH1*-KO induces lineage remodeling in both luminal and basal cells

Our data suggest that *CDH1* loss drives epigenomic and transcriptomic changes that subsequently drive lineage phenotype remodeling, yet the lineage shift could be explained by a selection against basal cells by *CDH1* loss. To examine this, we used flow sorting to isolate luminal (EpCAM^hi^) vs basal (EpCAM^lo^) cells from 184D1 prior to inducing *CDH1*-KO (Fig 6A-6B). Twenty-four hours after initial sorting, we confirmed that cells were effectively sorted into luminal vs basal populations (Fig 6C). We then induced *CDH1*-KO in each sorted population over the course of 2 weeks, reassessing lineage markers at 2 weeks and 4 weeks after initiating *CDH1* knockout (Fig 6D). Flow cytometry gating for each analysis was based on respective fluorescence minus one control (gating strategy in S8 Fig). 184D1 cells are predominantly basal/EpCAM^lo^ at baseline (~80%), and both EpCAM^hi^ and EpCAM^lo^ sorted NTG cells re-established this baseline over 2–4 weeks (60–80% basal/ EpCAM^lo^; Fig 6B-6D**, top**). Conversely, in the *CDH1*-KO cells, both EpCAM^hi^ and EpCAM^lo^ sorted cells instead maintained a greater luminal progenitor population (40–60% basal/ EpCAM^lo^; 40–50% EpCAM^hi^/CD49f^hi^; Fig 6B-6D**, bottom**). By 2 weeks post sort, all sorted cells attempted to re-establish HMEC heterogeneity; however, both the EpCAM^hi^ and EpCAM^lo^ *CDH1*-KO populations maintained higher EpCAM levels than the NTG EpCAM^hi^ and EpCAM^lo^ sorted populations (S9A Fig). By 4 weeks post sort, both sorted populations converged at similar EpCAM levels by mean fluorescent intensity (MFI) for *CDH1*-KO versus NTG models (Fig 6E). Together these findings support that *CDH1* loss induces lineage remodeling in both luminal and basal cells, towards a new EpCAM^hi^ (luminal) homeostasis, suggesting that *CDH1* loss in either mammary epithelial population may ultimately facilitate ILC oncogenesis.

**Fig 6:**
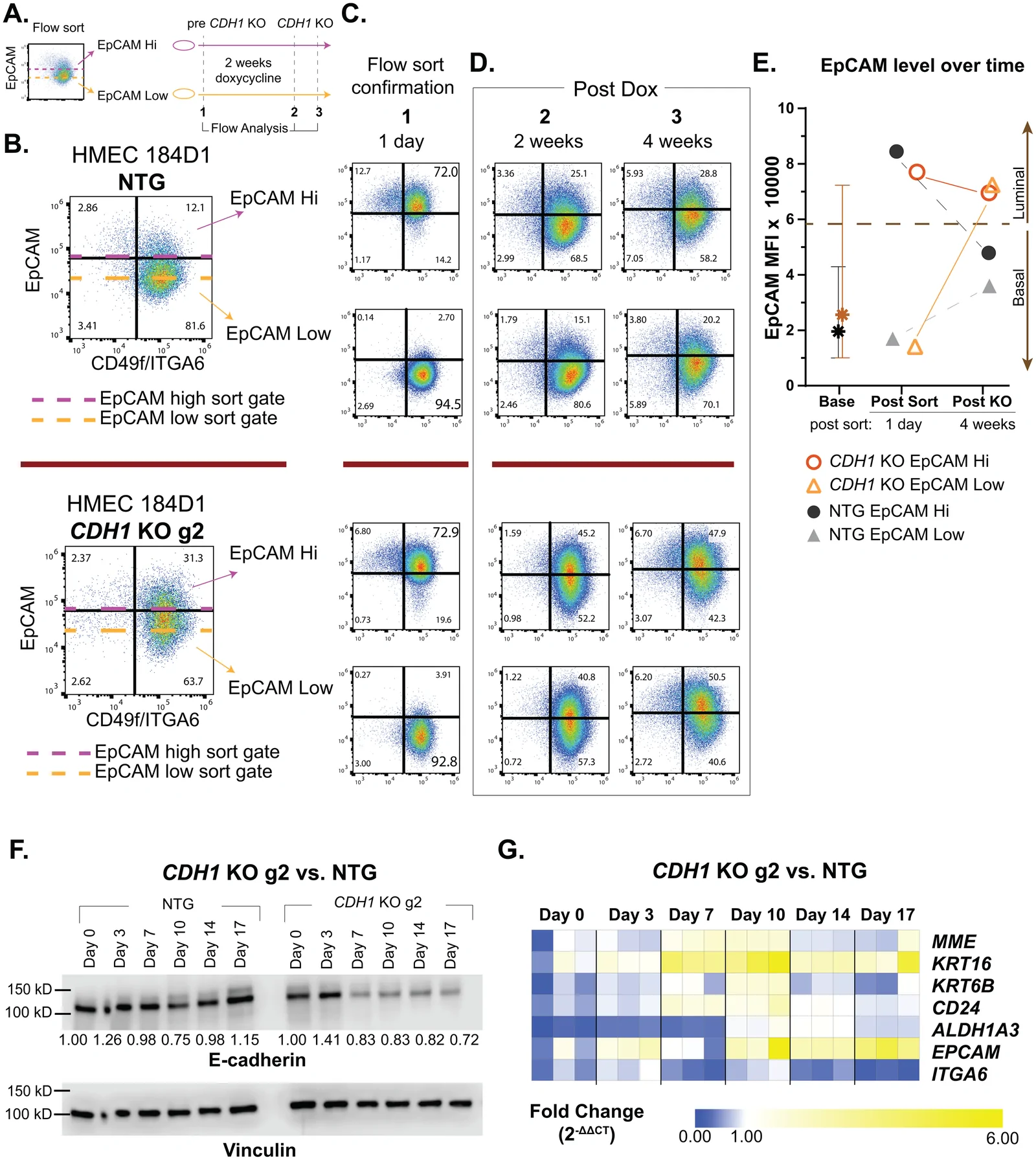
*CDH1*-KO induces lineage remodeling in luminal and basal cells. **A)** Schematic of flow sorting followed by flow analyses before and after induction of *CDH1*-KO. Cells were sorted into two populations using EpCAM: EpCAM hi (luminal) and EpCAM low (basal) and were separately treated with doxycycline. Additional experimental detail is provided in the Methods. **B)** Flow sorting and subsequent flow analyses were conducted in two cell lines: HMEC 184D1 NTG and HMEC 184D1 *CDH1*-KO g2. Dashed lines show where sorting gates were set based off EpCAM expression. C) 1 day post sort (before induction of *CDH1*-KO) the efficiency of the sort was determined by cell distribution based on EpCAM/CD49f expression as described in Fig 4A (n = 1). **D)** After *CDH1*-KO (2 weeks and 4 weeks post dox induction) flow analysis of cell distribution based on EpCAM/CD49f was determined (n = 3; n = 2). See gating FMOs for each flow analysis in S8E Fig) EpCAM MFI (geomean) within parental/pre-sorted population (baseline) and sorted populations before induction of *CDH1*-KO (post-sort) and after *CDH1*-KO (post KO). Baseline population error bars show 10% and 90% distribution of EpCAM MFI. Sorting timecourse data in (D-E) is from an individual experiment; biological triplicate samples at 2 weeks (S9 Fig) were ultimately pooled for 4 week analysis due to sample limitations; data are representative of a series of repeated studies of the overall post-knockout timeframe. **F)** Time course western blots of E-cadherin with Vinculin loading control. Densitometry was calculated by subtracting local background, normalizing to lane-specific loading control (Vinculin), and then normalized to Day 0 NTG or Day 0 *CDH1* KOg2. **G)** Paired time course samples analyzed via qPCR for basal and luminal gene expression.

### *CDH1* loss induces a temporal shift in lineage marker expression

Our models of *CDH1* loss include si*CDH1* as an acute *CDH1* loss over 72 hrs, whereas *CDH1*-KO is a more chronic or long-term context, with 2 weeks between inducing *CDH1*-KO and assay, leaving a gap in our phenotypic analyses. We performed a time course analysis of *CDH1*-KO in 184D1, measuring E-cadherin protein and lineage marker gene expression over 17 days upon Cas9 induction. By Day 7 post-induction, E-cadherin protein reached a stable minimum in both *CDH1*-KO gRNA models (Fig 6F – *CDH1*-KO g2; S9B Fig– *CDH1*-KO g1). Decrease in E-cadherin coincided with changes in lineage marker gene expression (Figs 6G, S9C Fig). Progenitor markers *MME*/CD10, *KRT16*, and *KRT6B* peaked at 7–10 days; *KRT16* remained elevated through the time course, consistent with being a specifically luminal progenitor marker [39]. Luminal progenitor marker *ALDH1A3* peaked at 10–14 days and similarly remained elevated versus baseline at 17 days. Luminal marker *CD24* peaked at 7–10 days then decreased, consistent with a transition to a CD24^lo^ progenitor state [68] after 10 days. *EPCAM* (luminal) and *ITGA6*/CD49f (basal) expression mirrored a transition to the luminal phenotype 10–17 days post-Cas9 induction, increasing and decreasing, respectively, to stable points at day 10–14 and beyond (Figs 6G, S9C Fig). These temporal changes in epithelial lineage phenotype markers support that early gene expression changes captured with acute *CDH1* loss by siRNA drive a phenotypic shift or remodeling to the luminal progenitor phenotype over the course of 10–17 days.

### *CDH1*-KO increases proliferative potential and anoikis resistance

We next examined how *CDH1* loss-induced lineage remodeling corresponds to acquisition of cancer phenotypes, focusing on proliferation and anchorage-independent growth (anoikis resistance), to link lineage remodeling to early oncogenesis. First, we examined proliferation over the finite lifespan of our post-stasis HMEC cultures, comparing NTG vs *CDH1*-KO models, + /- 2 weeks of doxycycline (dox) to induce Cas9. *CDH1*-KO cells had more population doublings throughout their lifespan compared to the NTG populations (Fig 7A). Specifically in the post-induction phase (i.e., after doxycycline completion, passages 17–22), *CDH1*-KO g1 and g2 + dox populations showed higher proliferation rates than –dox controls, whereas there was no difference in NTG controls; *CDH1*-KO g1 and g2 models +dox had 2.3-fold and 2.6-fold higher proliferation rates, respectively, versus NTG + dox (Fig 7A). These data support that *CDH1* loss increases proliferative potential in HMEC, which may support oncogenesis, e.g., driving clonal advantage.

**Fig 7:**
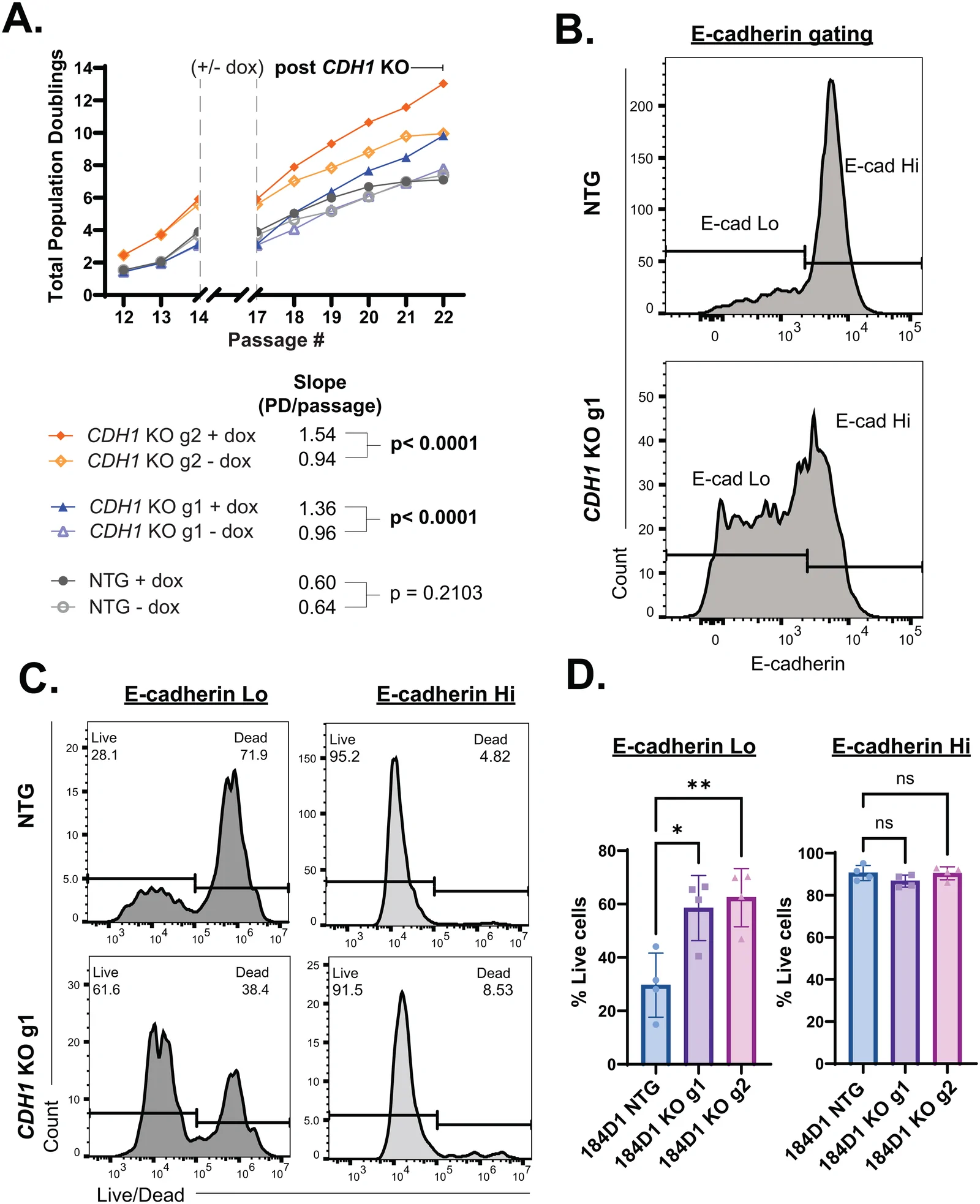
*CDH1*-KO increases proliferative potential and anoikis resistance. **A)** Total population doublings over the lifespan of the HMEC 184D1 NTG, *CDH1*-KO g1, *CDH1*-KO g2, all + /- dox were calculated by counting cells at the beginning and end of every passage (further described in the methods). The + dox cells were treated from passage 14 through passage 17 with doxycycline (population doublings during this period were excluded from final analysis to remove impact of doxycycline on proliferation). Post-induction proliferation rates were calculated using the following equation: Slope = Δ Population Doublings / 5 passages (17 à 22). Sidak’s multiple comparison test was used to calculate statistical significance between the slopes of the + and – dox models. **B)** Anoikis resistance assay, E-cadherin Lo and Hi gating for NTG and *CDH1*-KO cells. **C)** Flow cytometry representative histograms of anoikis resistance using live/dead fixable dye in E-cadherin Lo and E-cadherin Hi populations of NTG and *CDH1*-KO cells. **D)** Quantification of percent live cells as a representation of anoikis resistance in ULA (n = 4) in E-cadherin Lo and E-cadherin Hi populations. P value = 0.0126 (*) and 0.0061 (**) for *CDH1*-KO g1 and g2 respectively; one way ANOVA and Dunnett’s multiple comparisons test.

One of the defining features of ILC is enhanced anoikis resistance, i.e., anchorage-independent cell survival, which has been mechanistically linked to E-cadherin loss [7]. To assess whether *CDH1* loss was sufficient to support anoikis resistance in HMEC, we induced *CDH1*-KO and then seeded cells into ultra-low attachment plates and assessed cell survival based on E-cadherin status using flow cytometry. A subset of *CDH1*-KO cells remain E-cadherin positive (e.g., hemizygous for *CDH1*), while a subset of HMEC as in NTG controls are E-cadherin negative (likely via reduced *CDH1*/E-cadherin in basal cells [69]) **(**Fig 7B**)**, so we hypothesized that specifically *CDH1*-KO would permit anoikis resistance in E-cadherin low cells. Among E-cadherin low cells, < 30% of NTG cells were viable whereas >60% of *CDH1*-KO cells were viable (i.e., anoikis resistant) **(**Fig 7C-7D**)**. The enhanced anoikis resistance of E-cadherin-negative *CDH1*-KO cells likely supports early tumorigenesis, and suggests that *CDH1* loss-driven cellular remodeling, and not simply decreased E-cadherin levels, is a critical feature of ILC oncogenesis.

## Discussion

In this study, we sought to better understand the cellular consequences of early *CDH1* loss in ILC oncogenesis. Through transcriptomics, flow cytometry, and colony formation assays, we demonstrated that *CDH1* loss, distinct from E-cadherin inhibition, remodels human mammary epithelial cell lineage identity toward a luminal progenitor-like phenotype; our orthogonal approaches and associated findings are summarized in S5 File. Notably, *CDH1* loss induces lineage remodeling in both luminal and basal cells; either population in 184D1 cells converged on a distinct EpCAM^hi^ state after *CDH1* loss, but NTG controls restored the baseline EpCAM^lo^ state. Gene expression changes were accompanied by *CDH1* loss-driven changes in chromatin accessibility, which suggested putative factors that mediate gene expression changes upon *CDH1* loss. Importantly, gene expression changes in clinical samples of LCIS, which presented an increase in luminal markers and decrease in basal markers relative to normal breast tissue, were consistent with the gene expression changes seen in HMEC models upon *CDH1* loss that show remodeling toward a luminal lineage phenotype. Functionally, *CDH1* loss increased the proliferative capacity and proliferation rate of HMEC cells and facilitated anoikis resistance within E-cadherin-negative cells, linking lineage remodeling to key phenotypes that are likely critical for ILC oncogenesis.

We compared different modes of E-cadherin suppression and hypothesized that loss of cell-cell contacts alone (using E-cadherin antibody, α-Ecad) would not phenocopy loss of *CDH1*, wherein both E-cadherin extracellular contacts and intracellular scaffolding and signaling would be lost. Indeed, acute E-cadherin contact disruption and genetic *CDH1* loss produce distinct cellular states, in particular related to EMT signatures, supporting that *CDH1* loss in ILC is a mechanistically distinct event in breast cancer development and progression. The concept that *CDH1* suppression vs E-cadherin inhibition are mechanistically distinct in mammary epithelial cells was previously observed by the Weinberg Lab [21], reporting that *CDH1* loss (via shRNA) was sufficient to induce EMT whereas a dominant-negative E-cadherin that disrupts adherens junctions was not. Importantly, this prior study was performed in SV40 Large T antigen + TERT immortalized HMLE cells and Ras-transformed derivative HMLER cells, wherein *CDH1* shRNA led to activation of β-catenin which drove EMT. Conversely, in ILC, *CDH1* loss is associated with loss of β-catenin and loss of canonical β-catenin/Wnt signaling [28,70]. Our use of finite lifespan HMEC with only *CCND1* overexpression, rather than immortalized/transformed HMLE and HMLER, may highlight that early loss of *CDH1* prior to the acquisition of extensive oncogenic changes is a critical feature of ILC oncogenesis, e.g., by inducing lineage remodeling that may alter the impact of subsequent genetic changes.

These observations highlight that lineage remodeling upon *CDH1* loss, and subsequent ILC oncogenesis, may yet reflect one or more related scenarios: 1) *CDH1* loss stabilizes or permits expansion of a luminal progenitor-like state; 2) *CDH1* loss directly instructs a luminal progenitor-like state in mammary epithelium; 3) *CDH1* loss selects for cells compatible with luminal (i.e., ER-positive)-associated programs. Scenarios 1 and 2 may work in concert; our findings support that direct remodeling and expansion both occur to create a population of cells primed to acquire additional transforming hits. Scenario 3 is likely associated with the insufficiency of *CDH1* loss alone to drive transformation [30] and the distinct repertoire of tumor mutations associated with ILC [36,71]. In that context, our use of *CCND1*-overexpressing HMEC (in addition to serving an important technical role in facilitating passage) is likely important in cooperating with *CDH1* loss to drive early ILC transformation phenotypes. *CDH1* loss may cooperate with ILC-enriched oncogenic hits (*FOXA1*, *PTEN*, *TBX3, ARID1A*, *AKT1, CCND1*) and antagonize ILC-depleted hits (*GATA3*, *MAP3K1*, *MAP2K4*). Similar findings in lung cancer models support that cell lineage defines tolerance for specific oncogenic hits; lung alveolar cells are transformed by EGFR but killed by Myc activation, with the converse in basal/neuroendocrine cells [72]. Future work will address how *CDH1* loss creates this primed state versus leverages specific states (e.g., *CCND1* gain) to drive ILC oncogenesis.

Defining the transcription or epigenomic factors that mediate changes in gene expression and lineage remodeling upon *CDH1* loss is a key next step in understanding the role of *CDH1*/E-cadherin as a tumor suppressor in ILC oncogenesis. One such factor identified as a candidate in our studies is inhibitor of DNA binding 2 (ID2). ID2 regulates commitment to luminal lineage [73], and has been shown to be transcriptionally induced upon E-cadherin loss whereupon it drives anoikis resistance in multiple models of ILC [6]; nuclear roles of ID2 are less well understood [6]. ID2 indirectly binds DNA via basic helix-loop-helix (bHLH) and ETS family transcription factors [74], which were among gained peaks in ATAC-seq upon *CDH1* loss (TFAP2C, ELK4, GABPA) [74–76]. Conversely, *CDH1* loss reduced accessibility at p63 motifs, consistent with the role of p63 as a basal transcription factor [77], yet whether p63 is directly or indirectly regulated by *CDH1* loss is unknown. Taken together, changes in chromatin accessibility and associated gene expression point toward potential factors that mediate luminal reprogramming upon *CDH1* loss, representing an important area of ongoing investigation.

We defined the putative lineage progenitor phenotype in *CDH1*-loss HMEC through upregulation and cell surface presentation of key markers (e.g. EpCAM^hi^ / CD49f^hi^ / CD10^hi^ status), and functional assays including the progenitor-derived colony forming assay, though historically, progenitor populations have been inconsistently defined. However, recent advances, such as single cell RNA-seq of human mammary cells compiled into the human breast cell atlas, have identified a broad diversity of cell types within the luminal and basal compartments within and across individuals [78]. Several luminal progenitor populations based on unbiased clustering have been defined by cytokeratin composition and expression of specific transcription factors. One such cluster (LASP2) was defined by increased expression of *KRT16* and *KRT6B*, which we observed in our *CDH1* loss models. The LASP2 cluster was also associated with reduced lineage fidelity, mirroring our observations. This KRT16 + *CDH1* loss-induced progenitor population could be distinctly susceptible to supporting progression toward ILC. Defining the subsequent susceptibility, and how *CDH1* loss interacts with subsequent genetic hits in the path to transformation, is important to understanding ILC oncogenesis. The HMEC models we used represent one stage of a series that have been moved through stepwise genetic progression from primary cells to transformed tumorigenic cells [33], and as such, make a strong platform to apply additional genetic hits to continue beyond early hyperplasia with *CDH1* loss, to overt ILC.

A key consideration for our experimental models of genetic *CDH1* loss are related to incomplete E-cadherin loss with siCDH1, and heterogenous E-cadherin loss in our Cas9 *CDH1*-KO models, both of which likely lead to mixed populations of E-cadherin-positive, -low, and -negative cells in our *CDH1*-loss studies. Directly confirming E-cadherin-negative status in post-*CDH1*-KO settings, i.e., in the induced EpCAM-high and/or CD10-high populations, would provide a stronger link between *CDH1*-loss and the identified phenotypes. However, technical limitations with flow cytometry assays (E-cadherin required fixed cells; lineage markers required fresh cells) precluded such direct measurements in our experiments. Similarly, the contributions of lineage remodeling versus selection, in particular in serial passage and longer-term assays, would be needed to define whether *CDH1* loss provides a selective advantage to cells. Importantly, our use of multiple HMEC models with differing baseline lineage populations and a series of orthogonal studies (S5 File) underpins support for the role of *CDH1* loss in mammary cell lineage and ILC oncogenesis.

*CDH1* loss induces luminal progenitor-like reprogramming in luminal and basal mammary cells, creating a cellular context that may be permissive to ILC oncogenesis. By identifying potential mechanistic drivers and proliferative processes associated with this reprogramming, our findings highlight new opportunities for identifying, profiling, and targeting early lobular lesions to assess risk and prevent progression to ILC. This work establishes a foundation for future investigations into the etiology of ILC, including mechanisms underlying its distinct patterns of invasion and metastasis.

## Materials and methods

### Ethics statement

IRB approvals were obtained from participating institutions (University of Minnesota Study # 1406E51485, July 05, 2014; Hennepin County Medical Center Study # 14–3842, July 9, 2014).

### Cell line culture and conditions

Female post-stasis human mammary epithelial cells (provided by Dr. Martha Stampfer, Lawrence Berkeley National Lab; https://hmec.lbl.gov) were maintained in M87A medium, which consists of a 1:1 mix of supplemented MEBM:DMEM/F12. MEBM (Lonza, cat#cc-3151) is supplemented with 5.0μg/mL Insulin (Thermo Fisher, cat# 41400–045), 70.0μg/mL bovine pituitary extract (Thermo Fisher, CAT # 13028014), 0.5μg/mL hydrocortisone (Sigma-Aldrich, Cat# H4001-1G), 5.0ng/mL EGF (Fisher Scientific, Cat # 236EG200), 5.0μg/mL transferrin (Invitrogen, cat# 11108–016), 10^-5^M Isoproterenol (IP) (Sigma, CAT# I5627), and 2.0mM glutamine (Sigma, Cat# 59202C). DMEM/F12 (Corning, CAT # 10–092-CV) is supplemented with 10μg/mL Insulin, 0.1μg/mL hydrocortisone, 2.5mL BSA (Sigma, CAT# A4161), 0.005μg/mL EGF, 2mM glutamine, 10μg/mL Cholera Toxin (Sigma, CAT #C8052), 1μM Oxytocin (Sigma, CAT# O6379-1MG), and 5% Albumax (Invitrogen, CAT # 11020–021).

HMEC lines were derived from patient/specimen numbers 184, 122, 153, and 240 [79]. Specific sublines are identified by their specimen number and Left/Right breast as needed, batch indicator if relevant, a dash (-) followed by the agent/gene used to induce post-stasis growth (-D1 for *CCND1*-overexpression), and any additional processing indicators. HMEC sublines used herein were specifically: 184D-D1L (batch D, enriched for luminal phenotype after -D1 expression); 122L-D1 (left breast); 153L-D1 (left breast); 240LB-D1 (left breast, batch B).

Cells were incubated at 37°C in 5% CO_2_. All lines were confirmed Mycoplasma negative and authenticated by STR profiling annually; validated cultures were maintained for no more than 3 months before thawing new cultures.

### E-cadherin antibody inhibition

E-cadherin inhibition was performed using HECD-1 antibody (Thermo Fisher, CAT# 131700) according to manufacturer’s instructions. For RNA-seq, HMEC were plated in 48 well plates and left overnight. Cells were treated with HECD-1 or IgG (mouse) at 25ug/mL for 48 hours. Cells were washed twice with PBS and stored in -80°C until RNA extraction.

### siRNA knockdown protocol

siRNA was reverse transfected using RNAiMAX (Thermo Fisher) by manufacturer instructions. Constructs are siGENOME SMART pool siRNAs (GE Healthcare Dharmacon): nontargeting pool #2 (D-001206-14-05) and human *CDH1* pool (M-003877-02-0005).

### Inducible CRISPR/Cas9

CRISPR/Cas9 models were established using Cas9 lentiviral vectors and lentiviral vectors obtained from CU Anschutz Functional Genomics Facility. gRNA clones were from the Sanger-Sigma Human Whole Genome Arrayed lenti CRISPR Library: 1 non-targeting gRNA (NTG) control (Lenti CRISPR Universal Non-Target Control) +2 *CDH1*-targeting gRNA (g1 – Clone ID 236996584, and g2 – Clone ID 236987757; lentiCRISPRv2-puro). *CDH1*-targeting gRNAs target exon 3 (g1) and exon 2 (g2). These exons make up the propeptide/signal domain, where one of the most commonly recurring *CDH1* mutations in ILC can be found [80], and will cause N-terminal frameshift mutations that ablate E-cadherin protein production. Induction of Cas9 and gRNAs was conducted over a 2-week period with fresh doxycycline (1μg/mL) every three-four days. To preserve HMEC line heterogeneity, a mixed population workflow was followed (i.e., cells were not clonally selected after doxycycline induction).

### RNA isolation

RNA extractions were performed using the RNeasy mini kit (Qiagen, cat# 74034); mRNA was converted to cDNA on an Eppendorf Mastercycler Pro (Eppendorf, Hamburg, Germany) and using iSCRIPT cDNA synthesis kit (BioRad, CAT# 1708890).

### RNA-seq

Library preparation and sequencing was performed at the U. Colorado Comprehensive Cancer Center Genomics Core Facility. Libraries were generated with Illumina Stranded mRNAPrep, and sequenced on a NovaSEQ6000 with 2150 paired-end reads, targeting 40x10^6^ clusters/80x10^6^ reads/sample (final mean = 39,019,637 clusters/sample). Data were analyzed with the UCCC Biostatistics and Bioinformatics Shared Resource. Illumina adapters and the first 12 base pairs/read were trimmed using BBDuk (RRID:SCR_016969) and<50 bp reads post-trimming were discarded. Reads were aligned and quantified using STAR (2.6.0a, RRID:SCR_004463) against the Ensembl human transcriptome (hg38.p12 genome,release 96), then normalized to counts per million by edgeR package (RRID:SCR_012802). Differential expression was calculated using limma R package (RRID:SCR_010943) and voom() function. Overrepresentation analysis was performed with cluster Profiler R (RRID:SCR_016884) and Molecular Signatures Database (RRID:SCR_016863) gene sets.

### RNAseq: Quantification and statistical analysis

RNAseq was performed using biological replicates, n = 3. All cells for each cell line were plated in the same 48 well plate. Biological triplicates are defined as 3 separate wells within the same plate. Reads were aligned and quantified using STAR (2.6.0a, RRID:SCR_004463) against the Ensembl human transcriptome (hg38.p12 genome,release 96), then normalized to counts per million by edgeR package (RRID:SCR_012802). Differential expression was calculated using limma R package (RRID:SCR_010943) and voom() function. Overrepresentation analysis was performed with cluster Profiler R (RRID:SCR_016884) and Molecular Signatures Database (RRID:SCR_016863) gene sets. For antibody inhibition: differential gene expression was analyzed with gene list cutoff using the adjusted p value 0.05. DGE lists were identified following comparison of DGE between HECD-1 vs. CTRL and IgG vs. CTRL, the final list included DGE only from HECD-1. This indirect comparison was performed, rather than direct HECD-1 vs. IgG (negative control) due to a higher than expected immune response induced by the IgG (differential gene expression for IgG vs untreated control is shown in S1 File). For all RNA-seq datasets: enriched pathways were identified using Enrichr. Heatmaps were generated using Morpheus with marker selection- signal to noise (α-Ecad vs. si*CDH1* and *CDH1*-KO).

### Single cell RNA-seq

Following CRISPR editing, single cell suspensions were prepared according to the recommendations of the Illumina Single Cell 3’ RNA Prep, T20 kit (cat# 20135692). A detailed workflow of cell processing, mRNA capture, and library preparation can be accessed at https://www.fluentbio.com/resource-category/user-guides/. Samples were pooled and sequenced on a 375Gb lane of the NovaSeq XPlus 10B platform using 150 paired-end base pair reads. Samples were demultiplexed and transcript mapping was performed using PIPseeker software, available at https://www.fluentbio.com/products/pipseeker-software-for-data-analysis/, yielding 1.1 billion barcoded reads between the two samples. Data was processed and analyzed using the Seurat R package [81]. High quality cells were filtered based on transcript count, gene count, mitochondrial content, and genes/UMI metrics. Prior to downstream analysis gene expression data was normalized and scaled. The top 3,000 variable genes were identified and a PCA and UMAP dimensionality reduction were performed. Cell clusters were defined using nearest-neighbor graph analysis and Seurat clustering algorithms. Cells were annotated as luminal or basal using established lineage marker gene signatures and module scoring. Independent reclustering of luminal and basal cell populations were performed. More detailed methods can be found in S1 Methods.

### ATAC-seq

For ATAC-seq, cells (HMEC 240D1 and 153D1) were plated in 6 well plates and treated with siRNA (siNT or si*CDH1*) for 72 hours. Cells were harvested, counted, and stored at -80C following Active Motif ATAC-seq cell preparation protocol. Cells were thawed, counted, *Drosophila* Spike-In control (Active Motif #53154) was added, and we proceeded to follow Active Motif’s ATAC-seq protocol (#53150). Transposed DNA was indexed using Illumina’s Nextera adapters within Active Motif’s ATAC-seq kit (#53150). Samples were checked for quality and sequenced by Novogene. Samples were sequenced with 20M PE reads on 10B NovaSeqXPlus.

### ATAC-seq data analysis

Sequence quality was assessed using FastQC (v0.11.9) and MultiQC (v1.14). Reads were trimmed for Nextera adapters and low-quality bases using BBDuk (v39.01), followed by contamination screening with FastQ Screen (v0.15.3). Alignments to the GRCh38 human reference genome were performed using Bowtie2 (v2.5.0). Post-alignment, mitochondrial reads were removed, and fragments were filtered to retain high-confidence, properly paired alignments (MAPQ > 30) using Samtools (v1.16.1). Artifact-prone regions were excluded using the GRCh38 unified blacklist and Bedtools (v2.29.1). For comparative analysis, libraries were normalized by randomly sub-sampling reads to a uniform depth across samples. Normalized coverage tracks (BigWig) were generated with deepTools bamCoverage (v3.5.2). Peaks were called using MACS3 (v3.0.3), identifying high-confidence narrow peaks with a significance threshold of q < 0.05. Data were visualized using the Integrative Genomics Viewer (IGV, v2.18.4).

Differential accessibility analysis was performed using DiffBind (v3.20), retaining only reproducible peaks present in at least two samples. Sub-sampled reads within these consensus regions were quantified via summarizeOverlaps, normalized using Relative Log Expression (RLE) scaling based on full library sizes, and modeled with DESeq2 to identify significant differentially accessible regions (FDR < 0.05, minimum 1.25-fold change) between siCDH1 and siNT conditions. Genomic distribution of peaks (maintained, gained and lost) was determined using R/Bioconductor package ChIPseeker [82].

BETA-minus was used to identify nearby genes within 30kB of a peak [83]. BETA-basic was used to perform an integrated analysis of ATAC-seq and RNAseq from HMEC 153D1 and HMEC 240D1 cell models (Web-based platform Cistrome). Heatmaps were created using “compute matrix” and “plotHeatmap” tools from web-based platform Galaxy (see S1 Methods for more details).

Motif enrichment analysis was performed using i-cisTarget (https://gbiomed.kuleuven.be/apps/lcb/i-cisTarget/index.php) [52]. First, we did individual analyses using the BED files of gained peaks and lost peaks for both cell lines. Then we used i-cisTarget comparative analysis to find the shared motifs within the gained peaks across both cell lines and within the lost peaks across both cell lines.

### LCIS sample selection, pathology review, RNA isolation, and bioinformatics

#### Sample selection and pathology review.

A total of 198 cases that contained “LCIS” in the diagnostic report were identified between the years 2010 – 2014. Pathology slide review identified 24 cases of cLCIS and 7 cases of potential pLCIS which contained sufficient LCIS tissue for analysis (>20 mm2). LCIS lesions were adequately separate from any concomitant invasive cancer to ensure accurate enrichment of only LCIS from FFPE sections. Cases were independently reviewed by 3 board-certified surgical pathologists (MK, SC, ACN) for diagnostic classification as classic or pleomorphic LCIS; only cases with independent, 3-sided consensus agreement were classified as pLCIS and all others were classified as classic. One pathologist evaluated the cohort for 6 additional qualitative morphologic characteristics: nuclear contour, nuclear size, chromatin quality, extent of necrosis, presence of mitotic figures, and inflammatory infiltrate (scored on a 1–3 scale). For necrosis scoring: absent = 1; rare, small foci < 5 cell diameters = 2; and prominent central necrosis = 3. For mitoses scoring: absent to rare mitotic figures across all involved lobules = 1; the presence of at least 1 mitotic figure in two or more involved lobular units = 2; and the presence of multiple lobular units with >1 mitotic figure each = 3. Neoplastic cell percentage was minimally 50% in areas macro-enriched for analysis; cases were further categorized into three tiers of neoplastic cellularity: 50–60%, 60–80%, and >80%. 7 control cases from mammoplasty reduction were identified to obtain benign breast tissue.

#### FFPE RNA isolation.

Four consecutive 7-µm thick section of FFPE breast tissue from each FFPE block were cut using a fresh microtome blade and mounted on new RNA-free slides. Equipment sterilization with RNase AWAY (Molecular BioProducts Inc, San Diego, CA, USA) was completed between blocks. Following mounting, sections were immediately transferred to a -80C freezer and stored no longer than 2 weeks. Xylene deparaffinization and staining were completed as described in the Arcturus Paradise PLUS reagent system user guide (Life Technologies, Carlsbad, CA, USA). Areas containing cLCIS, pLCIS and representative normal breast tissue were identified and macro-dissected and stored at -80C. Total RNA was then isolated and purified using the Qiagen’s RNeasy Mini Kit (Hilden, Germany). RNA concentration and purity were measured with a Nano-Drop 2000 spectrophotometer (Thermo FisherScientiﬁc, Waltham, MA, USA).

#### RNA quality control and NGS sequencing.

RNA-seq was performed using the TruSeq RNA Access Library Prep Kit from Illumina (San Diego, CA, USA). RNA sample quantity and quality was assessed using a 2100 Bioanalyzer (Agilent, Santa Clara, CA, USA) following the DV200 recommendations of the TruSeq RNA Access Kit. Sequencing was completed on an Illumina HiSeq 2500 with 2 x 100 bp reads, producing an average of approximately 27 M reads/sample. Successful RNAseq data were obtained from 18 cLCIS, 4 pLCIS, and 7 control samples.

#### Bioinformatic and statistical analysis.

Reads were aligned and quantified using STAR (2.6.0a, RRID:SCR_004463) against the Ensembl human transcriptome (hg38.p12 genome,release 96), then normalized to counts per million by edgeR package (RRID:SCR_012802). Differential expression was calculated using limma R package (RRID:SCR_010943) and voom() function. Heatmap was created using Morpheus (https://software.broadinstitute.org/morpheus) and clusters were defined using K means. Gene set enrichment analysis and overrepresentation analyses were performed using BBSR tools at CU Anschutz (https://bioinformatics.cuanschutz.edu/).

### Flow cytometry

For extracellular flow cytometry, cells were harvested with 0.05% trypsin/EDTA. Cells were counted, washed with PBS, and resuspended in staining solution (PBS, 1% BSA, 1% EDTA). Cells were plated into Falcon 96-well round bottom plates (Corning # 353077) at 400,000 cells/well. EpCAM-BV421 (Biolegend #324220, clone 9C4, mouse IgG2b, 1:200), CD10-APC/Fire 750 (Biolegend #312230, clone HI10a, mouse IgG1, 1:200), and CD49f-PE (Biolegend #313612, clone GoH3, Rat IgG2a, 1:400) were added to cells in staining solution for 15 minutes on ice. Cells were centrifuged for 3 min at 1400rpm. Staining solution was aspirated and cells were washed with PBS for 10 min on ice. Cells were centrifuged for 4 min at 1400rpm. PBS was aspirated and cells were resuspended in flow solution (PBS, 2% BSA, 0.2% EDTA). Cells were then analyzed in a Novocyte Penteon Flow Cytometer. SpectraComp beads (Bio Slingshot # SSB-05-B), unstained cells, and FMOs were used as controls.

For flow sorting, cells were prepared following the same protocol as the extracellular flow cytometry analysis. Cells were only stained with EpCAM-BV421 (Biolegend #324220, clone 9C4, mouse IgG2b, 1:200). Cells were sorted using the Sony MA900; sorting gates were set using unstained controls. For the lineage-sorting experiment shown in Fig 6, results are representative of two independent experiments for the full timecourse (2 weeks) and further replicates of partial timecourse studies (24 hrs and 4 weeks). 24hr sort-efficiency checks (intended as sort validation only) represent technical duplicates, 2-week samples represent a biological triplicate and single replicates (due to limited material) from independent experiments, and 4-week samples represent biological triplicate pooled (due to limited material at extended time).

For intracellular flow cytometry, cells were harvested with 0.05% trypsin/EDTA. Cells were counted, washed with PBS, and fixed in 4%PFA (100uL/1x10^6^ cells) and incubated at room temperature for 8 minutes. Cells were washed with PBS by centrifugation. Cells were resuspended in 1% Triton-X (100uL/1x10^6^ cells) and incubated at room temperature for 10 minutes. Cells were then washed with PBS by centrifugation and resuspended in staining solution. E-cadherin- AF647 (Cell Signaling #9835, clone 24E10, Rabbit IgG, 1:200) was added to the cells and the staining flow protocol above was followed.

### Population doubling assay

Total population doubling over a finite lifespan for each culture was calculated using the formula: population doubling = log_2_(N_final_/N_initial_), where N_initial_ is the number of cells seeded in a dish at each passage and N_final_ is the number of cells recovered from the dish.

### Primary sphere culture and fibroblast co-culture as*say*

Primary mammary epithelial cells were cultured in 2D or as spheres using ultra-low attachment conditions. Briefly, 1 × 10^5^ cells per well were plated in 6-well ultra-low attachment plates and maintained in M87A medium for 7 days to allow sphere formation. Spheres were collected into 2-mL microcentrifuge tubes and pelleted by centrifugation at 900 rpm for 5 minutes at room temperature. The supernatant was aspirated, and sphere pellets were resuspended in 0.5–1 mL of 0.05% trypsin/0.025% EDTA, depending on pellet size, and incubated at 37°C for 10 minutes for enzymatic dissociation. Mechanical dissociation was performed by gentle pipetting (5–10 times) using a 1000-μL pipette. The resulting cell suspension was transferred to 15-mL conical tubes, and trypsin was neutralized with 5–10 mL of calcium- and magnesium-free PBS (CMF-PBS) supplemented with 10% fetal bovine serum (FBS). Cells were passed through a 40-μm nylon mesh filter, pelleted by centrifugation (900 rpm, 5 minutes, room temperature), and resuspended for viable cell counting using a hemocytometer.

For co-culture experiments, NIH-3T3 murine fibroblasts were irradiated with 30 Gy one day prior to use. Irradiated fibroblasts were plated in 24-well tissue culture plates at a density of 2 × 10⁴ cells/cm^2^ in DMEM supplemented with 10% FBS. On the day of sphere dissociation, single-cell suspensions derived from primary spheres were seeded onto the NIH-3T3 feeder layer at a density of 100 cells/mL for 2D culture and 1,000 cells/mL for 3D culture. Co-cultures were maintained at 37°C in a humidified incubator. Medium was replenished every 48 hours by performing a half-medium change, in which approximately half of the conditioned medium was removed and replaced with fresh M87A medium. After 10 days of co-culture, colonies were gently washed with 0.5–1mL CMF-PBS and fixed with 250–500μL ice-cold methanol for 5 minutes, taking care to avoid direct disruption of colonies. Methanol was aspirated, and colonies were stained with Wright dye (150–200μL per well) for 1 minute at room temperature for colony phenotype analysis. Luminal progenitors form rounded, tightly packed colonies, while basal progenitors form irregularly shaped colonies with more scattered cells and mixed phenotype progenitors show both features [67].

Data shown (Fig 5) are representative of three independent experiments. Images shown in 5D/F are directly from the experiment quantified in 5E/G. Colony phenotype scoring was not blinded, but was conducted independently by two investigators to affirm phenotype calls.

### Anoikis resistance assay

*CDH1*-KO was induced in HMEC 184D1 (NTG used as control). Cells were harvested, resuspended, and transferred to ultra-low attachment plates; seeding was normalized across conditions using trypan blue-negative cell counts (i.e., viable cell counts). After 3 days, cells were harvested, washed, and resuspended in 1xPBS with fixable live/dead dye according to the protocol (Thermo Fisher # L23105). Then cells were fixed and prepared for flow cytometry as described above. Cells were also stained with E-cadherin-AF647 (Cell Signaling #9835, clone 24E10, Rabbit IgG, 1:200). Unstained samples (mix of NTG and *CDH1*-KO cells) were used to set the gate for E-cadherin Hi and E-cadherin Lo populations. Anoikis was quantified based on live/dead fluorescence in E-cadherin Hi and E-cadherin Lo populations, separately; additional treatments to induce cell death were not included in these assays.

## Supporting information

S1 Fig E-cadherin suppression within each model. A) E-cadherin functional assay compares binding between HMEC 184D1, 153D1, 240D1 cells treated with HECD-1 vs. IgG and untreated. Plates were coated with E-cadherin at 3 ug/mL. Adhesion was quantified using GFP fluorescence. Bars represent average and standard deviation of percent adhesion (n = 3). B) Western blot of E-cadherin abundance (clone: 24E10) in HMEC 184D1, 153D1, 240D1 cells treated with si*CDH1* or siNT for 72 hours. %E-cadherin normalized to GAPDH as a loading control (ImageJ). C) Representative flow cytometry quantification of E-cadherin in HMEC after siCDH1, compared to E-cadherin negative ILC cell line MDA MB 134VI. D) Representative flow plots show *CDH1* KO efficiency. The first plot is HMEC 184D1 unstained negative control and was used to set the gate for E-cadherin negative (left of the gate) and E-cadherin positive (right of the gate). The next three plots were stained with E-cadherin Ab (clone: 24E10) conjugated to Alexa Fluor 647 (AF647). The x-axis is the logarithmic fluorescent intensity of AF647-E-cadherin. The percent of E-cadherin negative cells is in the top left of each plot and the percent of E-cadherin positive cells is in the top right of each plot. E) Quantification of percent E-cadherin negative cells across three biological replicates in both CDH1 KO g1 and g2 models and the control NTG model. F) Representative flow plots of size (FSC-H) vs. E-cadherin fluorescent intensity of individual cells in NTG, *CDH1*-KO g1 and *CDH1*-KO g2. G) Quantification of E. Violin plot showing the distribution of E-cadherin fluorescent intensity per cell in NTG, *CDH1*-KO g1 and *CDH1*-KO g2. **** adj. p < 0.0001 (one way-ANOVA).
(PDF)

S2 Fig Fast gene set enrichment analysis. (FGSEA) normalized enrichment scores (NES) of Hallmark pathways (q value < 0.05; *EMT in 240D1 q val = 0.06).
(PDF)

S3 Fig Differential gene expression within the Hypoxia signature. Fold change of gene expression (p < 0.05) between E-cadherin inhibition and genetic CDH1 loss within each cell line (HMEC 122D1, 153D1, 184D1, 240D1) in the Hallmark pathway hypoxia signature.
(PDF)

S4 Fig Luminal/Basal composition of HMEC models. A) Distribution of luminal and basal cells within each of the 4 HMEC models using EpCAM and CD49f. B) Relative quantification of luminal/basal composition within HMEC *CDH1* KO models using counts per million (CPM) ratios of the luminal to basal genes highlighted in Fig 1D.
(PDF)

S5 Fig Tabula Muris gene signatures comparing luminal vs. basal in sc-RNAseq. Overrepresentation analysis of only upregulated genes (luminal *CDH1*-KO vs. NTG; basal *CDH1*-KO vs. NTG) of mammary signatures in Tabula Muris cell-type pathways. (adj. p < 1x10^-10^, -0.3 > FC > 0.3).
(PDF)

S6 Fig *CDH1* KO induces reprogramming in luminal and basal cells toward a luminal progenitor-like state. A) Differentially expressed genes of *CDH1* KO vs. NTG cells within the luminal G1 and basal G1 populations relating to progenitor phenotype and cholesterol synthesis; tables on the right show the number of cells included in the analysis and the number of DGE for *CDH1* KO vs. NTG within Luminal G1 and Basal G1 cells, respectively (adj. p < 1x10^-10^, -0.3 > FC > 0.3). B) Feature plots of two of the top 10 induced genes in CDH1 KO luminal G1 and basal G1cells: KRT16 and ID2 mapped onto original sc-RNAseq feature plot including all NTG vs. CDH1 KO cells (KRT16: avg. logFC = 1.79; pct.1 = 0.70; adj. p val = 0; ID2: avg. logFC = 2.03; pct.1 = 0.38; adj. p val = 0). C) FGSEA of Cholesterol Homeostasis Hallmark pathway in Luminal G1 and Basal G1 cells (luminal G1 NES = 2.48, adj. p = 0.0027; basal G1 NES = 1.71, adj. p = 0.0706).
(PDF)

S7 Fig *CDH1*-KO chromatin landscape and associated gene expression changes. A) Pie charts show the distribution of reproducible and differentially expressed peaks found in promoters, introns, distal intergenic regions, exons, 3’UTR, 5’UTR, and other. B) Scatter plot of 240D1 ATACseq fold changes of differential peaks vs. fold changes in corresponding gene expression from RNAseq. C) Violin plot shows fold changes of the differentially expressed genes from RNAseq (adj. p < 0.05) that are associated with gained or lost peaks within each cell line. The average fold change for genes associated with gained peaks in 153D1 was 0.55, whereas genes associated with lost peaks had an average fold change of -0.44. A similar trend was seen in the 240D1 (FC of genes associated with gained peaks = 0.46; FC of genes associated with lost peaks = -0.22). One-way ANOVA: **** (adj. p val < 0.0001).
(PDF)

S8 Fig Gating for flow analyses after flow sort. A-C) Fluorescence minus one (FMO) samples were used to set the gates for EpCAM and CD49f for all three post sort analyses including A) 24hrs post sort, B) 2 weeks post dox, and C) 4 weeks post dox. Gates for each day include Cells (SSC-H vs. FSC-H), Single Cells (FSC-A vs. FSC-H), EpCAM (Count vs. EpCAM-BV421), and CD49f (Count vs. CD49f-PE). The samples used to set the gates include a mixture of cells from each sorted population (NTG EpCAM Hi, NTG EpCAM Lo, *CDH1*-KO EpCAM Hi, and *CDH1*-KO EpCAM Lo). The left column shows the FMO without EpCAM antibody and the right column shows the FMO without the CD49f antibody. Antibody details can be found in methods.
(PDF)

S9 Fig *CDH1*-KO Time Course with *CDH1*-KO g1. A) Relative EpCAM levels (NTG/EpCAM low as baseline) in post-sorting populations over a complete timecourse; results shown are for a single timecourse experiment and representative of repeated experiments (see Fig 6). Points represent biological replicates. B) Time course western blots of E-cadherin with Vinculin loading control. C) Paired time course samples analyzed via qPCR for basal and luminal gene expression.
(PDF)

S1 File Differentially expressed genes in HMEC RNA-seq studies. Includes summary table for numbers of genes and statistical cutoffs, and gene tables for each model and E-cadherin targeting mode associated with Fig 1.
(XLSX)

S2 File Chromatin accessibility peaks from HMEC ATAC-seq. BED format tables for peaks identified in HMEC ATAC-seq, including those gained versus lost after *CDH1* siRNA.
(XLSX)

S3 File Integrated ATAC-seq and RNA-seq analyses. Data on ATAC-seq peak association with genes regulated by CDH1 suppression in RNA-seq.
(XLSX)

S4 File LCIS sample tables and data analysis. Clinicopathological features for LCIS and normal tissues associated with RNA-seq studies. Includes differentially expressed gene lists and pathway analyses.
(XLSX)

S5 File Model and data summary. Tables providing an overview of orthogonal approaches and models used throughout, linking figures and associated data to specific key conclusions.
(XLSX)

S1 Methods Additional methods information for sequencing-based studies and cell culture-based experiments with associated references, and Key Resources tables. (DOCX)
