# Supplementary material for: CDH1 loss remodels gene expression and lineage identity in human mammary epithelial cells
Source: PLoS Genet. 2026 Oct 27;22(10):e1012351. doi: 10.1371/journal.pgen.1012351 (PMC13722294; doi:10.1371/journal.pgen.1012351)
Supplement: Supplementary file 1 [file pgen.1012351.s001.pdf]

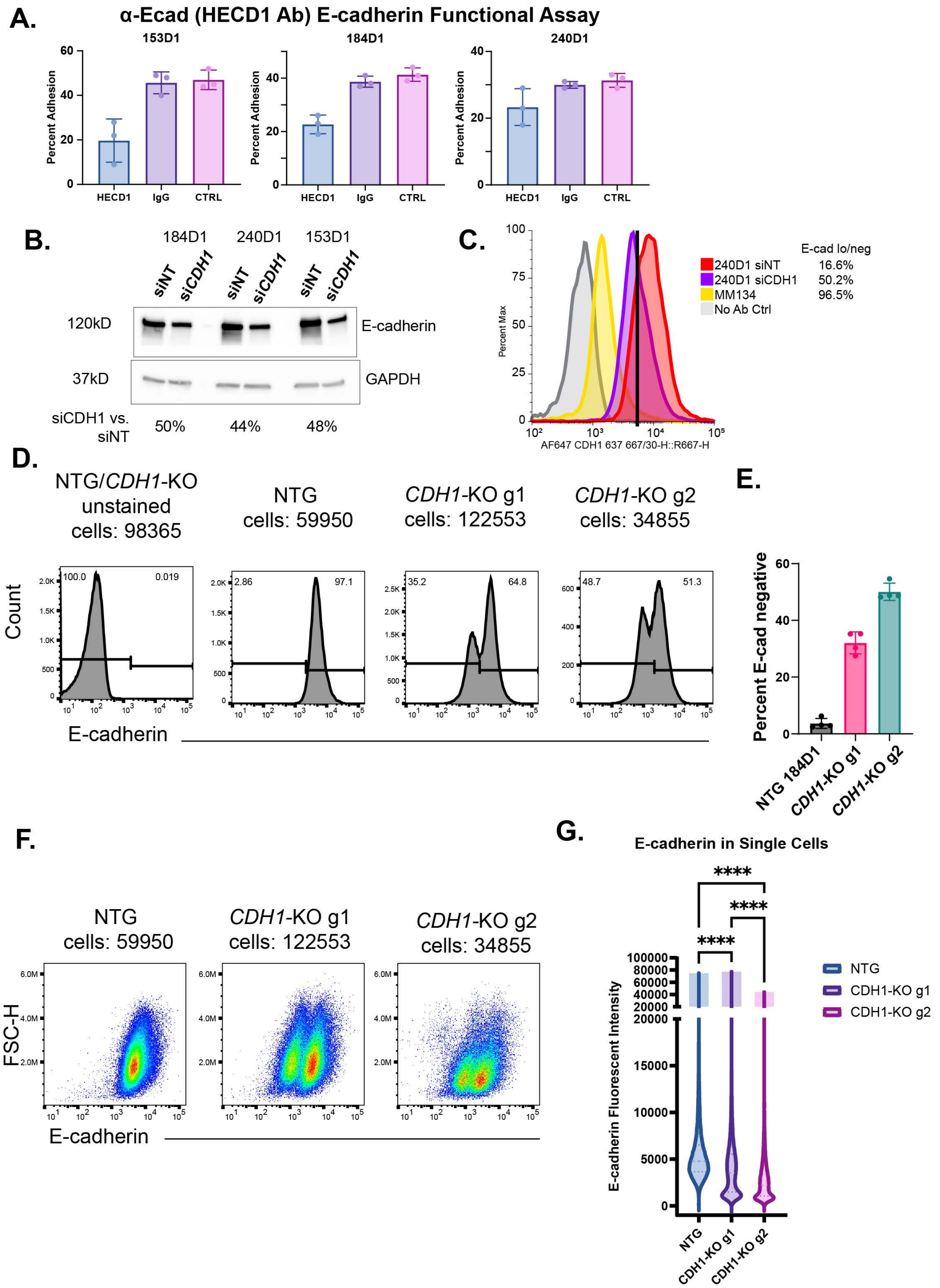

Supplemental Figure 1. E-cadherin suppression within each model. A) E-cadherin functional assay compares binding between HMEC 184D1, 153D1, 240D1 cells treated with HECD-1 vs. IgG and untreated. Plates were coated with E-cadherin at 3 ug/mL. Adhesion was quantified using GFP fluorescence. Bars represent average and standard deviation of percent adhesion (n=3). B) Western blot of E-cadherin abundance (clone: 24E10) in HMEC 184D1, 153D1, 240D1 cells treated with si*CDH1* or siNT for 72 hours. %E-cadherin normalized to GAPDH as a loading control (ImageJ). C) Representative flow cytometry quantification of E-cadherin in HMEC after siCDH1, compared to E-cadherin negative ILC cell line MDA MB 134VI. D) Representative flow plots show *CDH1* KO efficiency. The first plot is HMEC 184D1 unstained negative control and was used to set the gate for E-cadherin negative (left of the gate) and E-cadherin positive (right of the gate). The next three plots were stained with E-cadherin Ab (clone: 24E10) conjugated to Alexa Fluor 647 (AF647). The x-axis is the logarithmic fluorescent intensity of AF647-E-cadherin. The percent of E-cadherin negative cells is in the top left of each plot and the percent of E-cadherin positive cells is in the top right of each plot. E) Quantification of percent E-cadherin negative cells across three biological replicates in both CDH1 KO g1 and g2 models and the control NTG model. F) Representative flow plots of size (FSC-H) vs. E-cadherin fluorescent intensity of individual cells in NTG, *CDH1*-KO g1 and *CDH1*-KO g2. G) Quantification of E. Violin plot showing the distribution of E-cadherin fluorescent intensity per cell in NTG, *CDH1*-KO g1 and *CDH1*-KO g2. **** adj. $p < 0.0001$ (one way-ANOVA).
