# Supplementary material for: CDH1 loss remodels gene expression and lineage identity in human mammary epithelial cells
Source: PLoS Genet. 2026 Oct 27;22(10):e1012351. doi: 10.1371/journal.pgen.1012351 (PMC13722294; doi:10.1371/journal.pgen.1012351)
Supplement: Supplementary file 2 [file pgen.1012351.s002.pdf]

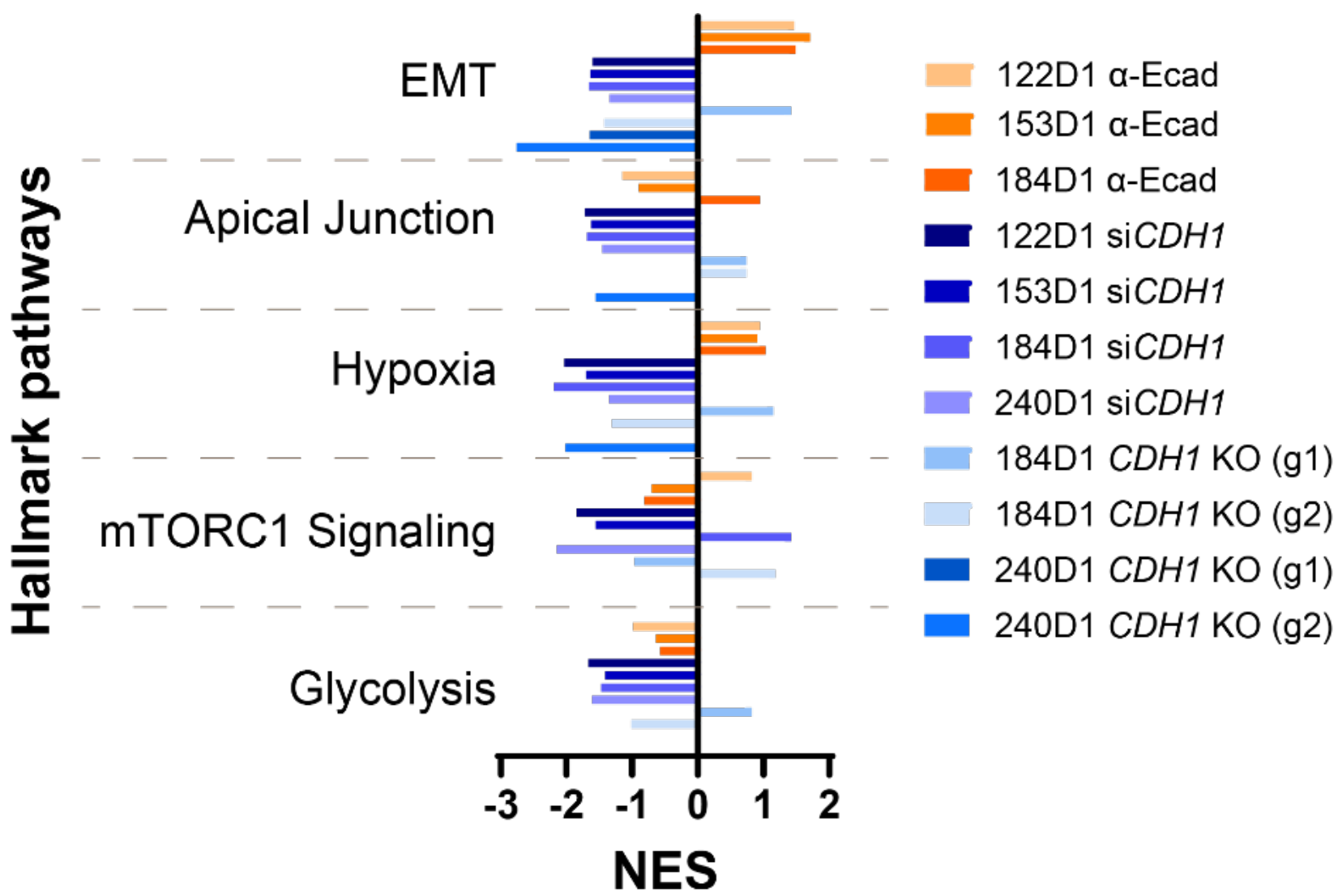

Supplemental Figure 2. Fast gene set enrichment analysis (FGSEA) normalized enrichment scores (NES) of Hallmark pathways (q value < 0.05; *EMT in 240D1 q val=0.06).
