# Supplementary material for: CDH1 loss remodels gene expression and lineage identity in human mammary epithelial cells
Source: PLoS Genet. 2026 Oct 27;22(10):e1012351. doi: 10.1371/journal.pgen.1012351 (PMC13722294; doi:10.1371/journal.pgen.1012351)
Supplement: Supplementary file 3 [file pgen.1012351.s003.pdf]

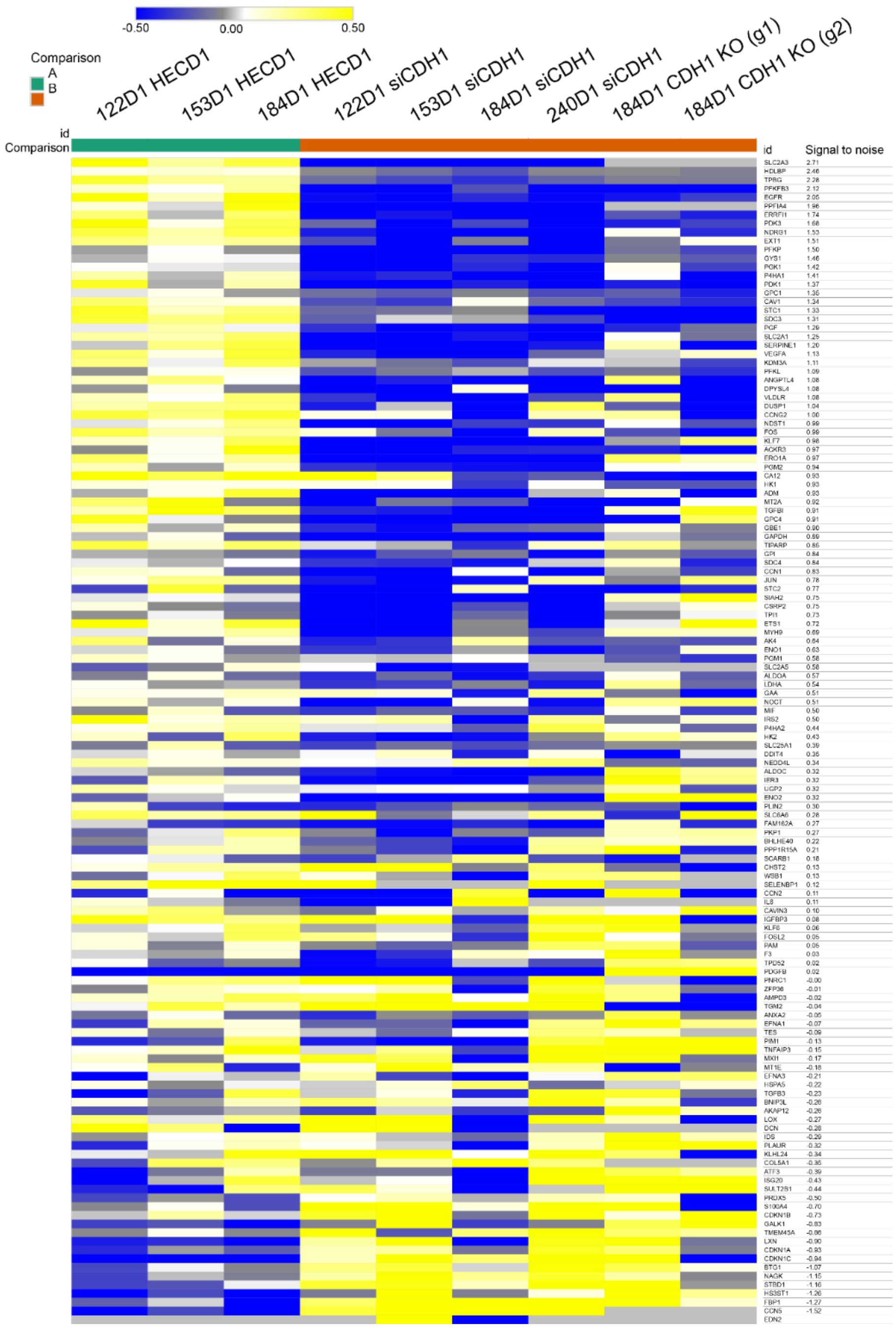

Supplemental Figure 3. Differential gene expression within the Hypoxia signature. Fold change of gene expression ($p<0.05$) between E-cadherin inhibition and genetic *CDH1* loss within each cell line (HMEC 122D1, 153D1, 184D1, 240D1) in the Hallmark pathway hypoxia signature.
