# Supplementary material for: CDH1 loss remodels gene expression and lineage identity in human mammary epithelial cells
Source: PLoS Genet. 2026 Oct 27;22(10):e1012351. doi: 10.1371/journal.pgen.1012351 (PMC13722294; doi:10.1371/journal.pgen.1012351)
Supplement: Supplementary file 4 [file pgen.1012351.s004.pdf]

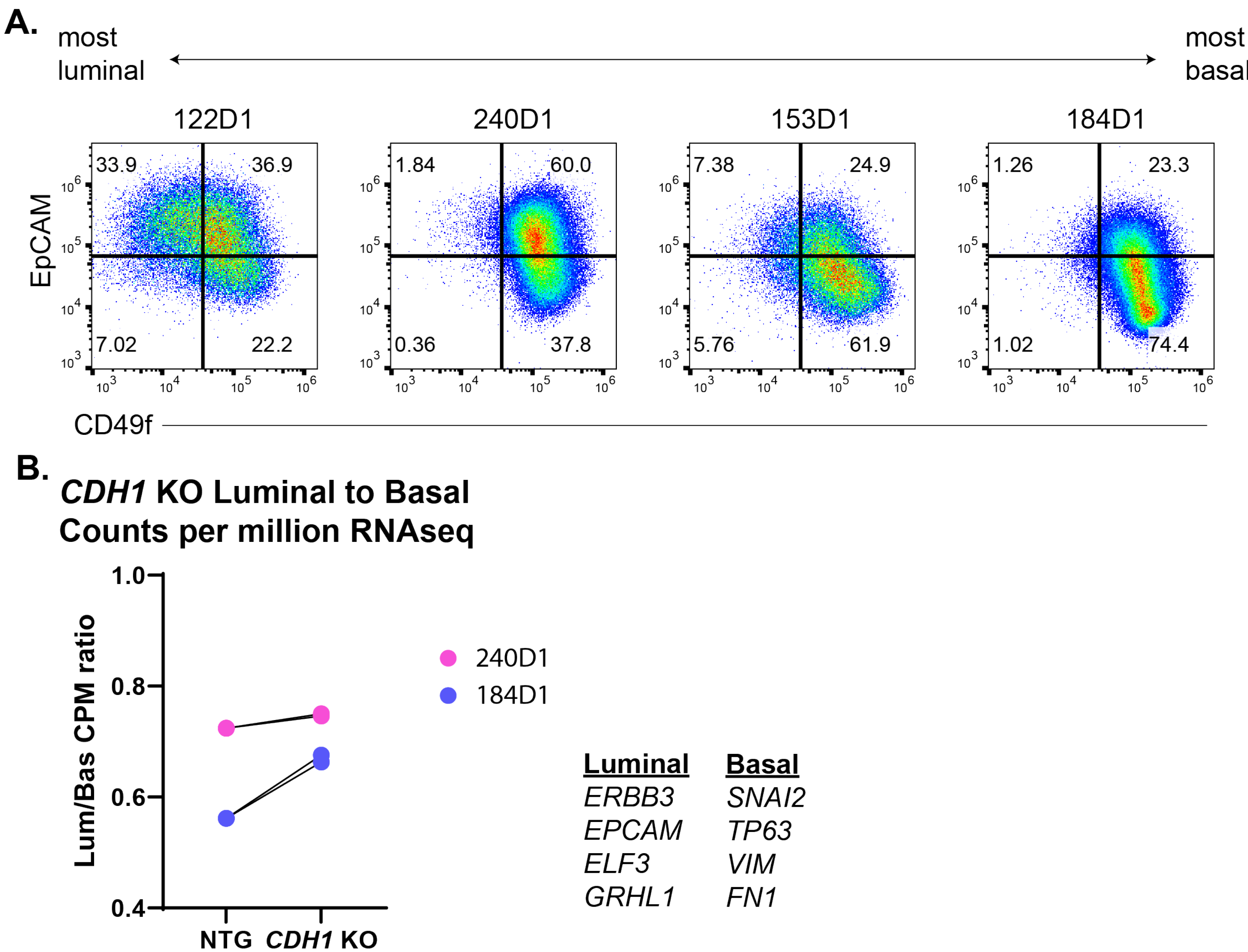

Supplemental Figure 4. Luminal/Basal composition of HMEC models. A) Distribution of luminal and basal cells within each of the 4 HMEC models using EpCAM and CD49f. B) Relative quantification of luminal/basal composition within HMEC *CDH1* KO models using counts per million (CPM) ratios of the luminal to basal genes highlighted in Figure 1D.
