# Supplementary material for: CDH1 loss remodels gene expression and lineage identity in human mammary epithelial cells
Source: PLoS Genet. 2026 Oct 27;22(10):e1012351. doi: 10.1371/journal.pgen.1012351 (PMC13722294; doi:10.1371/journal.pgen.1012351)
Supplement: Supplementary file 5 [file pgen.1012351.s005.pdf]

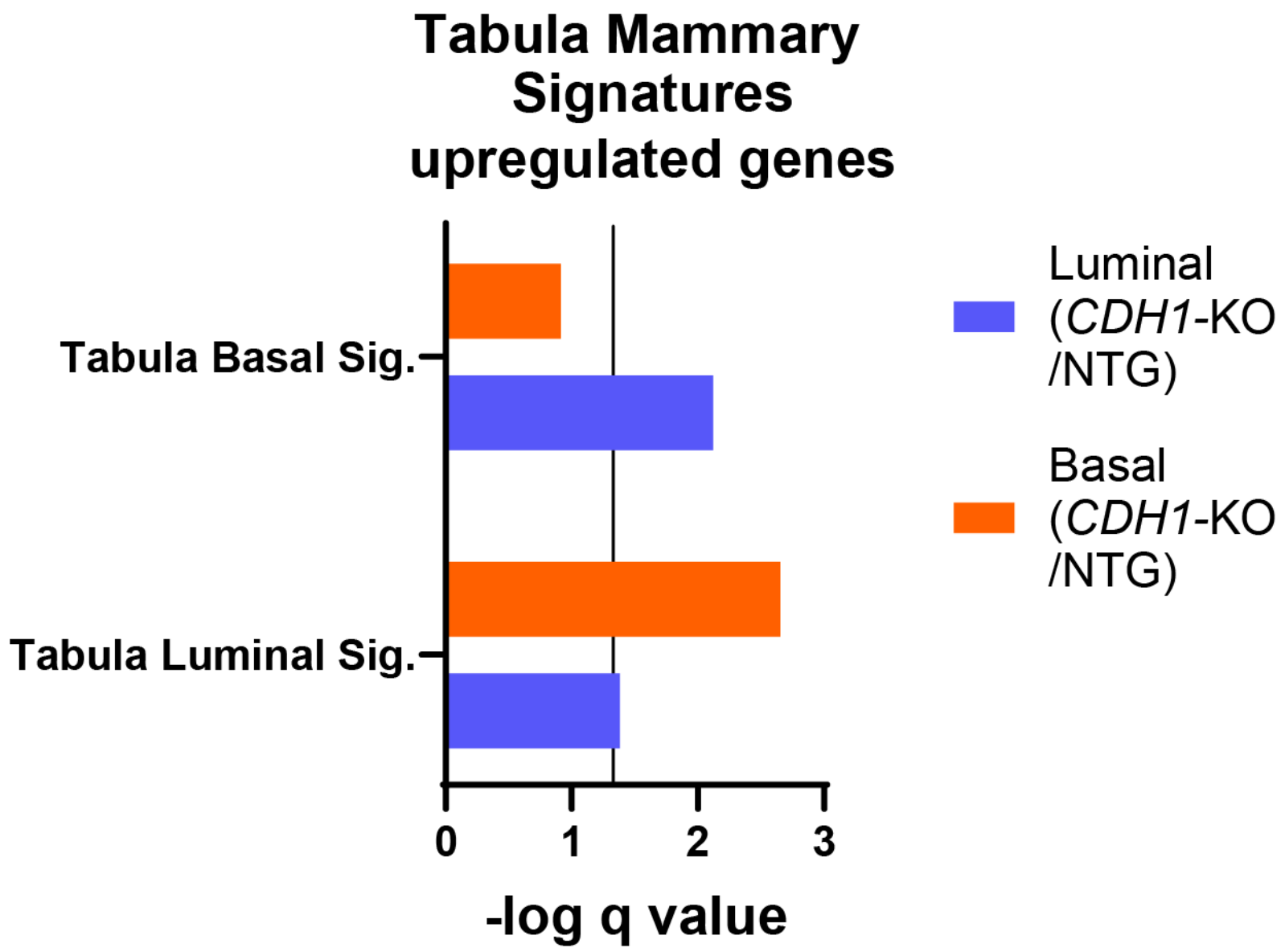

Supplemental Figure 5. Tabula Muris gene signatures comparing luminal vs. basal in sc-RNAseq. Overrepresentation analysis of only upregulated genes (luminal *CDH1*-KO vs. NTG; basal *CDH1*-KO vs. NTG) of mammary signatures in Tabula Muris cell-type pathways. (adj. p $<1x10^{-10}$, -0.3>FC>0.3).
