# Supplementary material for: CDH1 loss remodels gene expression and lineage identity in human mammary epithelial cells
Source: PLoS Genet. 2026 Oct 27;22(10):e1012351. doi: 10.1371/journal.pgen.1012351 (PMC13722294; doi:10.1371/journal.pgen.1012351)
Supplement: Supplementary file 6 [file pgen.1012351.s006.pdf]

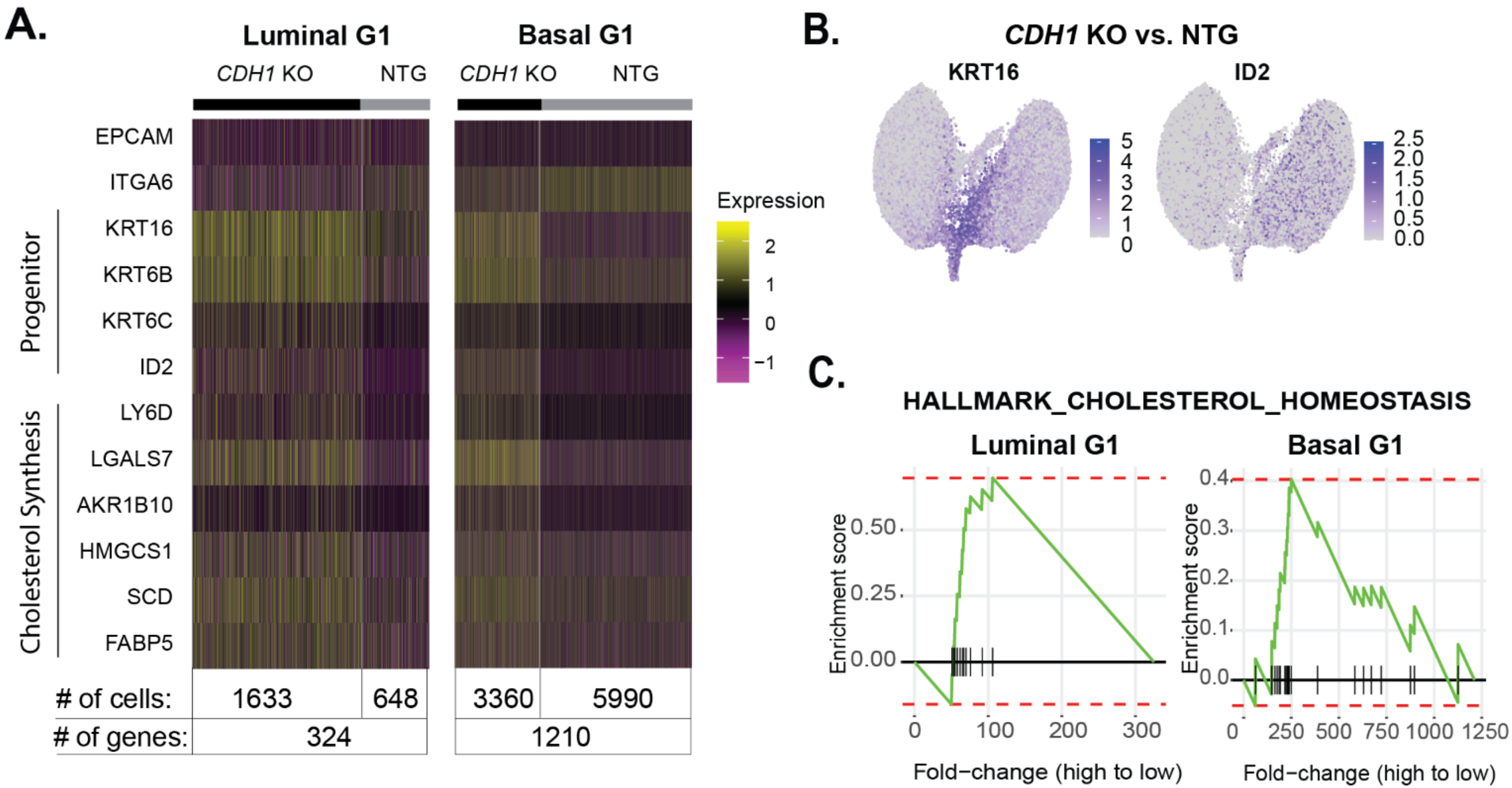

Supplemental Figure 6. ***CDH1*** **KO induces reprogramming in luminal and basal cells toward a luminal progenitor-like state.** A) Differentially expressed genes of *CDH1* KO vs. NTG cells within the luminal G1 and basal G1 populations relating to progenitor phenotype and cholesterol synthesis; tables on the right show the number of cells included in the analysis and the number of DGE for *CDH1* KO vs. NTG within Luminal G1 and Basal G1 cells, respectively (adj. p $<1\times10^{-10}$, -0.3>FC>0.3). B) Feature plots of two of the top 10 induced genes in CDH1 KO luminal G1 and basal G1cells: KRT16 and ID2 mapped onto original sc-RNAseq feature plot including all NTG vs. CDH1 KO cells (KRT16: avg. logFC= 1.79; pct.1=0.70; adj. p val = 0; ID2: avg. logFC= 2.03; pct.1=0.38; adj. p val = 0). C) FGSEA of Cholesterol Homeostasis Hallmark pathway in Luminal G1 and Basal G1 cells (luminal G1 NES= 2.48, adj. p=0.0027; basal G1 NES= 1.71, adj. p= 0.0706).
