# Supplementary material for: CDH1 loss remodels gene expression and lineage identity in human mammary epithelial cells
Source: PLoS Genet. 2026 Oct 27;22(10):e1012351. doi: 10.1371/journal.pgen.1012351 (PMC13722294; doi:10.1371/journal.pgen.1012351)
Supplement: Supplementary file 7 [file pgen.1012351.s007.pdf]

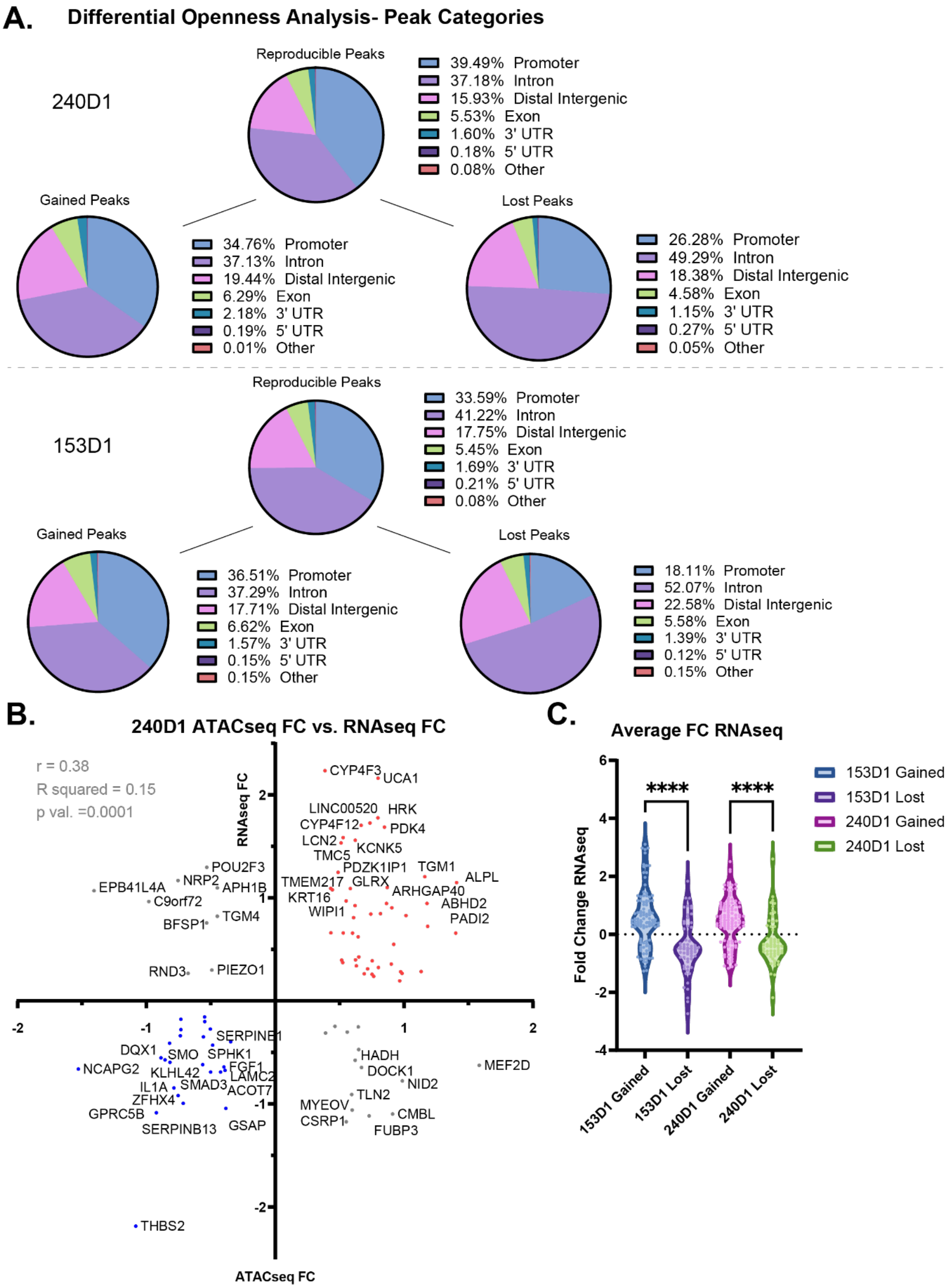

Supplemental Figure 7. *CDH1*-KO chromatin landscape and associated gene expression changes. A) Pie charts show the distribution of reproducible and differentially expressed peaks found in promoters, introns, distal intergenic regions, exons, 3’UTR, 5’UTR, and other. B) Scatter plot of 240D1 ATACseq fold changes of differential peaks vs. fold changes in corresponding gene expression from RNAseq. C) Violin plot shows fold changes of the differentially expressed genes from RNAseq (adj. $p < 0.05$) that are associated with gained or lost peaks within each cell line. The average fold change for genes associated with gained peaks in 153D1 was 0.55, whereas genes associated with lost peaks had an average fold change of -0.44. A similar trend was seen in the 240D1 (FC of genes associated with gained peaks = 0.46; FC of genes associated with lost peaks = -0.22). One-way ANOVA: **** (adj. p val < 0.0001).
