# Supplementary material for: CDH1 loss remodels gene expression and lineage identity in human mammary epithelial cells
Source: PLoS Genet. 2026 Oct 27;22(10):e1012351. doi: 10.1371/journal.pgen.1012351 (PMC13722294; doi:10.1371/journal.pgen.1012351)
Supplement: Supplementary file 8 [file pgen.1012351.s008.pdf]

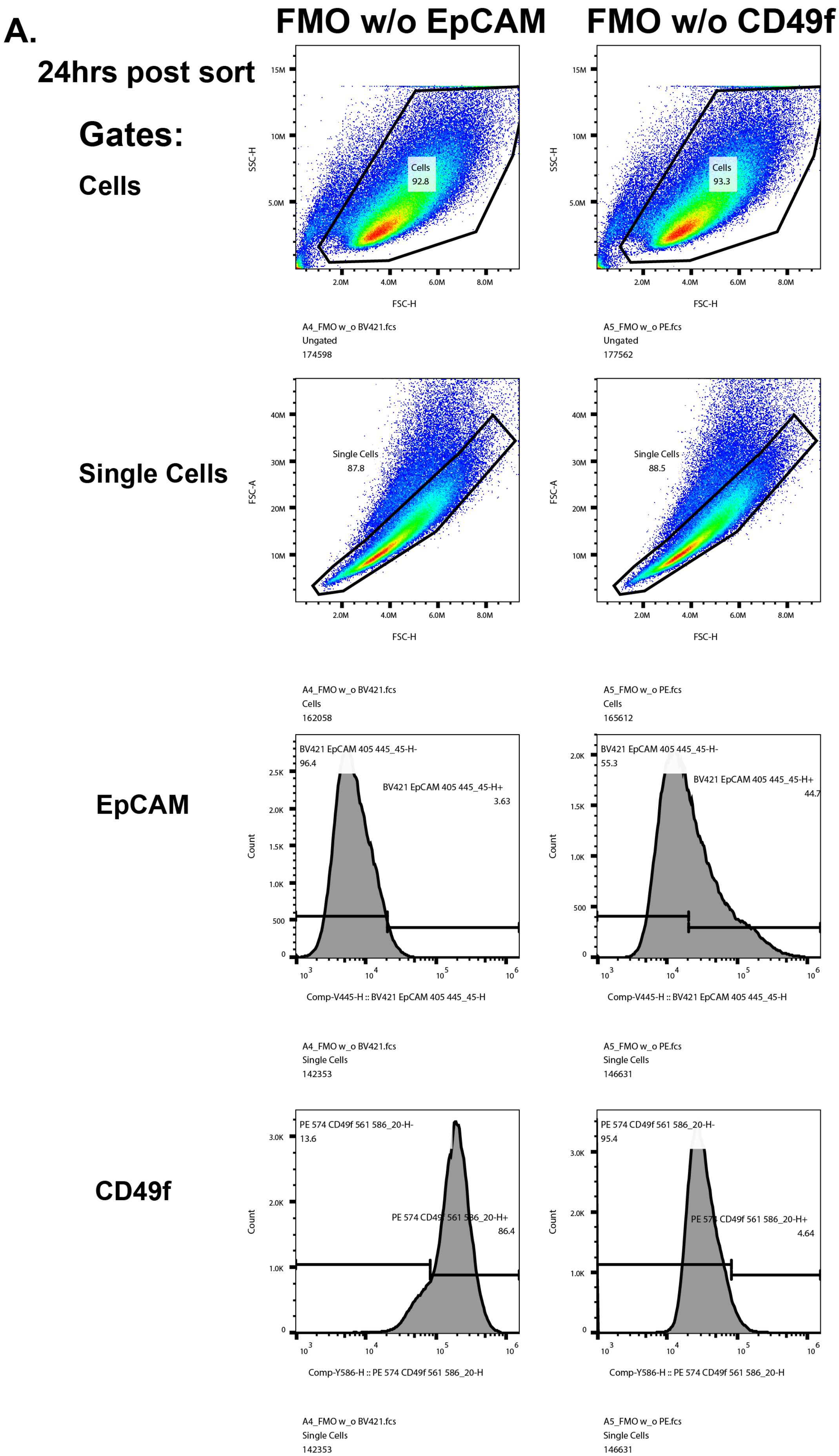
A.
FMO w/o EpCAM
FMO w/o CD49f
24hrs post sort
Gates:
Cells
Single Cells
EpCAM
CD49f
Cells
92.8
Cells
93.3
SSC-H
FSC-H
15M
10M
5.0M
2.0M
4.0M
6.0M
8.0M
A4_FMO w_o BV421.fcs
Ungated
174598
A5_FMO w_o PE.fcs
Ungated
177562
Single Cells
87.8
Single Cells
88.5
FSC-A
40M
30M
20M
10M
A4_FMO w_o BV421.fcs
Cells
162058
A5_FMO w_o PE.fcs
Cells
165612
BV421 EpCAM 405 445_45-H-
96.4
BV421 EpCAM 405 445_45-H+
3.63
BV421 EpCAM 405 445_45-H-
55.3
BV421 EpCAM 405 445_45-H+
44.7
Count
Comp-V445-H :: BV421 EpCAM 405 445_45-H
A4_FMO w_o BV421.fcs
Single Cells
142353
A5_FMO w_o PE.fcs
Single Cells
146631
PE 574 CD49f 561 586_20-H-
13.6
PE 574 CD49f 561 586_20-H+
86.4
PE 574 CD49f 561 586_20-H-
95.4
PE 574 CD49f 561 586_20-H+
4.64
Comp-Y586-H :: PE 574 CD49f 561 586_20-H
A4_FMO w_o BV421.fcs
Single Cells
142353
A5_FMO w_o PE.fcs
Single Cells
146631

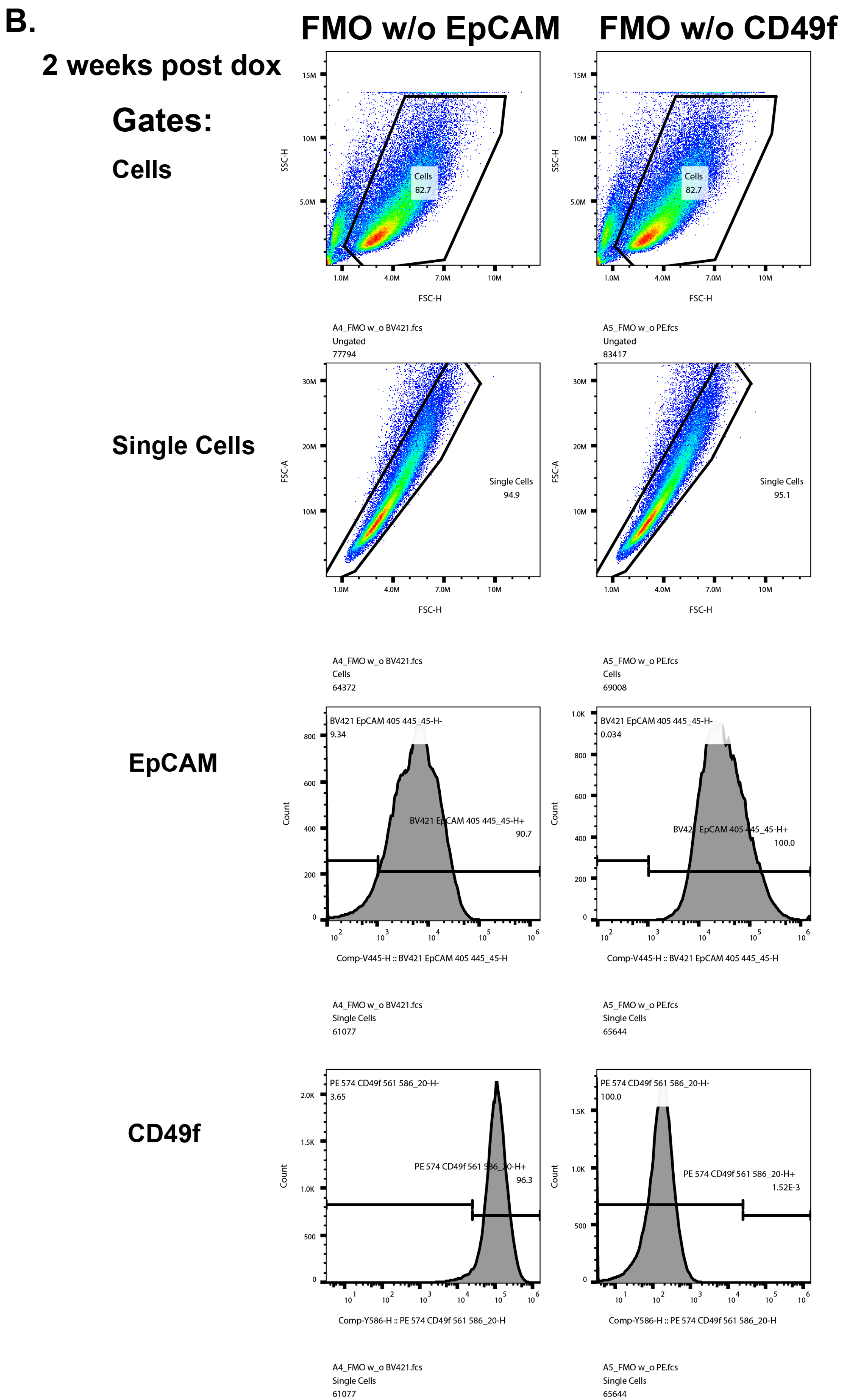
B.
FMO w/o EpCAM
FMO w/o CD49f
2 weeks post dox
Gates:
Cells
Single Cells
EpCAM
CD49f
SSC-H
FSC-H
Cells
82.7
A4_FMO w_o BV421.fcs
Ungated
77794
A5_FMO w_o PE.fcs
Ungated
83417
FSC-A
Single Cells
94.9
Single Cells
95.1
A4_FMO w_o BV421.fcs
Cells
64372
A5_FMO w_o PE.fcs
Cells
69008
Count
BV421 EpCAM 405 445_45-H-
9.34
BV421 EpCAM 405 445_45-H+
90.7
BV421 EpCAM 405 445_45-H-
0.034
BV421 EpCAM 405 445_45-H+
100.0
Comp-V445-H :: BV421 EpCAM 405 445_45-H
A4_FMO w_o BV421.fcs
Single Cells
61077
A5_FMO w_o PE.fcs
Single Cells
65644
PE 574 CD49f 561 586_20-H-
3.65
PE 574 CD49f 561 586_20-H+
96.3
PE 574 CD49f 561 586_20-H-
100.0
PE 574 CD49f 561 586_20-H+
1.52E-3
Comp-Y586-H :: PE 574 CD49f 561 586_20-H
A4_FMO w_o BV421.fcs
Single Cells
61077
A5_FMO w_o PE.fcs
Single Cells
65644

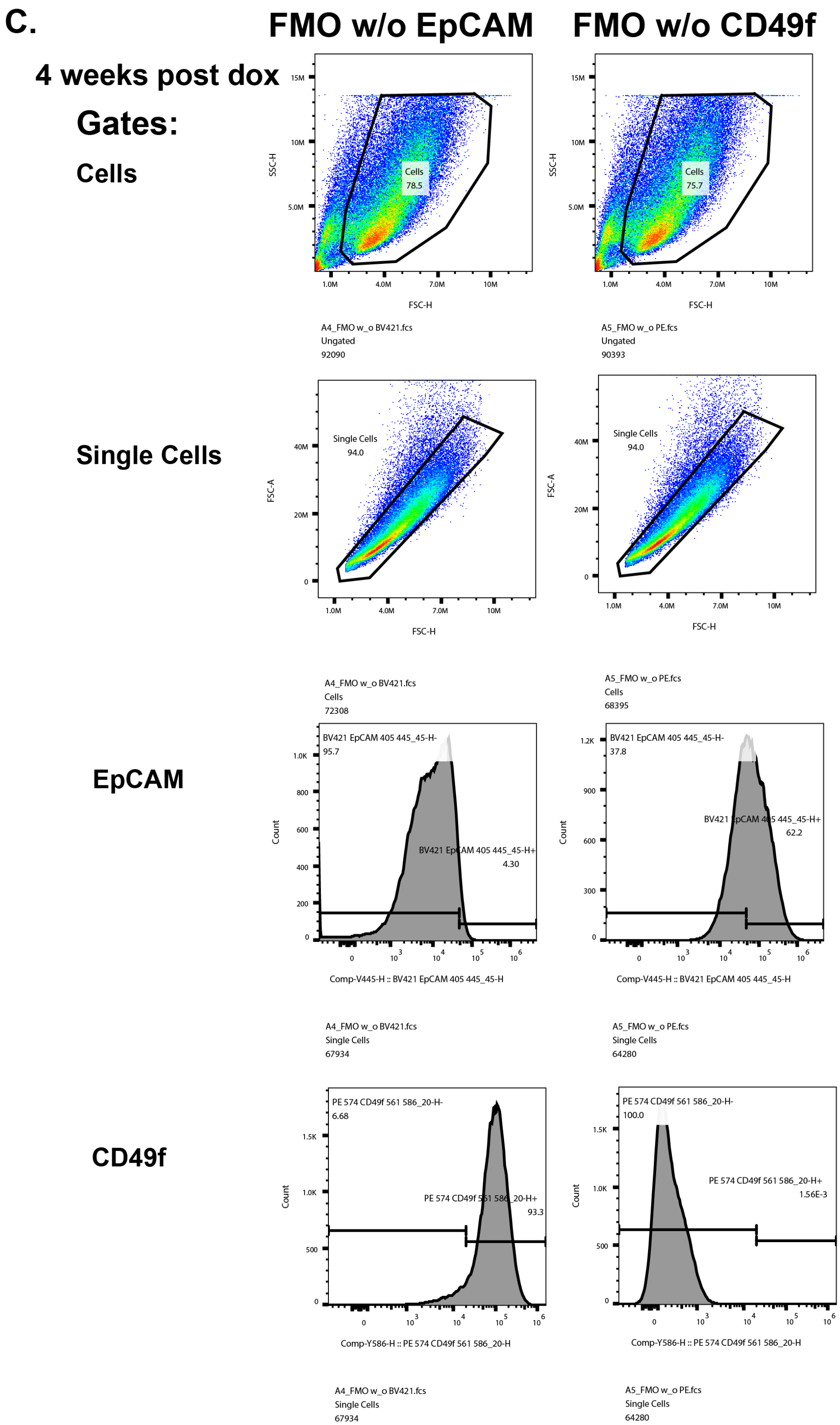

Supplemental Figure 8. Gating for flow analyses after flow sort. A-C) Fluorescence minus one (FMO) samples were used to set the gates for EpCAM and CD49f for all three post sort analyses including A) 24hrs post sort, B) 2 weeks post dox, and C) 4 weeks post dox. Gates for each day include Cells (SSC-H vs. FSC-H), Single Cells (FSC-A vs. FSC-H), EpCAM (Count vs. EpCAM-BV421), and CD49f (Count vs. CD49f-PE). The samples used to set the gates include a mixture of cells from each sorted population (NTG EpCAM Hi, NTG EpCAM Lo, *CDH1*-KO EpCAM Hi, and *CDH1*-KO EpCAM Lo). The left column shows the FMO without EpCAM antibody and the right column shows the FMO without the CD49f antibody. Antibody details can be found in methods.
