# Supplementary material for: CDH1 loss remodels gene expression and lineage identity in human mammary epithelial cells
Source: PLoS Genet. 2026 Oct 27;22(10):e1012351. doi: 10.1371/journal.pgen.1012351 (PMC13722294; doi:10.1371/journal.pgen.1012351)
Supplement: Supplementary file 9 [file pgen.1012351.s009.pdf]

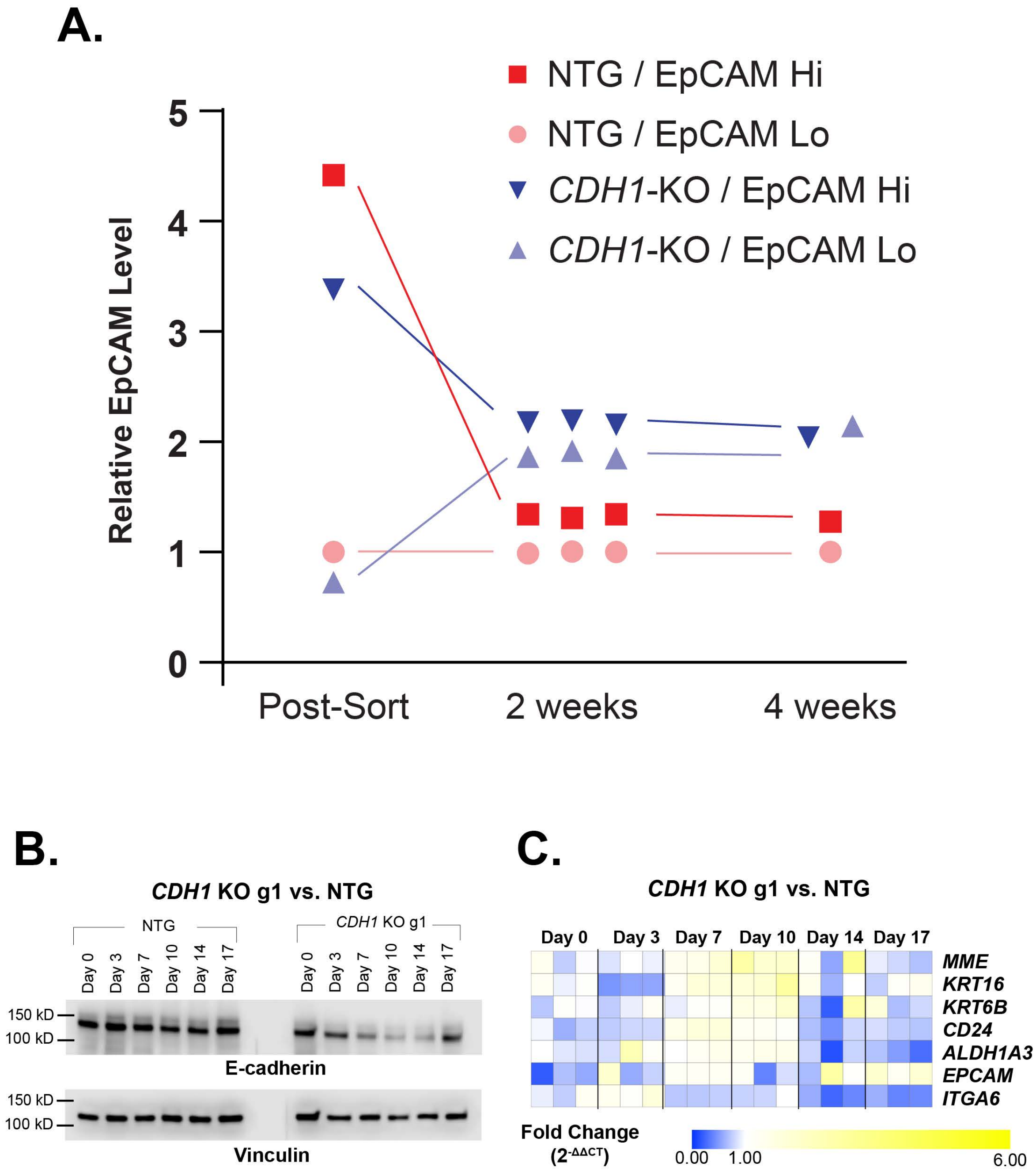

Supplemental Figure 9. *CDH1*-KO Time Course with *CDH1*-KO g1. A) Relative EpCAM levels (NTG/EpCAM low as baseline) in post-sorting populations over a complete timecourse; results shown are for a single timecourse experiment and representative of repeated experiments (see Figure 6). Points represent biological replicates. B) Time course western blots of E-cadherin with Vinculin loading control. C) Paired time course samples analyzed via qPCR for basal and luminal gene expression.
