# Supplementary material for: CDH1 loss remodels gene expression and lineage identity in human mammary epithelial cells
Source: PLoS Genet. 2026 Oct 27;22(10):e1012351. doi: 10.1371/journal.pgen.1012351 (PMC13722294; doi:10.1371/journal.pgen.1012351)
Supplement: Supplementary file 10 [file pgen.1012351.s017.pdf]

Matthew J. Sikora, PhD
Associate Professor, Department of Pathology
University of Colorado Anschutz

Mail Stop 8104
Room 5110; 12801 E. 17th Ave | Aurora, CO 80045
o 303 724 4301 | matthew.sikora@cuanschutz.edu
sikoralab.com

September 12, 2026

Re: Revised manuscript for "*CDH1* loss remodels gene expression and lineage identity in human mammary epithelial cells" (PGENETICS-D-26-00592)

Dear editor,

Thank you for the opportunity to submit a revised manuscript incorporating reviewer feedback. We are pleased about the reviewers' enthusiasm for our study, and grateful for their thorough and thoughtful suggestions and requests. We would like to particularly note that the clarity of reviewer suggestions was extremely valuable, and we genuinely thank both reviewers for their help strengthening this manuscript.

Please find below a point-by-point response to reviewer comments, which details our updates in this revised manuscript. We have included a version with tracked changes in the re-submission to facilitate review of the changes made.

Sincerely,

Matthew J. Sikora, PhD

Reviewer 1:

1. The antibody-versus-genetic comparison is central and should be more carefully framed.

A major strength of the manuscript is the comparison between functional E-cadherin disruption by antibody inhibition and genetic CDH1 loss by siRNA or CRISPR/Cas9. This comparison underlies one of the most important conclusions of the paper: that antibody-mediated disruption of cell-cell contacts induces EMT, whereas genetic CDH1 loss represses EMT and promotes luminal lineage remodeling.

This is a compelling concept, but the comparison carries some inherent differences that should be acknowledged more explicitly. The antibody treatment is acute, whereas the genetic perturbations occur over different timescales, especially in the two-week inducible CDH1-KO system. Thus, the observed differences may reflect not only the mode of E-cadherin targeting but also the distinction between acute junctional disruption and longer-term adaptation to genetic CDH1 loss.

In addition, the Methods indicate that the HECD-1 antibody analysis was complicated by an unexpected response to the IgG control, leading the authors to derive HECD-1-specific DEGs by subtracting the IgG-versus-control gene list from the HECD-1-versus-control gene list. This is a reasonable analytical solution, but readers would benefit from seeing the magnitude and nature of the IgG response more directly.

Specifically, I suggest that the authors:

• Acknowledge more explicitly that antibody-mediated E-cadherin disruption is acute, whereas siRNA- and CRISPR/Cas9-mediated CDH1 loss occur over different and longer timescales.

• Clarify that the observed differences may reflect both the mode of E-cadherin targeting and the time allowed for cellular adaptation.

• Provide, perhaps in the supplement, the number of IgG-responsive genes and the major pathways enriched in the IgG-versus-control comparison.

• Temper language that presents the antibody and genetic arms as simple binary opposites, and instead frame the findings as evidence that acute E-cadherin contact disruption and genetic CDH1 loss produce distinct cellular states.

I do not think this issue undermines the core conclusion, especially because the genetic CDH1-loss data are supported by multiple orthogonal approaches. However, addressing these points would make the central comparison clearer and more convincing.

We appreciate these suggestions that will improve interpretability of our results. In response, we have:

- Specifically noted that time scales for the *CDH1*-targeting approaches are distinct and that this may impact cellular adaptation, in line 129-131;
- Included a new Supplemental File 1 that lists differential gene expression for all comparisons performed herein, including IgG-treated vs control (referenced in Results line 139, Methods, line 505-506);
- Updated a comment in the discussion suggesting an opposite effect between the targeting approaches, which now reads: “Indeed, acute E-cadherin contact disruption and genetic *CDH1* loss produce distinct cellular states, in particular related to EMT signatures, supporting that *CDH1* loss in ILC is a mechanistically distinct event in breast cancer development and progression.” (Line 364-367).

2. The cross-model and cross-method structure should be made more explicit.

One of the manuscript's strengths is its breadth. However, because different experimental arms are performed in different HMEC models and with different perturbation strategies, the reader is sometimes asked to integrate findings across several systems. For example, the single-cell RNA-seq and much of the phenotypic work are centered on CDH1-KO in 184D1 cells, whereas the ATAC-seq is performed after siRNA-mediated CDH1 knockdown in 240D1 and 153D1 cells. The antibody comparison is performed in another subset of models.

This is not necessarily a weakness; indeed, concordance across multiple HMEC backgrounds supports the generality of the findings. However, the manuscript would benefit from a clearer summary of which conclusions are supported in which model and by which perturbation.

To improve clarity, I suggest that the authors:

• Add a summary table or schematic indicating which conclusions are supported by which HMEC model, perturbation strategy, and assay.

• For each major claim, indicate whether it is supported by antibody inhibition, siRNA knockdown, CRISPR/Cas9 knockout, bulk RNA-seq, single-cell RNA-seq, ATAC-seq, flow cytometry, sorting, or functional assays.

• Clarify when findings are robust across multiple starting lineage states versus inferred primarily from one model.

• More explicitly discuss how baseline luminal/basal composition differs among HMEC models, particularly the relatively luminal baseline state of 240D1 cells versus the more basal-enriched baseline state of 184D1 cells.

• If existing data permit, provide a targeted concordance check between the ATAC-derived transcriptional program and the 184D1 CDH1-KO model that carries much of the functional weight.

• For example, the authors could show whether the NFI/KLF/TFAP2C-associated program or related candidate transcriptional modules identified from ATAC-seq are similarly induced in the 184D1 CDH1-KO system.

Such a table or schematic would help readers track the evidentiary basis for each conclusion and better integrate the chromatin, transcriptomic, and phenotypic arms of the study.

We appreciate these suggestions that will improve data integration across study arms and models. In response, we have:

- Included Supplemental File 5, which identifies 6 major conclusions from this study (listed below), and for each supporting experiment from the primary manuscript figures, identifies the analysis/assay, the method of E-cadherin suppression, the models utilized, and the associated figure. This summary file is called out in the first paragraph of the discussion, line 351-352.

- More directly discussed that the HMEC models carry distinct baseline populations, in line 150-151: "Importantly, these trends were observed across all 4 of the HMEC models utilized, which each carry distinct baseline luminal/basal composition (Supp. Fig. 4A)."

Regarding the concordance check, we appreciate this suggestion as we regrettably encountered technical issues with performing ATACseq in the 184 models. In Supplemental File 3, we assessed enrichment for ENCODE regulatory genesets in differentially expressed genes in the 184D1 CDH1-KO models. Among enriched motifs in Figure 6H, this ENCODE series includes consensus regulatory target genesets for TP53, TP63, KLF4, FOS, and ATF3; TP63, FOS, and KLF4 were each enriched with both gRNA KO's, supporting the ATACseq data in 153/240 siRNA models. This observation has been added to the Results, in line 228-232.

3. The LCIS clinical analysis is valuable but should be interpreted as supportive rather than causal.

The LCIS RNA-seq analysis is a valuable addition, particularly because clinical transcriptomic datasets of lobular precursor lesions are limited. The finding that LCIS samples show increased luminal-associated genes and decreased basal/EMT-associated genes provides an important clinical parallel to the HMEC models.

However, this analysis is necessarily correlative. The comparison is between LCIS, which is already E-cadherin-negative and neoplastic, and normal breast tissue. Thus, the luminal signature could reflect the intrinsic luminal/ER-positive nature of lobular neoplasia, enrichment for neoplastic epithelium, differences in epithelial composition, or tissue-composition differences between LCIS and reduction mammoplasty specimens, rather than being directly attributable to CDH1 loss alone.

I suggest that the authors:

• Frame the LCIS analysis as consistent with the HMEC model rather than as independent causal evidence that CDH1 loss drives luminal reprogramming in vivo.

• Distinguish clearly between the causal HMEC perturbation experiments and the correlative clinical LCIS comparison.

• Provide additional information about the LCIS cohort where possible, including ER status, patient age, lesion type, classic versus pleomorphic status, and an estimate of epithelial/neoplastic cellularity.

• Discuss epithelial fraction or tissue-composition differences between LCIS and normal breast samples if such information is available.

• Clarify that comparisons to DCIS and EMT activation in ductal lesions are literature-based unless DCIS is analyzed directly in the same framework.

This distinction is important because the HMEC perturbation experiments are the causal arm of the study, whereas the LCIS dataset provides clinical concordance. The LCIS data are still valuable, but the interpretation should be appropriately calibrated.

We agree with the reviewer that our LCIS analyses are correlative rather than providing causal evidence regarding CDH1 loss and lineage reprogramming, and appreciate the suggestions for reframing these analyses/conclusions. In response to the above concerns, in the revised manuscript, we:

- Expanded Supplemental File 4 to include additional clinicopathological data from samples assessed herein, including patient age, histology/pathology assessment data, ER/PR/HER2 status, and estimates of cellularity, to the full extent that these data were available.
- Updated the results section to more explicitly address the correlative nature of this analysis and the literature observations on DCIS, now in lines 255-261:
    - “Importantly, the observed gene expression differences are correlative with CDH1 loss in early lobular neoplasia, yet may reflect other features of oncogenesis, e.g. increased epithelial cellularity in LCIS. However, the shift we observe in LCIS contrasts data from ductal carcinoma in situ (DCIS) in the literature; Rebbeck et al assessed gene expression in samples from n=145 patients with DCIS, and identified EMT activation in the progression of DCIS to invasive disease [62]. Taken together, LCIS gene expression data are consistent with the changes seen in our CDH1 loss models in HMEC, and support that early ILC oncogenesis includes a cell lineage shift to a luminal phenotype.”
- We similarly updated the discussion section on this point, in lines 356-358:
    - “Importantly, gene expression changes in clinical samples of LCIS, which presented an increase in luminal markers and decrease in basal markers relative to normal breast tissue, were consistent with the gene expression changes seen in HMEC models upon CDH1 loss that show remodeling toward a luminal lineage phenotype.”

4. Reprogramming versus selection should be discussed carefully in the proliferation data.

The population-doubling data support the idea that CDH1 loss provides a growth or persistence advantage in finite-lifespan HMEC cultures. This is highly relevant to ILC initiation, where early CDH1-deficient cells must persist and expand despite loss of epithelial adhesion.

However, because the CDH1-KO cultures are mixed populations, the increased population doublings could reflect a cell-autonomous proliferative effect of CDH1 loss, selective outgrowth of edited/E-cadherin-low cells, or both. This distinction bears directly on the authors' model of CDH1 loss as a lineage-remodeling event that provides clonal advantage.

To clarify this point, I suggest that the authors:

• Explicitly acknowledge that increased population doublings in mixed KO cultures may reflect a combination of lineage reprogramming and selection.

• Clarify whether the E-cadherin-negative or edited fraction changes over serial passage, if such data are already available.

• If feasible with existing material or straightforward follow-up experiments, track the fraction of E-cadherin-negative or edited cells over serial passage.

• If the E-cadherin-negative fraction increases over time, discuss this as support for a selection model.

• If the E-cadherin-negative fraction remains stable while the population expands more robustly, discuss this as support for a more cell-autonomous proliferative effect.

• Clarify the statistical tests used in the population-doubling analysis, as the figure legend appears to refer to both slope comparisons and ANOVA-based comparisons.

I do not view serial tracking as essential if the authors instead clearly acknowledge this limitation in the Discussion. Either interpretation (selection, reprogramming, or a combination of both) would be interesting and relevant to the authors' model.

We appreciate and agree with these comments. While we do not have data regarding endpoint E-cadherin levels for our population doublings experiments, immunoblots in Figure 6F support that E-cadherin levels are stable for at least ~10 days, which would account for 2-3 passages. Absent additional data, the limitations noted above (along with related comments from Reviewer 2) are now addressed in the Discussion, lines 419-430:

"A key consideration for our experimental models of genetic CDH1 loss are related to incomplete E-cadherin loss with siCDH1, and heterogenous E-cadherin loss in our Cas9 CDH1-KO models, both of which likely lead to mixed populations of E-cadherin-positive, -low, and -negative cells in our CDH1-loss studies. Directly confirming E-cadherin-negative status in post-CDH1-KO settings, i.e. in the induced EpCAM-high and/or CD10-high populations, would provide a stronger link between CDH1-loss and the identified phenotypes. However, technical limitations with flow cytometry assays (E-cadherin required fixed cells; lineage markers required fresh cells) precluded such direct measurements in our experiments. Similarly, the contributions of lineage remodeling versus selection, in particular in serial passage and longer-term assays, would be needed to define whether CDH1 loss provides a selective advantage to cells. Importantly, our use of multiple HMEC models with differing baseline lineage populations and a series of orthogonal studies (Supplemental File 5) underpins support for the role of CDH1 loss in mammary cell lineage and ILC oncogenesis."

Regarding the statistics described, the one-way ANOVA and stated p-values were in regard to panel D, and listed after the description of the slope comparisons in error. This has been corrected in the revised manuscript.

5. The lineage-sorting experiment is a major strength and should be emphasized, but reporting details should be expanded.

The lineage-sorting experiment is one of the strongest parts of the manuscript because it directly addresses a key alternative explanation: that the apparent luminal shift after CDH1 loss reflects selective expansion of pre-existing luminal cells rather than remodeling of lineage state. The finding that both sorted luminal and sorted basal populations converge toward an EpCAM-high state after CDH1-KO is important and should be highlighted more prominently in the Results and Discussion.

This experiment is especially important because it is the strongest evidence in the manuscript against a purely compositional-selection explanation for the luminal shift. Because it carries substantial interpretive weight, the authors should provide more methodological and quantitative detail.

Specifically, I suggest that the authors clarify:

• The number of independent sorting experiments performed.

• The number of biological and technical replicates for each sorted population.

• The sort purity for luminal and basal fractions.

• How the flow-cytometry gates were defined.

• Whether gates were based on FMO controls, unstained controls, prior literature, or another strategy.

• Why the reported n appears to differ across panels in Fig. 6.

• Whether the sort-efficiency check, which appears to be n = 1, was intended as a representative validation rather than a replicated experiment.

Similarly, for the colony-forming/progenitor assays in Fig. 5, please report:

• The number of colonies scored.

• The number of independent experiments.

• Whether colony phenotypes were scored blinded.

• The statistical test used for phenotype proportions.

• Whether representative images correspond to quantified experiments.

These assays provide important functional support for the luminal progenitor-like interpretation, so transparent reporting will strengthen the manuscript.

We appreciate the reviewer's enthusiasm for these experiments and are pleased to provide additional experimental detail for more transparent reporting.

For both the lineage/sorting experiment (Figure 6A-E) and colony phenotype assays (Figure 5D-G), we reported additional detail in both the associated figure legend and the pertinent part of the Methods section (lines 650-652). Briefly, the latter represents three complete independent experiments, with the images corresponding to those included in quantification, with two-way ANOVA used for statistical testing. Scoring was not blinded, but was independently performed by two investigators to affirm phenotype calls. For the former, sorting timecourse data in 6D-E is from an individual experiment; biological triplicate samples at 2 weeks (Supplemental Figure 9) were pooled for 4 week analysis due to sample limitations. Data are representative of a series of repeated studies of the overall post-knockout timeframe. The sorting efficiency check is representative validation of our gating strategy, which is based on a combination of FMO controls (Supplemental Figure 8) and basal/luminal population distributions in the overall HMEC series (Supplemental Figure 4); relative sort efficiency is shown as percent quadrant populations in Figure 6C. The legend for Figure 6 has been updated to reflect the above details.

Please also see Reviewer Figure 3 below (in Reviewer 2 response), which provides orthogonal support for the lineage/sorting experiment results.

6. The ILC-specific model would benefit from clearer distinction between instructive, selective, and permissive roles of CDH1 loss.

The manuscript makes a compelling case that genetic CDH1 loss can remodel HMEC lineage identity toward a luminal progenitor-like state. However, because ILC is overwhelmingly luminal/ER-positive and CDH1 loss is early but not sufficient for full malignant transformation, it would be helpful for the Discussion to distinguish among several related possibilities.

The authors should discuss whether CDH1 loss may:

- *Instruct a luminal progenitor-like state.*
- *Select for cells already compatible with luminal/ER-associated programs.*
- *Stabilize or permissively expand a luminal progenitor-like state.*
- *Require cooperation with additional ILC-associated events, such as PI3K pathway alteration, ER pathway activity, CCND1 gain, or other lesions.*

This distinction would make the model more nuanced and more directly anchored in ILC biology. The use of CCND1-overexpressing post-stasis HMECs is appropriate and relevant, particularly given the frequency of CCND1 gain in ILC, but it also means that the effects of CDH1 loss are being studied in a partially primed hyperplastic context rather than in fully naïve mammary epithelium.

I suggest that the authors:

- *Discuss the CCND1-overexpressing post-stasis HMEC context as an important feature of the model rather than as a weakness.*
- *Clarify how this model relates to early lobular oncogenesis.*
- *Avoid implying that CDH1 loss alone is sufficient for full malignant transformation.*
- *More explicitly state that CDH1 loss may create a permissive or advantageous lineage state that requires additional oncogenic events.*

We appreciate the suggestion and opportunity to expand the discussion related to the important nuance for the model we used and the genetic implications for downstream oncogenesis. We are continuing to explore in particular the cooperation between *CDH1* loss and additional oncogenic hits, both as preceding initiating events versus downstream cooperative events, and look forward to sharing these data in the future. In the meantime, we have added a paragraph in the discussion to address this comment, lines 377-391:

"These observations highlight that lineage remodeling upon CDH1 loss, and subsequent ILC oncogenesis, may yet reflect one or more related scenarios: 1) CDH1 loss stabilizes or permits expansion of a luminal progenitor-like state; 2) CDH1 loss directly instructs a luminal progenitor-like state in mammary epithelium; 3) CDH1 loss selects for cells compatible with luminal (i.e. ER-positive)-associated programs. Scenarios 1 and 2 may work in concert; our findings support that direct remodeling and expansion both occur to create a population of cells primed to acquire additional transforming hits. Scenario 3 is likely associated with the insufficiency of CDH1 loss alone to drive transformation and the distinct repertoire of tumor mutations associated with ILC. In that context, our use of CCDN1-overexpressing HMEC (in addition to serving an important technical role in facilitating passage) is likely important in cooperating with CDH1 loss to drive early ILC transformation phenotypes. CDH1 loss may cooperate with ILC-enriched oncogenic hits (FOXA1, PTEN, TBX3, ARID1A, AKT1, CCND1) and antagonize ILC-depleted hits (GATA3, MAP3K1, MAP2K4). Similar findings in lung cancer models support that cell lineage defines tolerance for specific oncogenic hits; lung alveolar cells are transformed by EGFR but killed by Myc activation, with the converse in basal/neuroendocrine cells. Future work will address how CDH1 loss creates this primed state versus leverages specific states (e.g. CCND1 gain) to drive ILC oncogenesis."

Regarding any suggestion that CDH1 loss is sufficient for transformation, we wholly agree as extensive in vivo GEMM data and our results strongly support that CDH1 loss is insufficient for transformation. We revised discussion of GEMMs in the Introduction in line 101: "…genetically engineered mouse models (GEMMs; Cre/loxP, *Cdh1*$^{flox/flox}$ plus additional oncogenic hits)…".

Minor Comments

1. Data availability. The statement indicates that RNA-seq and ATAC-seq datasets are being uploaded to GEO. Because these sequencing datasets are central to the manuscript's claims, GEO accession numbers and reviewer tokens should be provided during revision or before acceptance. This should include:

• Bulk RNA-seq datasets.

• ATAC-seq datasets.

• Single-cell RNA-seq datasets.

• LCIS RNA-seq datasets, as applicable.

• Supplemental data files required to reproduce the analyses.

The new Supplemental File 1 includes DEGs for RNA-seq in Figure 1, to facilitate analyses by readers. GEO datasets completed upload and processing during review/revision, and have been identified in the Methods under 'Data Availability':

"Sequencing data associated with these studies (HMEC RNA-seq, LCIS RNA-seq, HMEC scRNA-seq, HMEC ATAC-seq) are available in GEO super series GSE346960, which includes the following datasets: GSE346801 (RNA-seq HECD1); GSE346797 (RNA-seq siRNA); GSE347020 (RNA-Seq CDH1-KO); GSE346457(scRNA-Seq); GSE346452 (ATAC-Seq); GSE346959 (RNA-seq LCIS)."

Please note that data are current embargoed pending manuscript acceptance, but can be accessed by reviewers using token **sjijkkuyhxmfrwt** at https://www.ncbi.nlm.nih.gov/geo/query/acc.cgi?acc=GSE346960.

2. Supplementary figure cross-references. Please check the cross-references for FMO gating controls. The text and figure legends appear to refer to different supplementary figure numbers.

Thank you for catching this error; we have reviewed and updated figure and data callouts throughout the manuscript.

3. Cell-line nomenclature. Please standardize HMEC line names throughout the manuscript. For example, 184D-D1L, 184D1, 240L-D1, 240D1, and possible variants in the Methods should be harmonized or clearly defined at first use.

We updated HMEC nomenclature, and added to the methods clarifying the specific sublines used (i.e. those provided by LBNL) and their associated nomenclature in lines 450-455: "HMEC lines were derived from patient/specimen numbers 184, 122, 153, and 240 [PMID 22552289]. Specific sublines are identified by their specimen number and Left/Right breast as needed, batch indicator if relevant, a dash (-) followed by the agent/gene used to induce post-stasis growth (-D1 for CCND1-overexpression), and any additional processing indicators. HMEC sublines used herein were specifically: 184D-D1L (batch D, enriched for luminal phenotype after -D1 expression); 122L-D1 (left breast); 153L-D1 (left breast); 240LB-D1 (left breast, batch B)."

4. Define terms for a broad genetics readership. Terms such as "post-stasis," "stasis," "NTG," and "M87A medium" should be defined at first mention or briefly cited.

We reviewed the manuscript for potentially unclear terminology and made the following updates:

- "Stasis", line 117-119: "We used variant HMECs that were altered in culture to overcome stasis (i.e. stress-associated senescence barrier to continuous proliferation) via overexpression of CCND1".
- "NTG": Updated the Figure 1 legend, "siNT, non-targeting siRNA. NTG, KO g1, and KO g2 refer to non-targeting Cas9 gRNA, CDH1-targeting gRNA-1, and CDH1-targeting gRNA-2, respectively (see Methods)" and Methods, line 471-473: "1 non-targeting gRNA (NTG) control (Lenti CRISPR Universal Non-Target Control) +2 CDH1-targeting gRNA (g1 – Clone ID 236996584, and g2 – Clone ID 236987757; lentiCRISPRv2-puro). CDH1-targeting gRNAs target exon 3 (g1) and exon 2 (g2).", and Results line 163-164: "We compared HMEC 184D1 non-targeting gRNA control (NTG) versus CDH1-KO g2 (referred to as CDH1-KO in this analysis)".
- "ATAC-seq": updated Results line 191-193: "we examined changes in the chromatin landscape caused by CDH1 loss via ATAC-seq (Assay for Transposase-Accessible Chromatin Sequencing)".

- “BETA-minus”: updated Figure 3 legend: “(BETA-minus pipeline, see Methods)”.
- “M87A medium”: updated Methods to clarify that the medium mixture used for HMEC culture is called M87A; clarified description of the mixture.
- “i-cisTarget”: added hyperlink for motif analysis tool in ATAC-seq section in Methods.

5. ID2 model. The Discussion presents an interesting model involving ID2 and luminal progenitor-like remodeling. This is compelling, but the functional role of ID2 is not directly tested in the current manuscript. Please frame ID2 as a strong candidate or future direction unless additional functional data are added.

We would decline to include additional data on ID2 given the breadth of the manuscript, and absent these data have pared back the related discussion in lines 392-403:

“Defining the transcription or epigenomic factors that mediate changes in gene expression and lineage remodeling upon CDH1 loss is a key next step in understanding the role of CDH1/E-cadherin as a tumor suppressor in ILC oncogenesis. One such factor identified as a candidate in our studies is inhibitor of DNA binding 2 (ID2). ID2 regulates commitment to luminal lineage [70], and has been shown to be transcriptionally induced upon E-cadherin loss whereupon it drives anoikis resistance in multiple models of ILC [6]; nuclear roles of ID2 are less well understood [6]. ID2 indirectly binds DNA via basic helix-loop-helix (bHLH) and ETS family transcription factors [71], which were among gained peaks in ATAC-seq upon CDH1 (TFAP2C, ELK4, GABPA). Conversely, CDH1 loss reduced accessibility at p63 motifs, consistent with the role of p63 as a basal transcription factor [74], yet whether p63 is directly or indirectly regulated by CDH1 loss is unknown. Taken together, changes in chromatin accessibility and associated gene expression point toward potential factors that mediate luminal reprogramming upon CDH1 loss, representing an important area of ongoing investigation.”

6. Cell-cycle/proliferation validation. The single-cell analysis suggests increased S/G2/M fractions after CDH1 loss. If available, an orthogonal readout such as EdU, Ki67, or cell-cycle flow analysis would strengthen the connection between the single-cell data and the population-doubling assay. If not available, the computational nature of the cell-cycle inference should simply be stated.

Regrettably, we do not have orthogonal readouts for cell cycle in these studies, and have updated the text in lines 167-169 and 178-180 to emphasize that cell cycle is imputed from gene expression data.

7. Pleomorphic LCIS. The handling of pleomorphic LCIS samples should be stated clearly in the Results where the LCIS analysis is introduced. Given the small number of pLCIS cases, exclusion after clustering may be appropriate, but the rationale should be transparent.

We clarified our rationale for excluding the small number of pLCIS cases in cohort in lines 244-247:

“Of note, 4 of 22 LCIS were considered to have pleomorphic histology. While pleomorphic LCIS (pLCIS) clustered with classic LCIS (n=18) by these lineage markers, pLCIS likely represents a distinct oncogenic trajectory [PMID 1970107, 31932682], and as such were excluded in further analysis.”

8. Statistical thresholds. Different adjusted p-value or q-value thresholds are used across panels. Please include a brief statement in the Methods explaining the rationale for panel-specific thresholds and the correction methods used.

The figure legend for Figure 1A has been updated to clarify the selection of adj.p cutoffs:

“Venn diagrams show the overlap of differentially expressed genes (DEG), and the adj. p value cutoff used to determine gene lists used for B-E. Adj.p cutoffs were selected in each method to obtain similar numbers of DEGs to prevent biases in downstream gene set analyses from longer vs shorter DEG genesets.”

9. Motif-analysis thresholds. The motif analysis appears to use different NES thresholds for gained versus lost peaks. Please justify the asymmetric thresholds or state whether the qualitative conclusions are robust to matched thresholds.

An NES>3 threshold was used for both gained and lost peaks, the differential cutoff listing was in error and the Figure 3 legend has been corrected accordingly. The motif listings in Supplemental File 3 show the NES each enriched motif. We appreciate the reviewer identifying this error.

10. Anoikis assay controls. The anoikis-resistance assay is highly relevant to ILC biology and is one of the manuscript's strongest functional links. Please state viability at seeding and clarify whether any additional anoikis-sensitive or positive-control condition was included. If not, this could be acknowledged.

The methods section for these studies (lines 654-662) has been updated to reflect that seeding was normalized across conditions using trypan blue-negative cell counts (i.e. viable cell counts), and that additional treatments to induce cell death were not included in these assays.

11. Typographic/reference cleanup. Please check reference formatting and minor typographic issues. For example, one DOI/reference string appears malformed, and "preformed" should likely be "performed." Also, please confirm that all figure panels render correctly in the review PDF.

We reviewed for typographical errors and cleaned the cited references/bibliography, and figures/panels have been reformatted per journal revised manuscript guidelines.

12. Claims calibration. The Abstract is appropriately hedged in stating that CDH1 loss "may" underpin mechanisms of early ILC oncogenesis. Please ensure that similarly calibrated language is used throughout the Results and Discussion, especially when interpreting the LCIS clinical dataset.

We reviewed related language in the text, and note that responses to reviewer concerns herein secondarily address issues with language calibration.

Reviewer 2

There are a few limitations to the work. This is not to diminish the work done nor to ask for more, but to be acknowledged in the manuscript. Multiple assays are done through this paper, using eg just 1 of the HMEC models with one of the CDH1 manipulations, and these are not always consistent. Most of the assays were done with HMEC 184D1, where CDH1 was genetically knocked out, which is good as it re-capitulates the cancer better, but this is just one line and so some caution required.

We appreciate this concern, and in response have now included Supplemental File 5, which identifies 6 major conclusions from this study, and identifies the analysis/assay for each supporting experiment from the primary manuscript figures, the method of E-cadherin, suppression, the models utilized, and the associated figure. This summary file is called out in the first paragraph of the discussion, line 351-352. Please also see the response to Reviewer 1, Point #2.

The heterogenous HMEC cell populations (mix of luminal and basal) makes some of the interpretation tricky; with the added complexity that the manipulation of CDH1 is not 100% in the lines such that the cell populations are a mix of lineage and a mix of ecad expression, so the data is not necessarily 'clean' (but reflects biology with studying these things in situ). Its therefore not clear if the results directly relate to the loss of CDH1. In this respect I like the data in later figures where Ecadhein expression itself is investigated (in the timeline Fig6F/G; and in the Ecad gating in Fig 7). But this raises a further question about the manipulation of ecad by the different methods:

For siRNA – knock down is incomplete, as is often the case (SuppFig 1B), but do you know if some cells are negative and some are positive here or that all have reduced expression?

For CDH1-KO – this yielded 30-50% of ecad negative cells (SuppFig1D), but is this actually negative or low? looking at the scale on the flow plots, this could be 'low' when compared to the first plot of unstained cells; so does this mean that cells still express low levels of ecad or is there a mixed pop of negative, low and unchanged ecad?

We appreciate these comments and agree with the reviewer's overall comments that the CDH1-targeting methods used likely result in heterogenous populations with regard to E-cadherin status, which are also different in either targeting approach.

For siRNA, since this method does not induce any frameshift mutations to compromise E-cadherin function, we compared relative levels of E-cadherin in HMEC+siRNA to E-cadherin-neg. ILC line MM134 (https://doi.org/10.1101/2023.09.26.559548). At right/upper (**Reviewer Figure 1)**, by flow cytometry, all 240D1 cells with siCDH1 have reduced E-cadherin vs to siNT control; a subset have E-cadherin levels in range of the MM134 cells, supporting that some HMEC are E-cadherin negative after siRNA. MM134 still show increased signal relative to no antibody controls. At right/bottom, in the CRISPR/Cas9 model, overlaying E-cadherin histograms (independent experiment related to Suppl. Figure 1F) more clearly shows E-cad loss to the level of non-stained controls in a subset of cells, consistent with the expected editing efficiency of Cas9. Noted in line 134-137, we interpret this as a mixed population of hemizygous and homozygous knockout after Cas9 induction.

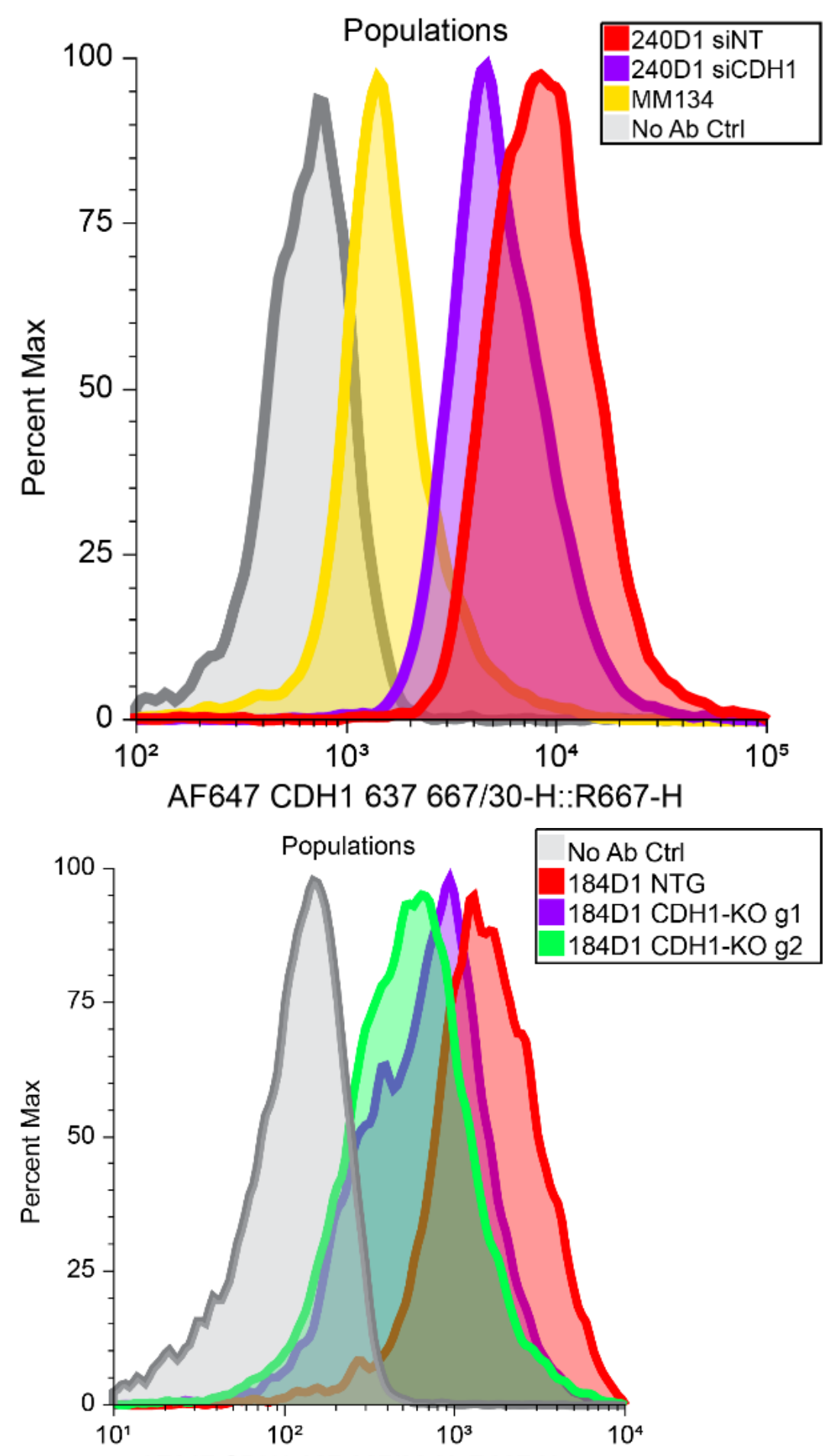

Reviewer Figure 1. E-cadherin flow cytometry.

In response to overall comments here and below regarding the heterogeneity of E-cadherin status in our models, we included an additional paragraph in the Discussion to acknowledge these important considerations, lines 419-430:

"A key consideration for our experimental models of genetic CDH1 loss are related to incomplete E-cadherin loss with siCDH1, and heterogenous E-cadherin loss in our Cas9 CDH1-KO models,

both of which likely lead to mixed populations of E-cadherin-positive, -low, and -negative cells in our CDH1-loss studies. Directly confirming E-cadherin-negative status in post-CDH1-KO settings, i.e. in the induced EpCAM-high and/or CD10-high populations, would provide a stronger link between CDH1-loss and the identified phenotypes. However, technical limitations with flow cytometry assays (E-cadherin required fixed cells; lineage markers required fresh cells) precluded such direct measurements in our experiments. Similarly, the contributions of lineage remodeling versus selection, in particular in serial passage and longer-term assays, would be needed to define whether CDH1 loss provides a selective advantage to cells. Importantly, our use of multiple HMEC models with differing baseline lineage populations and a series of orthogonal studies (Supplemental File 5) underpins support for the role of CDH1 loss in mammary cell lineage and ILC oncogenesis."

Comments on Results

Section 'Genetic loss of E-cadherin represses EMT and shifts cells to a luminal transcriptional state'

Nice data here showing the different modes of ecadherin abrogation and the characterisation of these models by RNAseq.

1. It would be good to add some quantification for the proportion of reduced adhesion and the % knockdown for each line in Supp Fig 1A and B

We corrected the axes in Supplemental Figure 1A, which were labeled as Percent Adhesion but values were for fraction of adhesion. For Supplemental Figure 1B, we added densitometry showing ≥50% E-cadherin knockdown, and added a version of the flow cytometry data shown above as Supplemental Figure 1C to provide additional context to interpret knockdown efficacy.

2. to just clarify - RNAseq was performed on parental and Ecad manipulated cells for each line and each model; and the DEGs were identified between parental vs manipulated and then compared by venn diagrams?

Correct. This is also laid out in the new summary tables in Supplemental File 5 to improve clarity.

Section: 'Single cell analysis supports CDH1-KO-induced reprogramming in luminal and basal cells'. Single cell seq analysis was conducted on 1 HMEC line (184D1) with 1 model of Ecadherin abrogation (KO). Most cells in this line are basal to start with. A mild shift to increase the proportion of cells being 'luminal' and proliferating was observed.

1. Figure 2 – can we see an EMT score in this figure, similar to how you've shown cell cycle/cell identity.

The change in EMT has been included in the text, in lines 166-167: "As in bulk RNA-seq (Fig. 1), the Hallmark EMT signature was repressed across CDH1-KO versus control cells (NES= -1.81; adj. p=0.038)."

2. Given the loss of Ecadherin is heterogeneous in these cells (SuppFig1B) is it possible to see the profile of cells that are Ecadherin negative post knockdown? How do those cells specifically compare to Ecadherin +ve cells of the same phenotype? Ie if Ecadherin loss is in the CDH1-KO luminal cells – can you compare these cells to both Ecadherin-positive luminal NTG cells and CDH1-KO cells for the same features as described (EMT, cell cycle, phenotype markers)?

For single-cell RNAseq, *CDH1* mRNA was strongly reduced in CDH1-KO vs NTG cells (log2FC = -1.17, adj.p < 1e-300; detected in 42% of CDH1-KO cells vs 76% of NTG cells). However, it is not entirely clear how reliably *CDH1* mRNA levels imply E-cadherin status, as the Cas9 editing should in principle not directly impact gene expression or mRNA levels (as opposed to siRNA). Similarly, E-cadherin positive cells in the NTG vs CDH1-KO cells likely reflect wild-type versus hemizygous knockout cells, respectively. Based on these contexts, we did not perform analyses to compare *CDH1* populations, though we strongly agree with the concept suggested by the reviewer.

We also considered using extracellular E-cadherin to sort cells post-knockout, prior to scRNA-seq; however, our primary flow method uses fixed cells and an intracellular E-cadherin epitope, as antibodies targeting extracellular domains proved unreliable in our studies.

3. Note legend for SuppFig6 is referencing part B and C the wrong way around.

This has been corrected in the revision – thank you for catching the error.

4. How does the cholesterol hallmark come about? Im missing the link here.

The increased expression of the Cholesterol Homeostasis Hallmark was a notable output of pathway analyses, but we appreciate that the original text was not clear in that regard. The related text in line 183-185 has been updated: “Notably, in pathway analyses via FGSEA, the cholesterol homeostasis Hallmark was upregulated in CDH1-KO cells in both luminal and basal populations (luminal G1 NES= 2.48, adj. p=0.0027; basal G1 NES= 1.71, adj. p= 0.0706) (Supp. Fig. 6A, C).”

Section: ‘Chromatin landscape supports CDH1 loss-induced changes in cell identity’. Here 2 siRNA HMEC lines were used (240D1 and 153D1) for whole cell population ATAC-seq. ie these are different lines to the line and mode of CDH1 manipulation used in scRNAseq. A small proportion of peaks were changed by siRNA KD, which is perhaps expected. Nevertheless, this still translated to many genes and quite a few changes in common across the 2 lines that had corresponding changes in expression, replicated in all models to some degree, including interesting genes.

Given the heterogeneity of these cell populations (as noted above - mixed populations of basal and luminal cells, mixed populations of Ecadherin negative vs positive cells), could some signal be masked?

Yes, we agree that it is likely that some lineage related shifts are masked, in particular those related to the basal to luminal shift, given that our ATAC-seq data is in cells with a greater proportion of luminal cells at baseline versus the 184D1 models (regrettably, we encountered technical issues in performing ATAC-seq in 184D1). This was reflected in our 240D1 CDH1-KO RNAseq data, wherein the relative gene expression changes were less than other models, but the final gene expression ‘state’ was highly similar to other HMEC (Figure 1C-D; Supplemental Figure 4A-B). However, despite the mixed populations, the extensive overlap in motifs associated with altered peaks in both 153D1 and 240D1, combined with the remodeling we observed in both luminal and basal cells (Figure 6), supports that robust chromatin remodeling impacts of CDH1-KO are likely represented in our data.

Section ‘CDH1 loss in HMEC mirrors gene expression changes in early lobular neoplasia’. 22 LCIS from the archives were analysed by RNAseq against 7 ‘normals’.

1. How was the data quality for these samples, given their age and FFPE nature? Data described certainly looks sensible.

The overall data quality was high, given the FFPE source of the samples; RNAseq data had an overall mapping rate of 97.5% and an average of 27.8 million reads passing bioinformatics filters per sample; this is noted in lines 585-590.

2. How were the 7 control samples prepared? Presumably normal ducts & lobules were dissected to enrich for normal epithelia to match the enrichment for epithelia in the LCIS samples?

The control samples were macro-dissected to enrich for epithelia. Scoring for final nuclei content is not available for these control samples, however, we re-examined putative cell type markers from the RNA-seq. (**Reviewer Figure 2)** While KRT14/KRT18 were differentially expressed in LCIS vs Control/Normal, potentially reflecting lineage changes, KRT17 as a general mammary epithelial marker (PMID: 38548988) was unchanged. Further, COL1A1 (fibroblasts) and ADIPOQ (adipocytes) were also equivalent between LCIS and control. Collectively these marker genes support that overall cell populations were similar across the samples analyzed.

3. If by dissection, then these normal samples harbour a mixed population of luminal and myo/basal cells and a mixed population of ER+ve/-ve cells and so these results showing differential expression of lineage markers, E2 response and cell adhesion markers are great to see as they’d be expected findings in this context. This doesn’t necessarily indicate a cell lineage shift though, more is a consequence of comparing a mixed cell population (normal = basal/luminal cells) and a much more pure cell population (LCIS = luminal cells). A more ideal experiment might be to isolate normal luminal cells for comparison to LCIS. This is a comment/limitation only, not asking for this to be done. But it needs consideration, especially wrt to the discussion paragraph 1 stating “Importantly, gene expression changes we observed in HMEC models were mirrored in clinical samples of LCIS, which presented an increase in luminal markers and decrease in basal markers relative to normal breast tissue, supporting that early CDH1 loss drives cells to luminal lineage phenotype.” The decrease in basal markers is just because of the higher prevalence of basal cells in normal breast epithelium to LCIS. The increase in E2 response also makes sense as the normal epithelial cells are ER in ~10-20% of cells vs 100% of cells in LCIS. Nevertheless this is an important phenotype switch or indicator that the cell of origin is ER+.

We agree with the limitations raised by the reviewer; analyses of isolated cell populations e.g. via single-cell RNA-seq will be an important future direction to address cell type prevalence in these samples. In response to a similar concern from Reviewer 1 (see Point #3), we edited text related to these analyses to stress that the differences observed in LCIS v normal tissues are not directly indicative of the causal impact of CDH1 loss. In particular, the results section to more explicitly address the correlative nature of this analysis and the literature observations on DCIS, now in lines 255-261:

- “Importantly, the observed gene expression differences are correlative with CDH1 loss in early lobular neoplasia, yet may reflect other features of oncogenesis, e.g. increased epithelial cellularity in LCIS. However, the shift we observe in LCIS contrasts data from ductal carcinoma in situ (DCIS) in the literature; Rebbeck et al assessed gene expression in samples from n=145 patients with DCIS, and identified EMT activation in the progression of DCIS to invasive disease [62]. Taken together, LCIS gene expression data are consistent with the changes seen in our CDH1 loss models in HMEC, and support that early ILC oncogenesis includes a cell lineage shift to a luminal phenotype.”

4. Also, Im not sure I agree with the statement ‘This shift in LCIS contrasts ductal carcinoma in situ, which are associated with EMT activation [62].’ I appreciate the data in ref62, but I don’t think it is as clearly different as this brief statement makes it out. DCIS is much more heterogeneous than LCIS. I suspect a comparison between normal epithelium and ER+ DCIS would show the same ‘lineage’ shift (for the same reasons as noted) to luminal and E2 response as LCIS. So maybe take this statement out as its not necessary.

We agree with this point and have edited the comment about this study to de-emphasize luminal/basal remodeling in the DCIS and focus on differential activation of EMT (see response immediately above). We would elect to keep this updated note to provide some context about DCIS, as it is relevant to understand the putative distinct role of CDH1 loss in early breast tumorigenesis.

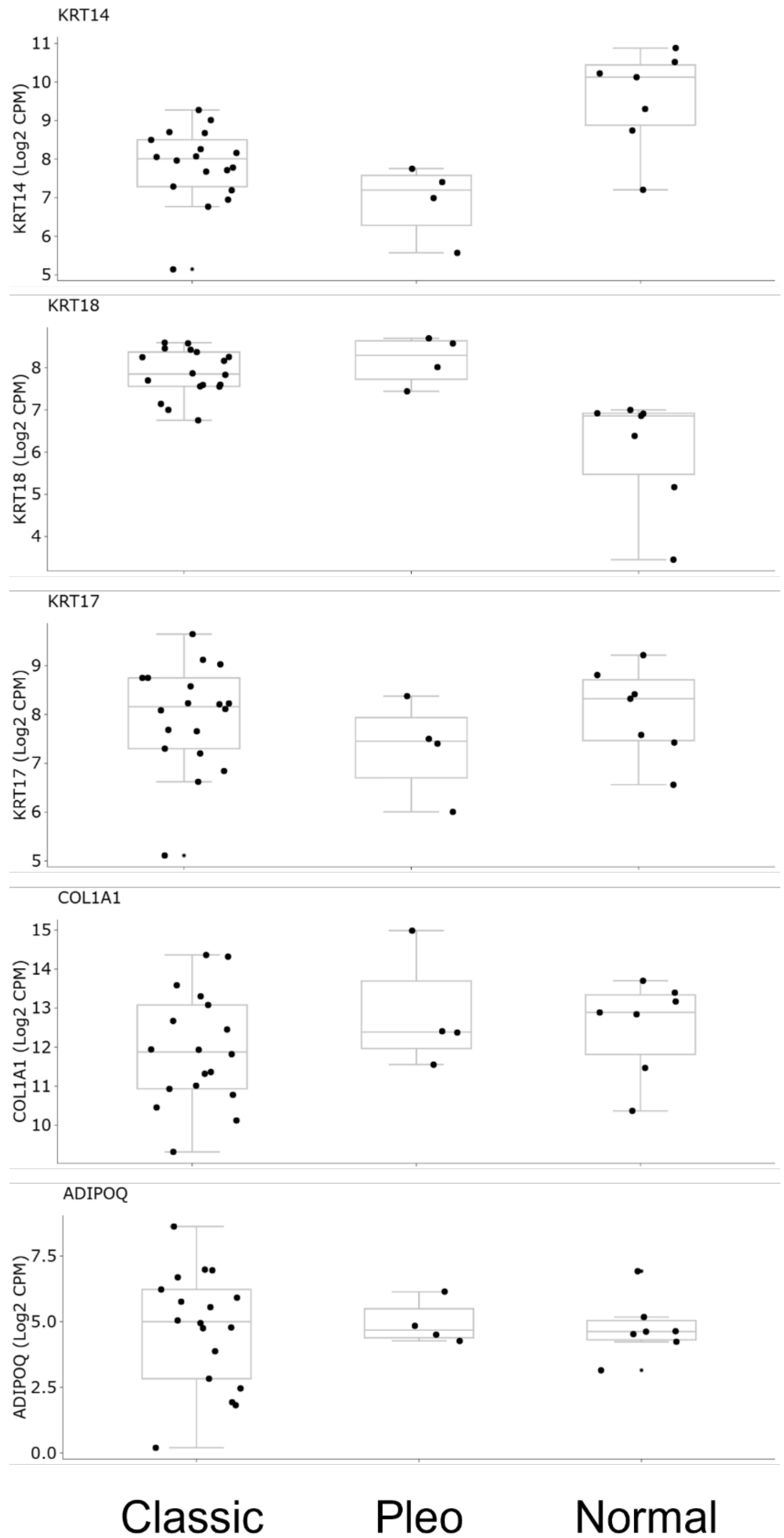

Reviewer Figure 2. Cell type-associated gene expression in LCIS RNAseq.

Section ‘Cellular lineage markers support a CDH1 loss-induced luminal progenitor-like phenotype’. Flow cytometry of HMEC 184D1 pre vs post CDH1-KO, shows a shift from predominantly basal to mixed basal-luminal progenitor phenotype.

In the colony formation assay – how do you define luminal vs basal? Is that by just looking at CD10 expression? Did you also look at Ecadherin expression here to verify the targeted knockdown in the cells/colonies being examined? This would be really useful, esp given the heterogeneous nature of knockdown in the cells; plus is knock down seen in luminal or basal colonies with preference? The images are hard to see– can you give a higher power or cell stained image?

In the early common progenitor-derived colonies assay (PMID: 26544537), colonies are defined as luminal, basal, or mixed based on morphology after seeding on the irradiated fibroblast feeder layer. Luminal progenitors form rounded, tightly packed colonies, while basal progenitors form irregularly shaped colonies with more scattered cells and mixed phenotype progenitors show both features (also see PMID: 19200636). This description has been added to the methods in lines 647-649. Unfortunately we do not have E-cadherin expression data from these colony assays, but have updated Figure 5 with additional higher power images.

Section 'CDH1-KO induces lineage remodeling in both luminal and basal cells'. Important to look at this – the selection against basal cells. This was done in HMEC 184D1 with CDH1-KO. It would have been nice to see how consistently this change was seen across other lines and in ecadherin low vs high cells.

We agree that expansion of these sorting-remodeling studies would be ideal, along with including parallel data on E-cadherin status. As alluded to above, our E-cadherin flow cytometry assay is incompatible with lineage marker assessment as the former requires fixation for the antibody/epitope, limiting out ability to address some related questions at present. However, included below as **Reviewer Figure 3**, we performed an orthogonal experiment to confirm whether CDH1 loss can induce reprogramming in either EpCAM-high or EpCAM-low cells. Using 240D1, which have a higher luminal baseline population, we used siRNA to suppress CDH1, which drove increased CD10 levels in both EpCAM-high and EpCAM-low starting populations, consistent with induction of lineage remodeling and the progenitor phenotype in both luminal and basal cells.

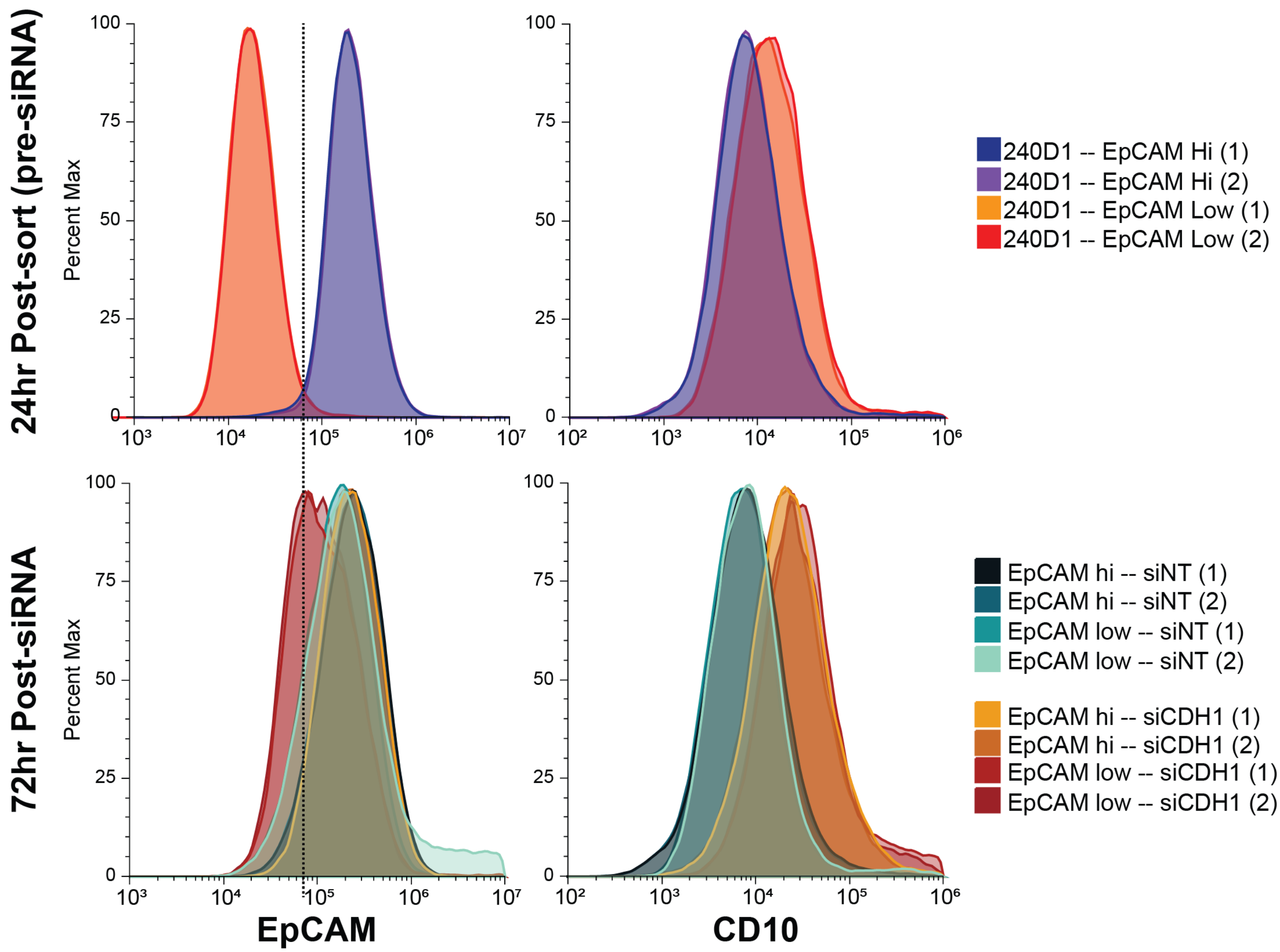

Reviewer Figure 3. Lineage/sorting experiment with 240D1 and siRNA-based CDH1 suppression. (1) and (2) represent biological replicates. Top – Post-sort (by EpCAM level); CD10 is elevated in EpCAM low cells consistent with increased expression in basal epithelium. Bottom – post-siRNA. EpCAM levels reverting to baseline, likely slower in EpCAM-low sort but MFI is above sorting gate (dashed line). CD10 levels are increased in all sorted populations with siCDH1 (i.e. increased CD10 in either EpCAM high or low starting cells) but siNT revert to baseline.

Section 'CDH1 loss induces a temporal shift in lineage marker expression'. Time course to show ecad loss and lineage marker changes along a time course. This was done in HMEC 184D1 with CDH1-KO. This is important to see the timeline of when ecad is downregulated. In line with comments above, is the remaining Ecad expression seen in these blots inferring that KO is complete in some cells but not all or is it just reduced but not completely lost in all cells? Does ALDH actually change much? Looking at the plot the fold change doesn't seem like it will be very much. Is Fig 6F/G the same as SuppFig 9B/C?

We hope that discussion above, and data in Reviewer Figure 1, provide additional context to interpret the efficacy of CDH1 suppression in our models.

Figure 6F/G and SF9B/C each represent one of the 184D1 CDH1-KO models (g2 and g1, respectively), which showed consistent changes in target gene expression after Cas9 induction, supporting the effect of CDH1 loss. We clarified the sources of the data in these panels in line 310-313. In these models, ALDH1A3 mRNA increases 2-3-fold within ~7-10 days post-CDH1-KO.

Section 'CDH1-KO increases proliferative potential and anoikis resistance'. Prolif and anoikis resistance in HMEC 184D1 with CDH1-KO; seems reasonable. I like the data specifically looking at Ecadherin levels in the different cell subsets – this is important. You don't specifically know the genotype of the cells analysed here though (some could be WT, hemizygous WT or homozygous null) so I don't think you can specifically say some cells are CDH1 hemizygous and Ecadherin positive from this data. The positive cells could be WT?

Consistent with the above discussion, we agree with this point and have softened the description of E-cadherin positive cells in line 338-340: "A subset of CDH1-KO cells remain E-cadherin positive (e.g. hemizygous for CDH1), while a subset of HMEC as in NTG controls are E-cadherin negative (likely via reduced CDH1/E-cadherin in basal cells [68])."
