# Supplementary material for: CDH1 loss remodels gene expression and lineage identity in human mammary epithelial cells
Source: PLoS Genet. 2026 Oct 27;22(10):e1012351. doi: 10.1371/journal.pgen.1012351 (PMC13722294; doi:10.1371/journal.pgen.1012351)
Supplement: Peer review history

# Peer review: Decision Letter 0

**DOI:** 10.1371/journal.pgen.1012351.r001

**Authors:** Hongbin Ji, Andrew Hong

17 Jul 2026

PGENETICS-D-26-00592

CDH1 loss remodels gene expression and lineage identity in human mammary epithelial cells

PLOS Genetics

Dear Dr. Musick,

Thank you for submitting your manuscript to PLOS Genetics. As you can see from the two reviewers, there is a lot of enthusiasm for this manuscript. After careful consideration, we feel that it has merit but does not fully meet PLOS Genetics's publication criteria as it currently stands. Therefore, we invite you to submit a revised version of the manuscript that addresses the points raised during the review process.

Please submit your revised manuscript by 11/15/2026. If you will need more time than this to complete your revisions, please reply to this message or contact the journal office at plosgenetics@plos.org. When you're ready to submit your revision, log on to https://www.editorialmanager.com/pgenetics/ and select the 'Submissions Needing Revision' folder to locate your manuscript file.

Please include the following items when submitting your revised manuscript:

* A letter that responds to each point raised by the editor and reviewer(s). You should upload this letter as a separate file labeled 'Response to Reviewers'. This file does not need to include responses to formatting updates and technical items listed in the 'Journal Requirements' section below.

* A marked-up copy of your manuscript that highlights changes made to the original version. You should upload this as a separate file labeled 'Revised Manuscript with Track Changes'.

* An unmarked version of your revised paper without tracked changes. You should upload this as a separate file labeled 'Manuscript'.

If you would like to make changes to your financial disclosure, competing interests statement, or data availability statement, please make these updates within the submission form at the time of resubmission. Guidelines for resubmitting your figure files are available below the reviewer comments at the end of this letter.

As the corresponding author, your ORCID iD is verified in the submission system and will appear in the published article. PLOS supports the use of ORCID, and we encourage all coauthors to register for an ORCID iD and use it as well. Please encourage your coauthors to verify their ORCID iD within the submission system before final acceptance, as unverified ORCID iDs will not appear in the published article. Only the individual author can complete the verification step; PLOS staff cannot verify ORCID iDs on behalf of authors.

We look forward to receiving your revised manuscript.

Kind regards,

Andrew Lee Hong

Academic Editor

PLOS Genetics

Aimée Dudley

Editor-in-Chief

PLOS Genetics

Anne Goriely

Editor-in-Chief

PLOS Genetics

**Additional Editor Comments (if provided):**

**Journal Requirements:**

If the reviewer comments include a recommendation to cite specific previously published works, please review and evaluate these publications to determine whether they are relevant and should be cited. There is no requirement to cite these works unless the editor has indicated otherwise.

1) We ask that a manuscript source file is provided at Revision. Please upload your manuscript file as a .doc, .docx, .rtf or .tex. If you are providing a .tex file, please upload it under the item type u2018LaTeX Source Fileu2019 and leave your .pdf version as the item type u2018Manuscriptu2019.

2) Please provide an Author Summary. This should appear in your manuscript between the Abstract (if applicable) and the Introduction, and should be 150-200 words long. The aim should be to make your findings accessible to a wide audience that includes both scientists and non-scientists. Sample summaries can be found on our website under Submission Guidelines:

https://journals.plos.org/plosgenetics/s/submission-guidelines#loc-parts-of-a-submission

3) Please upload all main figures as separate Figure files in .tif or .eps format. For more information about how to convert and format your figure files please see our guidelines:

https://journals.plos.org/plosgenetics/s/figures

4) Please upload a copy of Figures FIG 1 A-D, 2A-I, 3A-I, 4A-C, 5A-G, 6A-G, and 7A-D which you refer to in your text on pages 11, 13, 15, 17, 19, 20, and 23. Or, if the figure is no longer to be included as part of the submission please remove all reference to it within the text.

5) Please amend your detailed Financial Disclosure statement. This is published with the article. It must therefore be completed in full sentences and contain the exact wording you wish to be published.

i) State what role the funders took in the study. If the funders had no role in your study, please state: "The funders had no role in study design, data collection and analysis, decision to publish, or preparation of the manuscript."

ii) If any authors received a salary from any of your funders, please state which authors and which funders..

If you did not receive any funding for this study, please simply state: u201cThe authors received no specific funding for this work.u201d

6)Please send a completed 'Competing Interests' statement, including any COIs declared by your co-authors. If you have no competing interests to declare, please state "The authors have declared that no competing interests exist". Otherwise please declare all competing interests beginning with the statement "I have read the journal's policy and the authors of this manuscript have the following competing interests

7)We have amended your Competing Interest statement to comply with journal style. We kindly ask that you double check the statement and let us know if anything is incorrect,

**Reviewers' comments:**

Reviewer's Responses to Questions

**Comments to the Authors:**

**Please note here if the review is uploaded as an attachment.**

Reviewer #1: Reviewer Comments to the Authors

PLOS Genetics | PGENETICS-D-26-00592

“CDH1 loss remodels gene expression and lineage identity in human mammary epithelial cells”

Summary

Musick and colleagues address an important and understudied question in breast cancer biology: how the early, defining genetic event of invasive lobular carcinoma (loss of CDH1/E-cadherin) contributes to transformation, and whether the biological consequences of CDH1 loss differ depending on the mode of E-cadherin disruption. This is a timely and significant question for the ILC field, where CDH1 loss is nearly universal and occurs early, yet the mechanisms by which it creates a permissive or advantageous state for lobular oncogenesis remain incompletely understood.

Using a panel of finite-lifespan, post-stasis human mammary epithelial cell models that retain luminal/basal heterogeneity, the authors compare three approaches to E-cadherin targeting: extracellular antibody inhibition, transient CDH1 knockdown, and inducible CRISPR/Cas9-mediated CDH1 knockout. The central finding is that the mode of E-cadherin targeting matters. Antibody-mediated disruption of cell-cell contacts induces EMT-associated transcriptional programs, whereas genetic CDH1 loss represses EMT signatures and shifts cells toward a luminal, and more specifically luminal progenitor-like, phenotype. The authors support this model using a broad and impressive set of approaches, including bulk RNA-seq, single-cell RNA-seq, ATAC-seq, analysis of clinical LCIS samples, flow cytometry, colony-forming assays, lineage-sorting experiments, proliferation assays, and anoikis-resistance assays.

Overall, this is a carefully constructed and conceptually important study. The manuscript advances a provocative but well-supported reframing of CDH1 loss in early ILC biology: rather than functioning simply as an EMT-like loss of epithelial adhesion, genetic CDH1 loss appears to remodel mammary epithelial lineage identity and create a luminal progenitor-like state with increased proliferative and survival capacity. The work should be of broad interest to investigators studying ILC, mammary epithelial lineage biology, and tumor initiation.

My comments below are intended primarily to clarify the strength of the central claims, improve integration across models and datasets, and ensure that causal language is appropriately calibrated.

Major Comments

1. The antibody-versus-genetic comparison is central and should be more carefully framed.

A major strength of the manuscript is the comparison between functional E-cadherin disruption by antibody inhibition and genetic CDH1 loss by siRNA or CRISPR/Cas9. This comparison underlies one of the most important conclusions of the paper: that antibody-mediated disruption of cell-cell contacts induces EMT, whereas genetic CDH1 loss represses EMT and promotes luminal lineage remodeling.

This is a compelling concept, but the comparison carries some inherent differences that should be acknowledged more explicitly. The antibody treatment is acute, whereas the genetic perturbations occur over different timescales, especially in the two-week inducible CDH1-KO system. Thus, the observed differences may reflect not only the mode of E-cadherin targeting but also the distinction between acute junctional disruption and longer-term adaptation to genetic CDH1 loss.

In addition, the Methods indicate that the HECD-1 antibody analysis was complicated by an unexpected response to the IgG control, leading the authors to derive HECD-1-specific DEGs by subtracting the IgG-versus-control gene list from the HECD-1-versus-control gene list. This is a reasonable analytical solution, but readers would benefit from seeing the magnitude and nature of the IgG response more directly.

Specifically, I suggest that the authors:

• Acknowledge more explicitly that antibody-mediated E-cadherin disruption is acute, whereas siRNA- and CRISPR/Cas9-mediated CDH1 loss occur over different and longer timescales.

• Clarify that the observed differences may reflect both the mode of E-cadherin targeting and the time allowed for cellular adaptation.

• Provide, perhaps in the supplement, the number of IgG-responsive genes and the major pathways enriched in the IgG-versus-control comparison.

• Temper language that presents the antibody and genetic arms as simple binary opposites, and instead frame the findings as evidence that acute E-cadherin contact disruption and genetic CDH1 loss produce distinct cellular states.

I do not think this issue undermines the core conclusion, especially because the genetic CDH1-loss data are supported by multiple orthogonal approaches. However, addressing these points would make the central comparison clearer and more convincing.

2. The cross-model and cross-method structure should be made more explicit.

One of the manuscript’s strengths is its breadth. However, because different experimental arms are performed in different HMEC models and with different perturbation strategies, the reader is sometimes asked to integrate findings across several systems. For example, the single-cell RNA-seq and much of the phenotypic work are centered on CDH1-KO in 184D1 cells, whereas the ATAC-seq is performed after siRNA-mediated CDH1 knockdown in 240D1 and 153D1 cells. The antibody comparison is performed in another subset of models.

This is not necessarily a weakness; indeed, concordance across multiple HMEC backgrounds supports the generality of the findings. However, the manuscript would benefit from a clearer summary of which conclusions are supported in which model and by which perturbation.

To improve clarity, I suggest that the authors:

• Add a summary table or schematic indicating which conclusions are supported by which HMEC model, perturbation strategy, and assay.

• For each major claim, indicate whether it is supported by antibody inhibition, siRNA knockdown, CRISPR/Cas9 knockout, bulk RNA-seq, single-cell RNA-seq, ATAC-seq, flow cytometry, sorting, or functional assays.

• Clarify when findings are robust across multiple starting lineage states versus inferred primarily from one model.

• More explicitly discuss how baseline luminal/basal composition differs among HMEC models, particularly the relatively luminal baseline state of 240D1 cells versus the more basal-enriched baseline state of 184D1 cells.

• If existing data permit, provide a targeted concordance check between the ATAC-derived transcriptional program and the 184D1 CDH1-KO model that carries much of the functional weight.

• For example, the authors could show whether the NFI/KLF/TFAP2C-associated program or related candidate transcriptional modules identified from ATAC-seq are similarly induced in the 184D1 CDH1-KO system.

Such a table or schematic would help readers track the evidentiary basis for each conclusion and better integrate the chromatin, transcriptomic, and phenotypic arms of the study.

3. The LCIS clinical analysis is valuable but should be interpreted as supportive rather than causal.

The LCIS RNA-seq analysis is a valuable addition, particularly because clinical transcriptomic datasets of lobular precursor lesions are limited. The finding that LCIS samples show increased luminal-associated genes and decreased basal/EMT-associated genes provides an important clinical parallel to the HMEC models.

However, this analysis is necessarily correlative. The comparison is between LCIS, which is already E-cadherin-negative and neoplastic, and normal breast tissue. Thus, the luminal signature could reflect the intrinsic luminal/ER-positive nature of lobular neoplasia, enrichment for neoplastic epithelium, differences in epithelial composition, or tissue-composition differences between LCIS and reduction mammoplasty specimens, rather than being directly attributable to CDH1 loss alone.

I suggest that the authors:

• Frame the LCIS analysis as consistent with the HMEC model rather than as independent causal evidence that CDH1 loss drives luminal reprogramming in vivo.

• Distinguish clearly between the causal HMEC perturbation experiments and the correlative clinical LCIS comparison.

• Provide additional information about the LCIS cohort where possible, including ER status, patient age, lesion type, classic versus pleomorphic status, and an estimate of epithelial/neoplastic cellularity.

• Discuss epithelial fraction or tissue-composition differences between LCIS and normal breast samples if such information is available.

• Clarify that comparisons to DCIS and EMT activation in ductal lesions are literature-based unless DCIS is analyzed directly in the same framework.

This distinction is important because the HMEC perturbation experiments are the causal arm of the study, whereas the LCIS dataset provides clinical concordance. The LCIS data are still valuable, but the interpretation should be appropriately calibrated.

4. Reprogramming versus selection should be discussed carefully in the proliferation data.

The population-doubling data support the idea that CDH1 loss provides a growth or persistence advantage in finite-lifespan HMEC cultures. This is highly relevant to ILC initiation, where early CDH1-deficient cells must persist and expand despite loss of epithelial adhesion.

However, because the CDH1-KO cultures are mixed populations, the increased population doublings could reflect a cell-autonomous proliferative effect of CDH1 loss, selective outgrowth of edited/E-cadherin-low cells, or both. This distinction bears directly on the authors’ model of CDH1 loss as a lineage-remodeling event that provides clonal advantage.

To clarify this point, I suggest that the authors:

• Explicitly acknowledge that increased population doublings in mixed KO cultures may reflect a combination of lineage reprogramming and selection.

• Clarify whether the E-cadherin-negative or edited fraction changes over serial passage, if such data are already available.

• If feasible with existing material or straightforward follow-up experiments, track the fraction of E-cadherin-negative or edited cells over serial passage.

• If the E-cadherin-negative fraction increases over time, discuss this as support for a selection model.

• If the E-cadherin-negative fraction remains stable while the population expands more robustly, discuss this as support for a more cell-autonomous proliferative effect.

• Clarify the statistical tests used in the population-doubling analysis, as the figure legend appears to refer to both slope comparisons and ANOVA-based comparisons.

I do not view serial tracking as essential if the authors instead clearly acknowledge this limitation in the Discussion. Either interpretation (selection, reprogramming, or a combination of both) would be interesting and relevant to the authors’ model.

5. The lineage-sorting experiment is a major strength and should be emphasized, but reporting details should be expanded.

The lineage-sorting experiment is one of the strongest parts of the manuscript because it directly addresses a key alternative explanation: that the apparent luminal shift after CDH1 loss reflects selective expansion of pre-existing luminal cells rather than remodeling of lineage state. The finding that both sorted luminal and sorted basal populations converge toward an EpCAM-high state after CDH1-KO is important and should be highlighted more prominently in the Results and Discussion.

This experiment is especially important because it is the strongest evidence in the manuscript against a purely compositional-selection explanation for the luminal shift. Because it carries substantial interpretive weight, the authors should provide more methodological and quantitative detail.

Specifically, I suggest that the authors clarify:

• The number of independent sorting experiments performed.

• The number of biological and technical replicates for each sorted population.

• The sort purity for luminal and basal fractions.

• How the flow-cytometry gates were defined.

• Whether gates were based on FMO controls, unstained controls, prior literature, or another strategy.

• Why the reported n appears to differ across panels in Fig. 6.

• Whether the sort-efficiency check, which appears to be n = 1, was intended as a representative validation rather than a replicated experiment.

Similarly, for the colony-forming/progenitor assays in Fig. 5, please report:

• The number of colonies scored.

• The number of independent experiments.

• Whether colony phenotypes were scored blinded.

• The statistical test used for phenotype proportions.

• Whether representative images correspond to quantified experiments.

These assays provide important functional support for the luminal progenitor-like interpretation, so transparent reporting will strengthen the manuscript.

6. The ILC-specific model would benefit from clearer distinction between instructive, selective, and permissive roles of CDH1 loss.

The manuscript makes a compelling case that genetic CDH1 loss can remodel HMEC lineage identity toward a luminal progenitor-like state. However, because ILC is overwhelmingly luminal/ER-positive and CDH1 loss is early but not sufficient for full malignant transformation, it would be helpful for the Discussion to distinguish among several related possibilities.

The authors should discuss whether CDH1 loss may:

• Instruct a luminal progenitor-like state.

• Select for cells already compatible with luminal/ER-associated programs.

• Stabilize or permissively expand a luminal progenitor-like state.

• Require cooperation with additional ILC-associated events, such as PI3K pathway alteration, ER pathway activity, CCND1 gain, or other lesions.

This distinction would make the model more nuanced and more directly anchored in ILC biology. The use of CCND1-overexpressing post-stasis HMECs is appropriate and relevant, particularly given the frequency of CCND1 gain in ILC, but it also means that the effects of CDH1 loss are being studied in a partially primed hyperplastic context rather than in fully naïve mammary epithelium.

I suggest that the authors:

• Discuss the CCND1-overexpressing post-stasis HMEC context as an important feature of the model rather than as a weakness.

• Clarify how this model relates to early lobular oncogenesis.

• Avoid implying that CDH1 loss alone is sufficient for full malignant transformation.

• More explicitly state that CDH1 loss may create a permissive or advantageous lineage state that requires additional oncogenic events.

Minor Comments

1. Data availability. The statement indicates that RNA-seq and ATAC-seq datasets are being uploaded to GEO. Because these sequencing datasets are central to the manuscript’s claims, GEO accession numbers and reviewer tokens should be provided during revision or before acceptance. This should include:

• Bulk RNA-seq datasets.

• ATAC-seq datasets.

• Single-cell RNA-seq datasets.

• LCIS RNA-seq datasets, as applicable.

• Supplemental data files required to reproduce the analyses.

2. Supplementary figure cross-references. Please check the cross-references for FMO gating controls. The text and figure legends appear to refer to different supplementary figure numbers.

3. Cell-line nomenclature. Please standardize HMEC line names throughout the manuscript. For example, 184D-D1L, 184D1, 240L-D1, 240D1, and possible variants in the Methods should be harmonized or clearly defined at first use.

4. Define terms for a broad genetics readership. Terms such as “post-stasis,” “stasis,” “NTG,” and “M87A medium” should be defined at first mention or briefly cited.

5. ID2 model. The Discussion presents an interesting model involving ID2 and luminal progenitor-like remodeling. This is compelling, but the functional role of ID2 is not directly tested in the current manuscript. Please frame ID2 as a strong candidate or future direction unless additional functional data are added.

6. Cell-cycle/proliferation validation. The single-cell analysis suggests increased S/G2/M fractions after CDH1 loss. If available, an orthogonal readout such as EdU, Ki67, or cell-cycle flow analysis would strengthen the connection between the single-cell data and the population-doubling assay. If not available, the computational nature of the cell-cycle inference should simply be stated.

7. Pleomorphic LCIS. The handling of pleomorphic LCIS samples should be stated clearly in the Results where the LCIS analysis is introduced. Given the small number of pLCIS cases, exclusion after clustering may be appropriate, but the rationale should be transparent.

8. Statistical thresholds. Different adjusted p-value or q-value thresholds are used across panels. Please include a brief statement in the Methods explaining the rationale for panel-specific thresholds and the correction methods used.

9. Motif-analysis thresholds. The motif analysis appears to use different NES thresholds for gained versus lost peaks. Please justify the asymmetric thresholds or state whether the qualitative conclusions are robust to matched thresholds.

10. Anoikis assay controls. The anoikis-resistance assay is highly relevant to ILC biology and is one of the manuscript’s strongest functional links. Please state viability at seeding and clarify whether any additional anoikis-sensitive or positive-control condition was included. If not, this could be acknowledged.

11. Typographic/reference cleanup. Please check reference formatting and minor typographic issues. For example, one DOI/reference string appears malformed, and “preformed” should likely be “performed.” Also, please confirm that all figure panels render correctly in the review PDF.

12. Claims calibration. The Abstract is appropriately hedged in stating that CDH1 loss “may” underpin mechanisms of early ILC oncogenesis. Please ensure that similarly calibrated language is used throughout the Results and Discussion, especially when interpreting the LCIS clinical dataset.

Reviewer #2: Nice paper addressing an important area of oncogenesis for invasive lobular carcinoma. Most research has focused on studying cancer cells to try and understand tumour biology and behaviour, whereas the present study asks questions about the very origins of lobular neoplasia. We understand that ILC is epithelial in nature and does not show evidence of EMT, as in other scenarios related to ecadhein deficiency; but not how the mode of action targeting the CDH1 gene plays a key role in delineating cell lineage. Thus current study presents an elegant experimental plan to demonstrate that CDH1 inactivation by mutation (‘neither transient nor reversible’) drives a luminal epithelial phenotype with elevated proliferative capacity. This is a large body of work, the paper is well written, figures and data well described/referenced.

There are a few limitations to the work. This is not to diminish the work done nor to ask for more, but to be acknowledged in the manuscript. Multiple assays are done through this paper, using eg just 1 of the HMEC models with one of the CDH1 manipulations, and these are not always consistent. Most of the assays were done with HMEC 184D1, where CDH1 was genetically knocked out, which is good as it re-capitulates the cancer better, but this is just one line and so some caution required.

The heterogenous HMEC cell populations (mix of luminal and basal) makes some of the interpretation tricky; with the added complexity that the manipulation of CDH1 is not 100% in the lines such that the cell populations are a mix of lineage and a mix of ecad expression, so the data is not necessarily ‘clean’ (but reflects biology with studying these things in situ). Its therefore not clear if the results directly relate to the loss of CDH1. In this respect I like the data in later figures where Ecadhein expression itself is investigated (in the timeline Fig6F/G; and in the Ecad gating in Fig 7). But this raises a further question about the manipulation of ecad by the different methods:

For siRNA – knock down is incomplete, as is often the case (SuppFig 1B), but do you know if some cells are negative and some are positive here or that all have reduced expression?

For CDH1-KO – this yielded 30-50% of ecad negative cells (SuppFig1D), but is this actually negative or low? looking at the scale on the flow plots, this could be ‘low’ when compared to the first plot of unstained cells; so does this mean that cells still express low levels of ecad or is there a mixed pop of negative, low and unchanged ecad?

Comments on Results

Section ‘Genetic loss of E-cadherin represses EMT and shifts cells to a luminal transcriptional state’

Nice data here showing the different modes of ecadherin abrogation and the characterisation of these models by RNAseq.

1. It would be good to add some quantification for the proportion of reduced adhesion and the % knockdown for each line in Supp Fig 1A and B

2. to just clarify - RNAseq was performed on parental and Ecad manipulated cells for each line and each model; and the DEGs were identified between parental vs manipulated and then compared by venn diagrams?

Section: ‘Single cell analysis supports CDH1-KO-induced reprogramming in luminal and basal cells’. Single cell seq analysis was conducted on 1 HMEC line (184D1) with 1 model of Ecadherin abrogation (KO). Most cells in this line are basal to start with. A mild shift to increase the proportion of cells being ‘luminal’ and proliferating was observed.

1. Figure 2 – can we see an EMT score in this figure, similar to how you’ve shown cell cycle/cell identity.

2. Given the loss of Ecadherin is heterogeneous in these cells (SuppFig1B) is it possible to see the profile of cells that are Ecadherin negative post knockdown? How do those cells specifically compare to Ecadherin +ve cells of the same phenotype? Ie if Ecadherin loss is in the CDH1-KO luminal cells – can you compare these cells to both Ecadherin-positive luminal NTG cells and CDH1-KO cells for the same features as described (EMT, cell cycle, phenotype markers)?

3. Note legend for SuppFig6 is referencing part B and C the wrong way around.

4. How does the cholesterol hallmark come about? Im missing the link here.

Section: ‘Chromatin landscape supports CDH1 loss-induced changes in cell identity’. Here 2 siRNA HMEC lines were used (240D1 and 153D1) for whole cell population ATAC-seq. ie these are different lines to the line and mode of CDH1 manipulation used in scRNAseq. A small proportion of peaks were changed by siRNA KD, which is perhaps expected. Nevertheless, this still translated to many genes and quite a few changes in common across the 2 lines that had corresponding changes in expression, replicated in all models to some degree, including interesting genes.

Given the heterogeneity of these cell populations (as noted above - mixed populations of basal and luminal cells, mixed populations of Ecadherin negative vs positive cells), could some signal be masked?

Section ‘CDH1 loss in HMEC mirrors gene expression changes in early lobular neoplasia’. 22 LCIS from the archives were analysed by RNAseq against 7 ‘normals’.

1. How was the data quality for these samples, given their age and FFPE nature? Data described certainly looks sensible.

2. How were the 7 control samples prepared? Presumably normal ducts & lobules were dissected to enrich for normal epithelia to match the enrichment for epithelia in the LCIS samples?

3. If by dissection, then these normal samples harbour a mixed population of luminal and myo/basal cells and a mixed population of ER+ve/-ve cells and so these results showing differential expression of lineage markers, E2 response and cell adhesion markers are great to see as they’d be expected findings in this context. This doesn’t necessarily indicate a cell lineage shift though, more is a consequence of comparing a mixed cell population (normal = basal/luminal cells) and a much more pure cell population (LCIS = luminal cells). A more ideal experiment might be to isolate normal luminal cells for comparison to LCIS. This is a comment/limitation only, not asking for this to be done. But it needs consideration, especially wrt to the discussion paragraph 1 stating “Importantly, gene expression changes we observed in HMEC models were mirrored in clinical samples of LCIS, which presented an increase in luminal markers and decrease in basal markers relative to normal breast tissue, supporting that early CDH1 loss drives cells to luminal lineage phenotype.” The decrease in basal markers is just because of the higher prevalence of basal cells in normal breast epithelium to LCIS. The increase in E2 response also makes sense as the normal epithelial cells are ER in ~10-20% of cells vs 100% of cells in LCIS. Nevertheless this is an important phenotype switch or indicator that the cell of origin is ER+.

4. Also, Im not sure I agree with the statement ‘This shift in LCIS contrasts ductal carcinoma in situ, which are associated with EMT activation [62].’ I appreciate the data in ref62, but I don’t think it is as clearly different as this brief statement makes it out. DCIS is much more heterogeneous than LCIS. I suspect a comparison between normal epithelium and ER+ DCIS would show the same ‘lineage’ shift (for the same reasons as noted) to luminal and E2 response as LCIS. So maybe take this statement out as its not necessary.

Section ‘Cellular lineage markers support a CDH1 loss-induced luminal progenitor-like phenotype’. Flow cytometry of HMEC 184D1 pre vs post CDH1-KO, shows a shift from predominantly basal to mixed basal-luminal progenitor phenotype.

In the colony formation assay – how do you define luminal vs basal? Is that by just looking at CD10 expression? Did you also look at Ecadherin expression here to verify the targeted knockdown in the cells/colonies being examined? This would be really useful, esp given the heterogeneous nature of knockdown in the cells; plus is knock down seen in luminal or basal colonies with preference? The images are hard to see that these are actually cells – can you give a higher power or cell stained image?

Section ‘CDH1-KO induces lineage remodeling in both luminal and basal cells’. Important to look at this – the selection against basal cells. This was done in HMEC 184D1 with CDH1-KO. It would have been nice to see how consistently this change was seen across other lines and in ecadherin low vs high cells.

Section ‘CDH1 loss induces a temporal shift in lineage marker expression’. Time course to show ecad loss and lineage marker changes along a time course. This was done in HMEC 184D1 with CDH1-KO. This is important to see the timeline of when ecad is downregulated. In line with comments above, is the remaining Ecad expression seen in these blots inferring that KO is complete in some cells but not all or is it just reduced but not completely lost in all cells? Does ALDH actually change much? Looking at the plot the fold change doesn’t seem like it will be very much. Is Fig 6F/G the same as SuppFig 9B/C?

Section ‘CDH1-KO increases proliferative potential and anoikis resistance’. Prolif and anoikis resistance in HMEC 184D1 with CDH1-KO; seems reasonable. I like the data specifically looking at Ecadherin levels in the different cell subsets – this is important. You don’t specifically know the genotype of the cells analysed here though (some could be WT, hemizygous WT or homozygous null) so I don’t think you can specifically say some cells are CDH1 hemizygous and Ecadherin positive from this data. The positive cells could be WT?

# Author comment: Author response to Decision Letter 1

**DOI:** 10.1371/journal.pgen.1012351.r002

15 Sep 2026

Attachment Submitted filename: Musick PLoS Gen_Response to Reviewer Comments v1.2.pdf

# Peer review: Decision Letter 1

**DOI:** 10.1371/journal.pgen.1012351.r003

**Authors:** Hongbin Ji, Andrew Hong

13 Oct 2026

Dear Dr Sikora,

We are pleased to inform you that your manuscript entitled "*CDH1*  loss remodels gene expression and lineage identity in human mammary epithelial cells" has been editorially accepted for publication in PLOS Genetics. Congratulations!

Before your submission can be formally accepted and sent to production you will need to

1) complete our formatting changes, which you will receive in a follow up email and

2) address the very minor (but important for clarity) points raised below by the reviewer

Please be aware that it may take several days for you to receive this email; during this time no action is required by you. Please note: the accept date on your published article will reflect the date of this provisional acceptance, but your manuscript will not be scheduled for publication until the required changes have been made.

Once your paper is formally accepted, an uncorrected proof of your manuscript will be published online ahead of the final version, unless you’ve already opted out via the online submission form. If, for any reason, you do not want an earlier version of your manuscript published online or are unsure if you have already indicated as such, please let the journal staff know immediately at plosgenetics@plos.org.

In the meantime, please log into Editorial Manager at https://www.editorialmanager.com/pgenetics/, click the "Update My Information" link at the top of the page, and update your user information to ensure an efficient production and billing process. Note that PLOS requires an ORCID iD for all corresponding authors. Therefore, please ensure that you have an ORCID iD and that it is validated in Editorial Manager. To do this, go to ‘Update my Information’ (in the upper left-hand corner of the main menu), and click on the Fetch/Validate link next to the ORCID field.  This will take you to the ORCID site and allow you to create a new iD or authenticate a pre-existing iD in Editorial Manager.

If you have a press-related query, or would like to know about making your underlying data available (as you will be aware, this is required for publication), please see the end of this email. If your institution or institutions have a press office, please notify them about your upcoming article at this point, to enable them to help maximise its impact. Inform journal staff as soon as possible if you are preparing a press release for your article and need a publication date.

Thank you again for supporting open-access publishing; we are looking forward to publishing your work in PLOS Genetics!

Yours sincerely,

Andrew Lee Hong

Academic Editor

PLOS Genetics

Hongbin Ji

Section Editor

PLOS Genetics

Aimée Dudley

Editor-in-Chief

PLOS Genetics

Anne Goriely

Editor-in-Chief

PLOS Genetics

www.plosgenetics.org

BlueSky: @plos.bsky.social

----------------------------------------------------

Comments from the reviewers (if applicable):

Reviewer's Responses to Questions

**Comments to the Authors:**

**Please note here if the review is uploaded as an attachment.**

Reviewer #1: The authors have addressed my concerns satisfactorily, and the revised manuscript is substantially improved. The distinctions between acute E-cadherin disruption and genetic CDH1 loss are clearer, the LCIS findings are appropriately framed as correlative, and the expanded Discussion provides a more balanced interpretation of lineage remodeling and selection. I support publication following correction of a few remaining reporting and typographical issues. I do not think additional experiments are needed.

Please reconcile the LCIS sample numbers. The Results describe 18 classic and 4 pleomorphic LCIS samples, whereas the Methods report successful sequencing from 19 classic and 3 pleomorphic samples. Please confirm the numbers used in the analyses.

Please make the reporting of the Figure 6 sorting experiment consistent across the Methods, figure legend, and response. The descriptions differ regarding biological duplicates versus triplicates at two weeks and pooling versus technical replication at four weeks. Please clearly identify what each reported n represents.

Please correct the figure references. The flow-sorting Methods refer to Figure 5 rather than Figure 6, and the temporal-shift Results cite Figure 5B for EPCAM and ITGA6 expression changes rather than Figure 6G.

In the Discussion, “CCDN1-overexpressing” should read “CCND1-overexpressing,” and “upon CDH1” in the ATAC-seq discussion should read “upon CDH1 loss.”

Reviewer #2: Thank you for considering the comments and providing clarification on these points.

**********

**Have all data underlying the figures and results presented in the manuscript been provided?**

Large-scale datasets should be made available via a public repository as described in the *PLOS Genetics*  data availability policy, and numerical data that underlies graphs or summary statistics should be provided in spreadsheet form as supporting information.

Reviewer #1: Yes

Reviewer #2: Yes

**********

PLOS authors have the option to publish the peer review history of their article (what does this mean?). If published, this will include your full peer review and any attached files.

If you choose “no”, your identity will remain anonymous but your review may still be made public.

**Do you want your identity to be public for this peer review?**  For information about this choice, including consent withdrawal, please see our Privacy Policy.

Reviewer #1: No

Reviewer #2: No

----------------------------------------------------

**Data Deposition**

If you have submitted a Research Article or Front Matter that has associated data that are not suitable for deposition in a subject-specific public repository (such as GenBank or ArrayExpress), one way to make that data available is to deposit it in the Dryad Digital Repository. As you may recall, we ask all authors to agree to make data available; this is one way to achieve that. A full list of recommended repositories can be found on our website.

The following link will take you to the Dryad record for your article, so you won't have to re‐enter its bibliographic information, and can upload your files directly:

http://datadryad.org/submit?journalID=pgenetics&manu=PGENETICS-D-26-00592R1

More information about depositing data in Dryad is available at http://www.datadryad.org/depositing. If you experience any difficulties in submitting your data, please contact help@datadryad.org for support.

Additionally, please be aware that our data availability policy requires that all numerical data underlying display items are included with the submission, and you will need to provide this before we can formally accept your manuscript, if not already present.

----------------------------------------------------

**Press Queries**

If you or your institution will be preparing press materials for this manuscript, or if you need to know your paper's publication date for media purposes, please inform the journal staff as soon as possible so that your submission can be scheduled accordingly. Your manuscript will remain under a strict press embargo until the publication date and time. This means an early version of your manuscript will not be published ahead of your final version. PLOS Genetics may also choose to issue a press release for your article. If there's anything the journal should know or you'd like more information, please get in touch via plosgenetics@plos.org.

# Editor report: Acceptance letter

**DOI:** 10.1371/journal.pgen.1012351.r004

**Authors:** Hongbin Ji, Andrew Hong

PGENETICS-D-26-00592R1

*CDH1*  loss remodels gene expression and lineage identity in human mammary epithelial cells

Dear Dr Sikora,

We are pleased to inform you that your manuscript entitled "*CDH1*  loss remodels gene expression and lineage identity in human mammary epithelial cells" has been formally accepted for publication in PLOS Genetics! Your manuscript is now with our production department and you will be notified of the publication date in due course.

The corresponding author will soon be receiving a typeset proof for review, to ensure errors have not been introduced during production. Please review the PDF proof of your manuscript carefully, as this is the last chance to correct any errors. Please note that major changes, or those which affect the scientific understanding of the work, will likely cause delays to the publication date of your manuscript.

Soon after your final files are uploaded, unless you have opted out or your manuscript is a front-matter piece, the early version of your manuscript will be published online. The date of the early version will be your article's publication date. The final article will be published to the same URL, and all versions of the paper will be accessible to readers.

For Research Articles, you will receive an invoice from PLOS for your publication fee after your manuscript has reached the completed accept phase. If you receive an email requesting payment before acceptance or for any other service, this may be a phishing scheme. Learn how to identify phishing emails and protect your accounts at https://explore.plos.org/phishing.

Thank you again for supporting PLOS Genetics and open-access publishing. We are looking forward to publishing your work!

With kind regards,

Sharmila Kamatchi

PLOS Genetics

On behalf of:

The PLOS Genetics Team

Carlyle House, Carlyle Road, Cambridge CB4 3DN | United Kingdom

plosgenetics@plos.org | +44 (0) 1223-442823

plosgenetics.org | Twitter: @PLOSGenetics
